# First-in-Class Selenium-Containing Potent Serotonin Receptor 5-HT_6_ Agents with a Beneficial Neuroprotective Profile against Alzheimer’s Disease

**DOI:** 10.1021/acs.jmedchem.3c02148

**Published:** 2024-01-08

**Authors:** Patryk Pyka, Wawrzyniec Haberek, Małgorzata Więcek, Ewa Szymanska, Wesam Ali, Agnieszka Cios, Magdalena Jastrzębska-Więsek, Grzegorz Satała, Sabina Podlewska, Silvia Di Giacomo, Antonella Di Sotto, Sabrina Garbo, Tadeusz Karcz, Chiara Lambona, Francesco Marocco, Gniewomir Latacz, Sylwia Sudoł-Tałaj, Barbara Mordyl, Monika Głuch-Lutwin, Agata Siwek, Kinga Czarnota-Łydka, Dawid Gogola, Agnieszka Olejarz-Maciej, Natalia Wilczyńska-Zawal, Ewelina Honkisz-Orzechowska, Małgorzata Starek, Monika Dąbrowska, Katarzyna Kucwaj-Brysz, Rossella Fioravanti, Muhammad Jawad Nasim, Marius Hittinger, Anna Partyka, Anna Wesołowska, Cecilia Battistelli, Clemens Zwergel, Jadwiga Handzlik

**Affiliations:** † Department of Technology and Biotechnology of Drugs, Jagiellonian University Medical College, Medyczna 9, 30-688 Kraków, Poland; ‡ Division of Bioorganic Chemistry, School of Pharmacy, Saarland University, Campus B 2.1, D-66123 Saarbrücken, Germany; $ Doctoral School of Medical and Health Sciences, Jagiellonian University Medical College, św. Łazarza 15, 31-530 Kraków, Poland; ⊥ Department of Clinical Pharmacy, Faculty of Pharmacy, Jagiellonian University Medical College, Medyczna 9, 30-688 Kraków, Poland; ∥ Department of Medicinal Chemistry, Maj Institute of Pharmacology, Polish Academy of Sciences, Smętna 12, 31-343 Kraków, Poland; # Department of Physiology and Pharmacology “V. Erspamer”, Sapienza University of Rome, Piazzale Aldo Moro 5, 00185 Rome, Italy; ¶ Italian National Institute of Health (ISS), Viale Regina Elena 299, 00161 Rome, Italy; ∇ Department of Molecular Medicine, Istituto Pasteur Italia, Fondazione Cenci-Bolognetti, Sapienza University of Rome, Viale Regina Elena 324, 00161 Rome, Italy; ⊗ Department of Drug Chemistry and Technologies, Sapienza University of Rome, Piazzale Aldo Moro 5, 00185 Rome, Italy; □ Department of Pharmacobiology, Faculty of Pharmacy, Jagiellonian University Medical College, Medyczna 9, 30-688 Kraków, Poland; △ Department of Inorganic and Analytical Chemistry, Jagiellonian University Medical College, Medyczna 9, 30-688 Kraków, Poland; ○ Department of Drug Discovery, Pharmbiotec gGmbH, Nußkopf 39, 66578 Schiffweiler, Germany; ▼ Department of Drug Delivery, Pharmbiotec gGmbH, Nußkopf 39, 66578 Schiffweiler, Germany

## Abstract

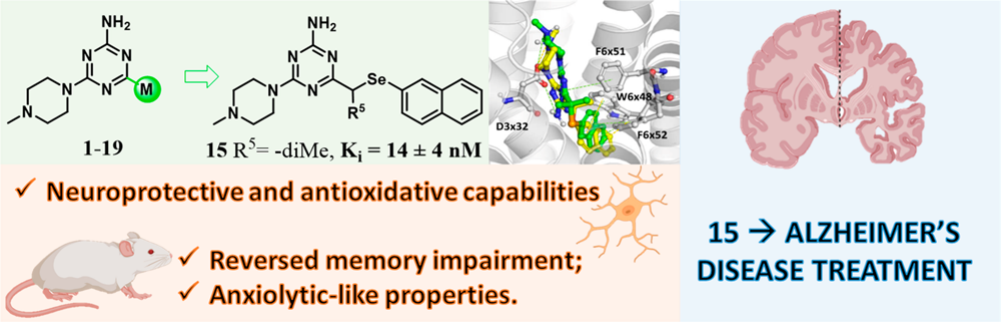

Alzheimer’s disease (AD) has a complex and not-fully-understood etiology. Recently, the serotonin receptor 5-HT_6_ emerged as a promising target for AD treatment; thus, here a new series of 5-HT_6_R ligands with a 1,3,5-triazine core and selenoether linkers was explored. Among them, the 2-naphthyl derivatives exhibited strong 5-HT_6_R affinity and selectivity over 5-HT_1A_R (**13**–**15**), 5-HT_7_R (**14** and **15**), and 5-HT_2A_R (**13**). Compound **15** displayed high selectivity for 5-HT_6_R over other central nervous system receptors and exhibited low risk of cardio-, hepato-, and nephrotoxicity and no mutagenicity, indicating its “drug-like” potential. Compound **15** also demonstrated neuroprotection against rotenone-induced neurotoxicity as well as antioxidant and glutathione peroxidase (GPx)-like activity and regulated antioxidant and pro-inflammatory genes and NRF2 nuclear translocation. In rats, **15** showed satisfying pharmacokinetics, penetrated the blood–brain barrier, reversed MK-801-induced memory impairment, and exhibited anxiolytic-like properties. **15**’s neuroprotective and procognitive-like effects, stronger than those of the approved drug donepezil, may pave the way for the use of selenotriazines to inhibit both causes and symptoms in AD therapy.

## Introduction

Alzheimer’s disease (AD) is a neurodegenerative disease affecting mainly elderly people. It is estimated that 50 million people worldwide are currently affected by this disease, and this number is estimated to triple by 2050.^1,2^ AD symptoms usually start with mild cognitive impairment and memory problems, but the neuronal damage progresses with time, leading to severe dementia, aggressiveness, and loss of even the most basic skills, such as swallowing, eventually leading to death.^1^ AD development also has a substantial impact on the mental health of patients, as depressive and anxiety disorders are commonly co-occurring.^3^ AD leads to a plethora of symptoms such as agitation, aggression, irritability, apathy, depressive mood, anxiety, psychosis, and reduced sociability, which are be summarized in the term “behavioral and psychological symptoms of dementia” (BPSD).^4^ Since the very first portrait of a dementia patient, described by Alois Alzheimer in 1907,^2^ researchers have been trying to shed light on the complex etiology of the disease, which is not yet completely understood; however, it is known that both genetic and environmental factors play roles in its development.^3^ At the neuronal level, AD is characterized by an accumulation of amyloid plaques and neurofibrillary tangles, as well as by degeneration of synapses.^5^ Despite numerous efforts to find a cure for AD, treatment options are still limited and insufficient. Antidepressants and atypical antipsychotics possess only a modest efficacy in the treatment of BPSD, with severe side effects such as cognitive slowing, cardiac arrhythmias, or daytime sleepiness, which are high-risk factors for geriatric patients;^6−9^ hence, more specific treatments are necessary.^1,4,10^ The heterogeneity of AD is probably the main obstacle to an effective and safe treatment, despite the considerable number of chemical compounds with promising preclinical activity.^11^ Only six FDA-approved drugs are currently available for AD treatment;^2^ three of them, **galantamine**, **rivastigmine**, and **donepezil**, are cholinesterase inhibitors.^12^ Indeed, decreased cholinergic neurotransmission in AD patients is linked to dementia and cognitive impairments, while increased acetylcholinesterase (AChE) levels were demonstrated to relieve the disease’s symptoms.^12^ The approved voltage-dependent, non-competitive antagonist of the *N*-methyl-d-aspartate (NMDA) receptor, **memantine**, protects neurons from excitotoxicity caused by elevated glutamate levels. Blockade of NMDA receptors with memantine is supposed to slow down disease development and is recommended for patients with moderate to severe symptoms.^12^ Unfortunately, both groups of drugs do not improve the situation of patients significantly while carrying the risk of quite severe side effects such as renal dysfunction or skin cancer.^5^ Recently, two monoclonal antibodies—**lecanemab** and **aducanumab**—have received fast-track approval from the FDA.^13,14^ Both are supposed to decompose amyloid plaques; there are some doubts about their efficacy, but it is still too early to evaluate them reliably.^15^

As outlined above, current treatment options for BPSD do not meet the clinical needs; thus, new well-tolerated target-specific medications are urgently needed.^16,17^

AD is a disease with a complex etiology, starting with a shift in neurotransmitter abundance which, in turn, triggers changes in the expression and function of certain receptors, finally resulting in neurodegeneration and aging.^18^ Nowadays, it is well accepted that treatment regimens aiming at only one pathological process are not enough; therefore, researchers focus on multitarget drugs.^19,20^ Currently, the most pursued targets are cholinesterases, metalloproteinases, monoamine oxidases, and other proteins/cascades that decrease neuroinflammation, β-amyloid deposits, or oxidative stress.^21^ In the context of these targets, often a massive accumulation of reactive oxygen species (ROS) has been observed to cause neuroinflammation and to result in cytotoxic oxidation, such as lipid peroxidation or DNA damage, synergistically participating in the onset and progression of AD and giving rise to neurons’ death.^22^

In this frame, a central role for the nuclear factor erythroid 2-related factor 2 (NRF2) has been unveiled.^23,24^ This protein is an important regulator of cellular antioxidant response, and upon the increase of ROS production, it translocates into the nucleus, where it induces the transcription of genes involved in the antioxidant response. Specifically, it induces the expression of heme oxygenase-1 (HO-1), quinone oxidoreductase-1 (NQO-1), and superoxide dismutase (SOD),^25,26^ but it also reduces the activity and expression of beta-site amyloid precursor protein cleaving enzyme 1 (BACE1), increasing the production of amyloid-β (Aβ).^27^ Thus, NRF2 can be considered as a promising target to be activated to mitigate AD progression.

In recent years, the number of studies exploring novel, innovative targets has rapidly increased.^28^ An interesting representative of such a more and more exploited target is the serotonin receptor 5-HT_6_ (5-HT_6_R), found almost exclusively in the central nervous system (CNS); thus, targeting this receptor should carry a low risk of peripheral adverse effects.^29^ 5-HT_6_R was discovered in 1993 as one of the last members of the serotoninergic system^30,31^ and has been associated with diverse CNS dysfunctions (e.g., depression and schizophrenia).^32−34^ Physiologically, 5-HT_6_R modulates numerous neurotransmitter pathways, and the blockade of this receptor leads to increased cholinergic and glutaminergic neurotransmission;^29^ furthermore, it also facilitates the release of dopamine and norepinephrine in the frontal cortex.^35^ Recent preclinical studies have shown that 5-HT_6_R antagonists and agonists are capable of improving memory impairment in novel object recognition (NOR), social recognition (SRT), Y-maze continuous spontaneous alternation (Y-CAT), and Morris water maze (MWM) tests in rats,^36−38^ underlining the importance of this target for AD, while also exhibiting anxiolytic and antidepressant effects.^32,39,40^ Interestingly, both 5-HT_6_R agonists and antagonists paradoxically exhibited procognitive, antidepressant, and antianxiety properties.^41^ The selective agonists **WAY-181187**, **WAY-208466**, and **E-6801**,^42−44^ the partial agonist **EMDT 386088**, and the antagonists **SB-271046** and **SB-399885** have been extensively studied in preclinical and clinical settings without reaching approval.^32,45,46^

**Figure 1:**
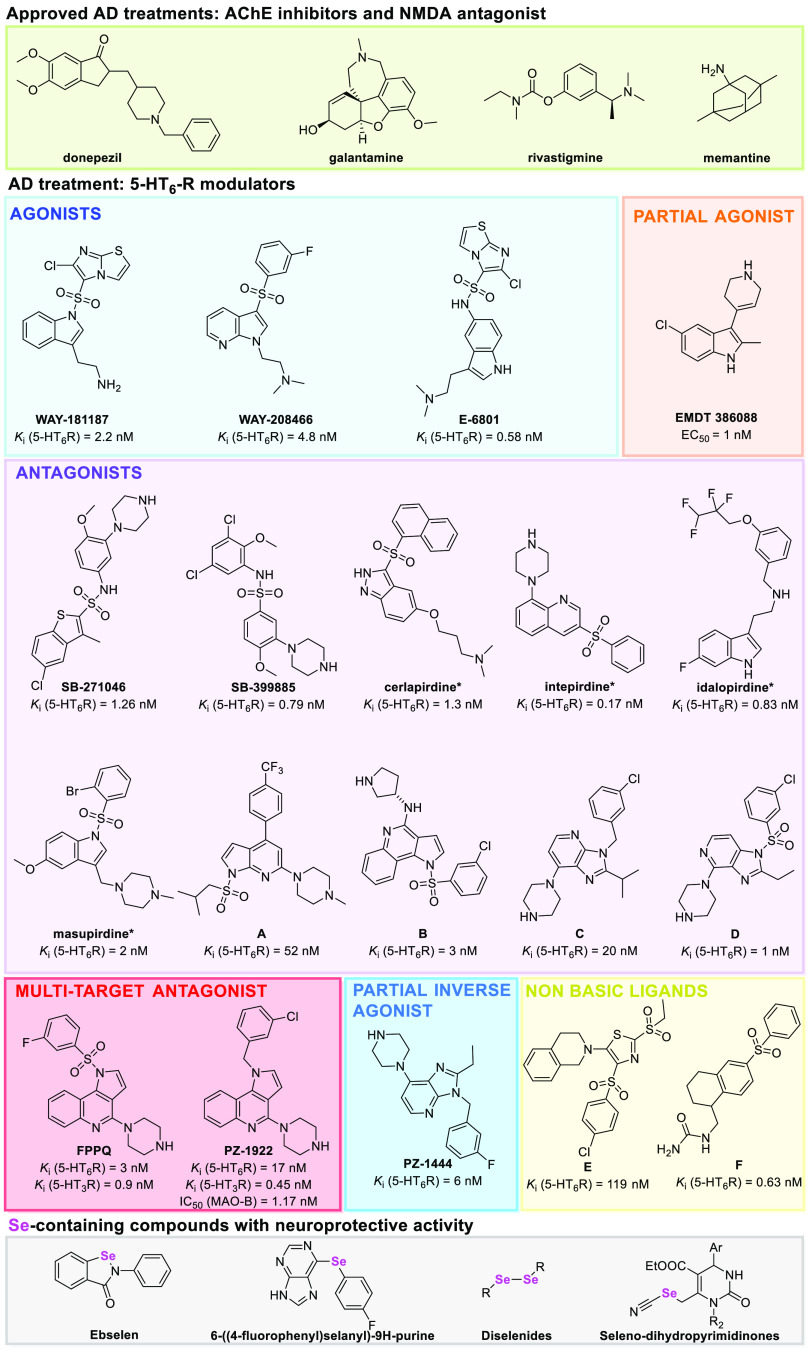
Approved AD drugs and 5-HT_6_R ligands in preclinical and *clinical trials and Se-containing compounds with neuroprotective activity.^12,32,45−50,53−56,58,60,62−70^

More recently, other 5-HT_6_R antagonists such as **A**, **B**, **C**, and **D** (Figure 1)^47−50^ have been shown to possess a nanomolar affinity to the target; however, they display a high mutual similarity due to the presence of an indole-like core and/or sulfonyl groups; thus, the chemical space in the search for novel 5-HT_6_R agents needs to be broadened to achieve a potent and selective action on 5-HT_6_R with satisfying CNS druggability.^51^

**Cerlapirdine** (**SAM-531**) showed promising results in Phase I studies in healthy subjects (NCT00479349, NCT00479700). However, in Phase II studies in mild to moderate AD patients (NCT00895895), all three dosage levels (1.5, 3, and 5 mg) were found to be unsatisfactory, and the study was terminated. Moreover, **HEC30654** is a 5-HT_6_ receptor antagonist with good preclinical results in cognition tests, and recently its safety, tolerability, and pharmacokinetic (PK) profile have been evaluated in healthy Chinese subjects with encouraging data.^52^

5-HT_6_R antagonists, in combination with cholinesterase inhibitors, were tested in clinical trials as a potential therapy for AD. Two agents, **idalopirdine** and **intepirdine**, have reached Phase III of clinical trials yet failed to demonstrate statistically significant improvement in cognition,^53−55^ probably due to the complexity of this disorder that may require a multitarget approach rather than a single selective one.^11^ However, it is still important to design novel molecules in such a way that their selectivity profile will remain directed toward 5-HT_6_R over homologous serotonin receptors 5-HT_2A_, 5-HT_1A_, and 5-HT_7_, as a differentiated modulation has a significant influence on the observed therapeutic effects.^11^ For example, **masupirdine** (**SUVN-502**) is highly selective for 5-HT_6_R and has minimal activity on the 5-HT_2A_ receptor, in contrast with **idalopirdine** and **intepiridine**. **Masupirdine** was developed by Nirogi et al.^56^ and demonstrated positive procognitive effects in various behavioral tests in animal models. It also modulated glutamate levels and potentiated the effects of donepezil and memantine. The beneficial effects of masupirdine on learning and memory may be mediated by the modulation of cholinergic and/or glutamatergic neurotransmission in relevant brain regions. Indeed, the results of a Phase II study (NCT02580305) involving masupirdine in combination with memantine and donepezil for the treatment of moderate AD highlights that administration of masupirdine improves cognitive functions and reduces agitation/aggression scores.^57,58^

Particularly, by merging the structure of **C** with a pyrroloquinoxaline ligand of 5-HT_3_,^59^ Zajdel et al. obtained **FPPQ**, a dual-acting 5-HT_3_/5-HT_6_ antagonist that alleviates symptoms in psychiatric disorders and has procognitive properties.^60^ Staying within the realm of multitarget antagonists, Grychowska et al.—employing *in silico* analysis and cryo-electron microscopy techniques—designed, synthesized, and evaluated **PZ-1922**, an innovative triple-acting compound, as a potential therapy for AD.^61^ It demonstrated notable antagonistic activity at both 5HT_6_R and 5-HT_3_R alongside a robust, reversible inhibition of MAO-B. Furthermore, PZ-1922 exhibited favorable PK properties, and the findings of this study unequivocally showcased the superiority of PZ-1922 over intepirdine in terms of its capacity to prevent and mitigate molecular and synaptic alterations while also effectively modulating neuroinflammatory processes in the hippocampus of rats subjected to Aβ injections.

Vanda and colleagues developed **PZ-1444**, a 5-HT_6_ receptor partial agonist with nanomolar IC_50_ and *K*_i_ values.^49^ This compound showed good PK properties and had procognitive properties, reversing phencyclidine- and scopolamine-induced memory deficit.

All 5-HT_6_R ligands tested *in vivo* had a positive ionizable center responsible for the key interaction with serotonin receptors (salt bridge with Asp3.32). Some attempts to develop atypical non-basic 5-HT_6_R ligands have been reported *in vitro*, but their design remains highly challenging. Examples of this approach are molecules **E** and **F**.^62,63^

**Figure 2:**
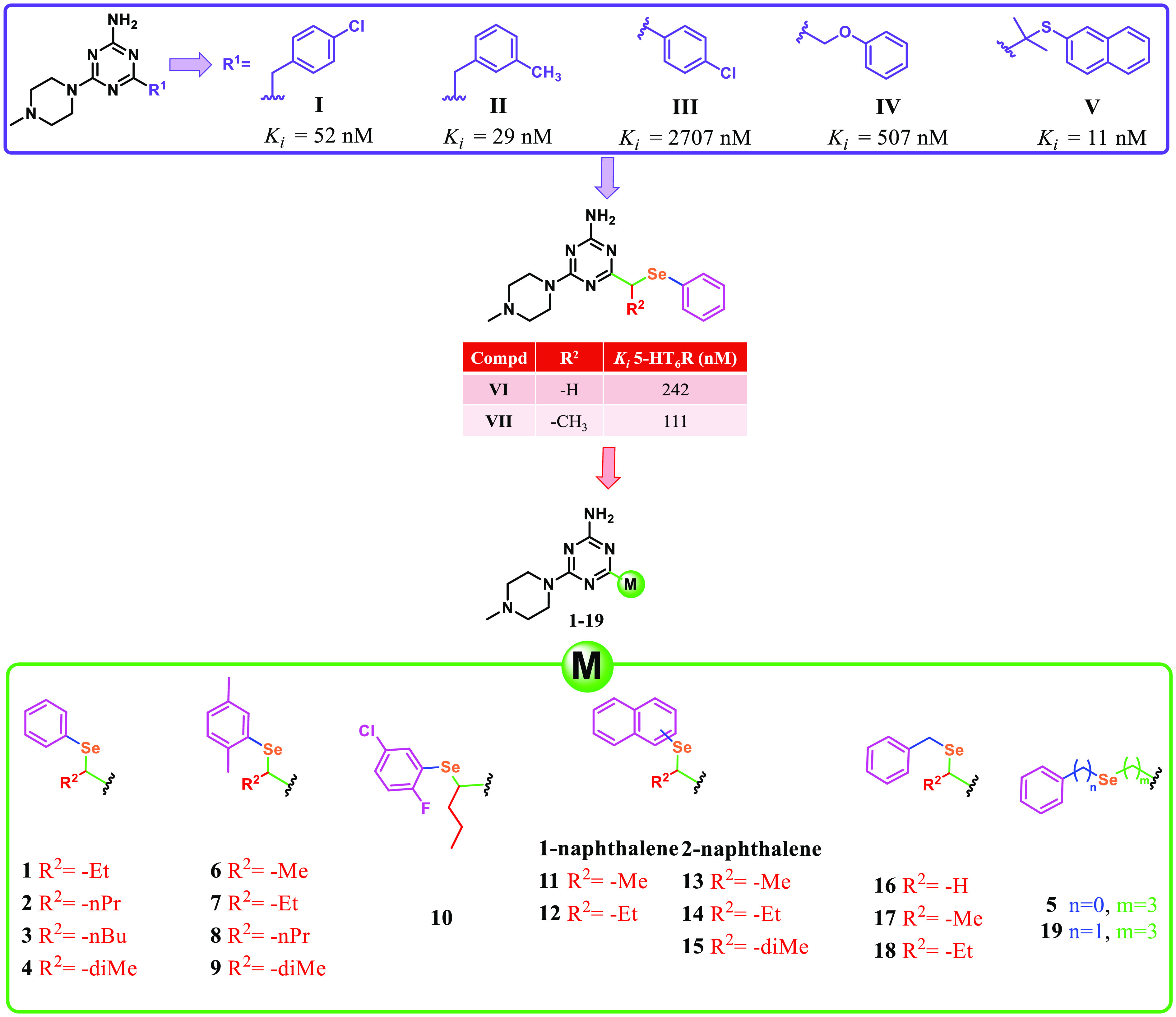
Selenoether 5-HT_6_R ligands described by our research team, **I–VII**, and new potential 5-HT_6_R ligands, **1**–**19**.^40,51,71−73^

In recent years, our research group developed a new family of potent and selective 5-HT_6_R ligands with a 1,3,5-triazine core (**I–V**, Figure 2), which, unlike other known ligands, possess neither an indole moiety nor a sulfonyl group in their structures.^40,51,71−73^ In 2019, Ali et al. explored applications of different chalcogen linkers in search of 5-HT_6_R agents among 1,3,5-triazine derivatives.^51^ Particularly, attention should be paid to compounds **VI** and **VII** (Figure 2) containing selenoether linkers. Both show moderate activity toward 5-HT_6_R with potential for optimization while being twice as active as the corresponding oxygen analogs, underlining the increasing importance of seleno compounds in the search for new therapies for CNS diseases.^74^

Indeed, selenium plays various roles in the progression of some neurodegenerative diseases such as AD.^75,76^ It is crucial for the activity of glutathione peroxidase (GPx), which is responsible for protecting organisms from oxidative damage; studies show that supplementation with this element is beneficial for patients with AD and mild cognitive impairment.^77^ The toxicity of organoselenium compounds strongly depends on the selenium oxidation state and types of substituents.^78^ The most dominant form of selenium in organic compounds is Se(II), and in this form, compounds present relatively low toxicity,^78,79^ although in very high doses they may generate ROS and cause thiol depletion. However, more studies are reporting their antioxidant and neuroprotective action^79^ as well as their potential use in the treatment of various pathological conditions spanning cancer and non-cancer disorders.^64^ Selenium-containing molecules are primarily recognized for their physiological role as antioxidants, particularly through a group of selenoproteins that employ selenocysteine (Sec) residues in their enzyme active sites to catalyze redox reactions, safeguarding organisms from oxidative stress.^80^ In particular, selenium-containing compounds are typically classified into three main categories: inorganic selenium compounds, organic selenium compounds, and selenoproteins.^81^ Typically, the PK properties of selenium compounds are favorable. Selenium compounds exhibit increased uptake, particularly by cancer cells, although the precise mechanism of selective selenium uptake in cancer cells remains incompletely understood.^81^ So far, various mechanisms have been described. For example, selenide may be transported by ATPases,^82^ while selenite uptake can be mediated by anion transporters.^83^ The metabolic stability and excretion routes of Se-containing compounds vary considerably.^80,84,85^

Organoselenium compounds exhibit reduced toxicity and enhanced bioactivity when compared to their inorganic selenium counterparts^64^ and are able to act as mimetics of GPx7 that modulates oxidative stress in the brain.^86^ Several studies report neuroprotective and antioxidant properties of diphenyl diselenide and **ebselen**.^64,87−89^ The neuroprotective effects of ebselen are related to its antioxidant properties. The compound reduced malondialdehyde overproduction and boosted SOD activity in brain tissue during ischemia/reperfusion (I/R).^65^ Ebselen demonstrated protection against amyloid neurotoxicity and improved cognitive function in AD by reducing Aβ levels and inhibiting tau protein hyperphosphorylation.^66,67^ The organic selenide 6-((4-fluorophenyl)selanyl)-9*H*-purine showed the ability to inhibit AChE in the brain and enhance memory in a mouse model, underscoring its potential as a treatment option for AD.^90^ Some aromatic diselenides tested in rodents have been confirmed to improve cognitive performance without inducing neurotoxic effects.^68^ In particular, *p*-methoxyphenyl diselenide improved mice’s memory, protected them against Aβ-induced neurotoxicity, and inhibited AChE activity in a model of sporadic Alzheimer’s-type dementia.^69^ Similar or even better effects were observed for selenodihydropyrimidinones, primarily acting as potent AChE inhibitors, which additionally showed a very high antioxidant activity through different mechanisms of action^64,70,80^ (Figure 1).

In general, selenium-containing compounds demonstrate higher biological activity than their sulfur-containing counterparts. This enhanced activity is likely attributed to subtle chemical distinctions between these two elements.^80,91^ Due to the larger atomic radius of Se than other chalcogens, more loosely bound outer valence electrons occur, resulting in enhanced antioxidant properties^91^ with stronger electron acceptor and more electrophilic properties than oxygen or sulfur analogs. Se’s higher polarizability generally increases the lipophilicity and permeability of drug molecules and makes it a stronger nucleophile, facilitating its coordination with metal centers in enzyme catalytic sites.^92^

Considering all the benefits mentioned above, a new series of selenoether derivatives of 1,3,5-triazine has been designed, synthesized, and evaluated *in vitro* for 5-HT_6_R affinity and neuroprotective action and *in vivo* in animal models of CNS diseases.

## Results and Discussion

### Chemistry

**Scheme 1:**
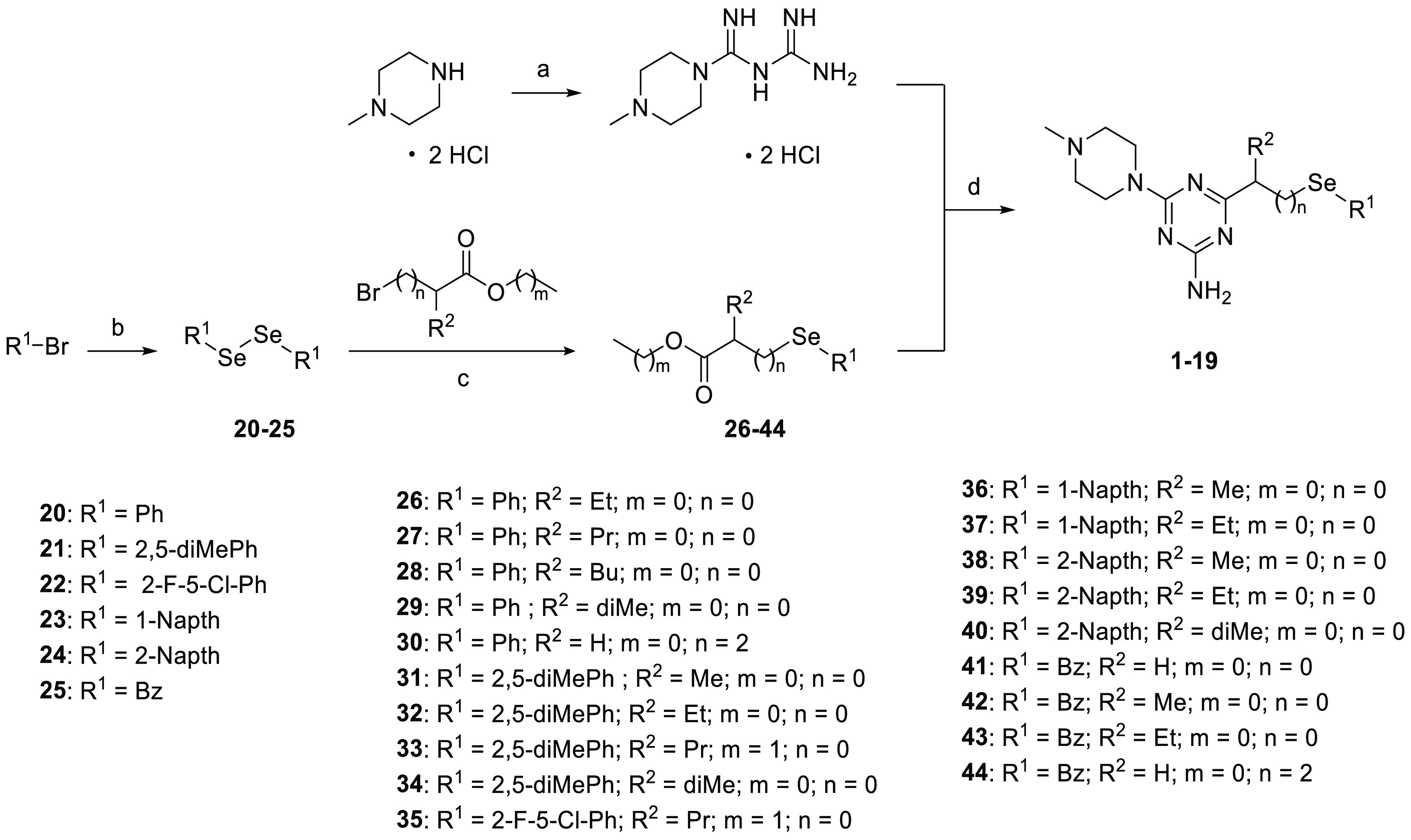
Synthesis of Final 1,3,5-Triazine Products Reagents and conditions: (a) 1-cyanoguanidine, BuOH, 125 °C, 24 h; (b) Se, Mg, I_2_, anhydrous THF, rt, 24 h; (c) NaBH_4_, THF:H_2_O 1:1, rt, 24–48 h; (d) Na, CH_3_OH, reflux, 15–30 h.

The final compounds described in this study (**1**–**19**) were synthesized through a 3–4-step synthesis pathway. The syntheses of **1**, **2**, **4**, **5**, **16**, **18**, and **19** were described previously.^51,93,94^ The synthesis of (4-methyl-1-piperazinyl)biguanide dihydrochloride (Scheme 1) was performed using commercially available 4-methylpiperazine dihydrochloride and 1-cyanoguanidine, following a previously described method.^95^ Diphenyl diselenide and dibenzyl diselenide are the only aryl diselenides commercially available, and the other desired aryl diselenides **20**–**25** were obtained via the formation of the Grignard reagent by treating commercial aryl halides with magnesium under inert conditions (Scheme 1). The resulting selenium Grignard reagent easily oxidizes in the presence of air, leading to a selenium products mixture in which the diselenides are the predominant species. However, the byproducts in the mixture were found to be inert to the subsequent reactions; therefore, the obtained diselenides **20**–**25** were used as crude products at the next step.

The cleavage of diselenides with sodium borohydride was performed under an argon atmosphere and led to the *in situ* formation of an arylselenide anion, which reacted with an appropriate bromoester. The syntheses of the 1,3,5-triazine selenium derivatives **1**–**19** involved the preparation of sodium methanolate, to which a suitable aryl selenium ester (**26**–**44**) and (4-methyl-1-piperazinyl)biguanide dihydrochloride were added. Condensation reactions were carried out according to a method described previously,^40,51^ utilizing purification on flash chromatography, crystallization from water, or transformation into the corresponding crystalline hydrochloric salts. Spectral (^1^H, ^13^C, ^77^Se NMR) and chromatographic (LC/MS) analytical methods confirmed the structures and purity (>95%) of the final compounds.

### Pharmacology

#### The Action on 5-HT_6_R and 5-HTRs Off-Targets:

##### Radioligand Binding and Functional Assays

The whole series of **VI**, **VII**, and **1–19** was investigated to determine their affinities for serotonin receptors, including the main target 5-HT_6_R and the off-targets 5-HT_1A_R, 5-HT_2A_R, and 5-HT_7_R, in the radioligand binding assay (RBA) using the methods described previously.^51^ Olanzapine, buspirone, aripiprazole, and clozapine were used as highly potent reference ligands toward the 6, 1A, 2A, and 7 serotonin receptor subtypes, respectively (Table 1). Moreover, a selected chemical subgroup of the most active 5-HT_6_R agents (i.e., the naphthyl derivatives) was examined to determine their intrinsic action in functional studies using the cAMP measurement assay according to procedures described before^60,96^ (details in Figure S4 and Table S3).

**Table 1:** Affinities (*K*_i_) for Compounds **VI**, **VII**, **1**–**19** to Serotonin Receptors Assessed in Radioligand Binding Assays, Selectivity Index (SI), and Antagonistic Action (*K*_b_) in Functional Assays with cAMP

	***K***_**i**_**± SD (nM)**				**SI**			***K***_**b**_**(nM)**
**Compd**	**5-HT**_**6**_	**5-HT**_**1A**_	**5-HT**_**2A**_	**5-HT**_**7**_	**5-HT**_**1A**_**/5-HT**_**6**_	**5-HT**_**2A**_**/5-HT**_**6**_	**5-HT**_**7**_**/5-HT**_**6**_	**5-HT**_**6**_**R**
**VI**	*242 ± 10*	6647 ± 1498	329 ± 57	2521 ± 536	15.107	0.748	5.730	ND
**VII**	*111 ± 9*	5311 ± 1256	376 ± 41	4247 ± 983	47.847	3.387	38.261	ND
**1**	*122 ± 13*	4084 ± 764	1011 ± 198	4393 ± 721	33.475	8.287	36.008	ND
**2**	*52 ± 10*	3702 ± 831	623 ± 72	3161 ± 752	71.192	11.981	60.788	ND
**3**	*33 ± 4*	3812 ± 829	336 ± 68	4177 ± 864	115.515	10.182	126.576	ND
**4**	*165 ± 31*	2900 ± 613	793 ± 154	5244 ± 1167	17.576	4.806	31.782	ND
**5**	*193 ± 21*	1718 ± 266	414 ± 39	7376 ± 1837	8.902	2.145	38.218	ND
**6**	*46 ± 8*	5110 ± 1027	899 ± 183	4745 ± 829	111.087	19.543	103.152	ND
**7**	*22 ± 6*	4145 ± 921	579 ± 106	4186 ± 467	188.409	26.318	190.273	ND
**8**	*80 ± 15*	4645 ± 1085	517 ± 127	5667 ± 1356	58.063	6.463	70.838	ND
**9**	*75 ± 11*	3671 ± 406	534 ± 98	2546 ± 625	48.947	7.120	33.947	ND
**10**	*21 ± 5*	2070 ± 354	306 ± 86	4006 ± 719	98.571	14.571	190.762	ND
**11**	*21 ± 3*	6071 ± 1352	301 ± 51	8962 ± 1958	289.095	14.333	426.762	33.1
**12**	*36 ± 7*	5948 ± 1469	198 ± 29	7874 ± 1594	165.222	5.500	218.722	26.14
**13**	*9 ± 3*	2999 ± 520	130 ± 18	2295 ± 429	333.222	14.444	255.000	9.99
**14**	*8 ± 2*	2306 ± 504	18 ± 3	5109 ± 981	288.250	2.250	638.625	7.87
**15**	*14 ± 4*	3533 ± 437	35 ± 5	1449 ± 173	252.357	2.500	103.500	15.0
**16**	*278 ± 49*	7614 ± 1822	1018 ± 197	4450 ± 1002	27.388	3.662	16.007	ND
**17**	*1023 ± 216*	1904 ± 253	1722 ± 359	6993 ± 1464	1.861	1.683	6.836	ND
**18**	*3065 ± 687*	1149 ± 181	1022 ± 176	8752 ± 1898	0.375	0.333	2.855	ND
**19**	*79 ± 9*	893 ± 117	834 ± 205	7450 ± 1725	11.304	10.557	94.304	ND
**Ref ligand**	*7^a^*	32^b^	21^c^	62^d^	–	–	–	2.38^e^

a Olanzapine.

b Buspirone.

c Aripiprazole.

d Clozapine.

e SB258585. 5-HT_6_R affinities are shown in italics. ND, not determined.

As shown in Table 1, most of the tested compounds (**VI**, **VII**, **1**–**16**, and **19**) displayed significant submicromolar (*K*_*i*_ < 500 nM) affinities for 5-HT_6_R and distinct selectivity over 5-HT_1A_R and 5-HT_7_R off-targets. Most of the compounds (**VII**, **1**–**16**, **19**) were also at least slightly selective toward 5-HT_6_R with respect to 5-HT_2A_ receptors, but the affinity for that off-target was in the submicromolar range for a majority of the compounds (**VI**, **VII**, **2**–**15**, **19**).

In more detail, among the series of phenyl derivatives **1**–**3**, it is apparent that an increase in the number of carbons within the side chain correlates with an increased affinity for 5-HT_6_R (*K*_i_ = 122, 52, and 33 nM for **1**, **2**, and **3**, respectively) and, concurrently, an augmented selectivity index (SI) toward 5-HT_6_/5-HT_2A_ but especially toward 5-HT_1A_R and 5-HT_7_R, where the increase of a carbon atom within the side chain correlates with a 2-fold increase in selectivity. Conversely, the introduction of a nonlinear entity, such as a dimethyl residue as in compound **4**, exhibits a decline in both affinity and the associated 5-HT_6_R SI. A longer chain augmenting the distance between the selenoether and the triazine core, as seen for derivative **5**, resulted in a drop of activity and selectivity for 5-HT_6_R, with *K*_i_ = 193 nM and SI < 10 toward both 5-HT_1A_R and 5-HT_2A_R. Looking at the derivatives featuring a 2,5-dimethylphenyl moiety, **6**–**9** uniformly exhibit favorable affinity values as well as SIs. Notably, by comparing derivatives possessing identical R^2^ side chains (Figure 2), it is evident that derivative **7** surpasses analog **1** (R^2^ = Et) in affinity and selectivity. This discernible pattern is most effectively accentuated by comparing derivatives **4** and **9**, which share the same side chain (R^2^ = diMe). In this context, **9** demonstrated superior affinity and selectivity compared to **4**. Compound **10** and its halogenated analog **3** demonstrated comparable affinity and selectivity values. Replacing the phenyl with the naphthyl moiety resulted in the most promising compounds in our series. In more detail, regarding the 1-naphthyl compounds, **11**, with the small methyl side chain, possesses an almost 2-fold superior 5-HT_6_R affinity (*K*_i_ = 21 nM) and selectivity over 5-HT_1A_R, 5-HT_2A_R, and 5-HT_7_R when compared to its ethyl counterpart, **12** (*K*_i_ = 36 nM). For derivatives harboring a 2-naphthyl group, **13**–**15**, the affinity for 5-HT_6_R remains nearly uniform. However, concerning selectivity, the preeminent derivative emerges to be **13** bearing R^2^ = Me. Upon considering the other derivatives, for **6** and **11**, featuring a methyl lateral chain (R^2^ = Me), a distinct structure–activity relationship (SAR) is observed, with affinity successively ascending twice from 2,5-dimethylphenyl (**6**, *K*_i_ = 46 nM) to 1-naphthyl (**11**, *K*_i_ = 21 nM) and culminating in 2-naphthyl (**13**, *K*_i_ = 9 nM), accompanied by corresponding elevations in 5-HT_6_R selectivity of 3-fold with respect to 5-HT_1A_R (**6**–**13**) and of 4-fold with respect to 5-HT_7_R (**6**–**11**). The exceptions are for the 5-HT_6_R/5-HT_7_R selectivity, which regresses almost 2-fold from the 1-naphthyl derivative **11** (SI > 400) to the 2-naphthyl derivative **13** (SI > 250), and for the 5-HT_6_R/5-HT_2A_R selectivity, which decreases about 1.3-fold from the 2,5-dimethylphenyl **6** to both the 1- and 2-naphthyl compounds **11** and **13**. For derivatives **1**, **7**, **12**, and **14** characterized by R^2^ = Et, a trend is discernible analogous to those reported for derivatives possessing R^2^ = Me. Of particular significance, **14** emerges as the most efficacious compound regarding 5-HT_6_R affinity, with *K*_i_ = 8 nM. By evaluating the selectivities of these ethyl derivatives, it emerges that the augmentation in binding affinity encountered during the transition from monoaryl (*K*_i_ = 122 and 22 nM for **1** and **7**, respectively) to naphthyl derivatives (*K*_i_ = 36 and 8 nM for **12** and **14**, respectively) is accompanied by an enhanced selectivity for 5-HT_6_R with respect to 5-HT_1A_R and 5-HT_7_R receptors, while at the same time selectivity between 5-HT_6_R/5-HT_2A_R receptors is diminished. Indeed, by comparing the phenyl derivative **1** with the naphthyl compound **14**, it emerges that the 5-HT_6_R SI passes from 33 to 288 for 5-HT_1A_R and from 36 to 638 for 5-HT_7_R_,_ but for the 5-HT_2A_R the SI decreases from 8 to 2. For derivatives encompassing a dimethyl substituent on R^2^ (**4**, **9**, **15**), an analogous overarching pattern is evident, characterized by increased binding affinity (*K*_i_ = 165, 75, and 14 nM for **4**, **9**, and **15**, respectively) and selectivity toward 5-HT_6_ and 5-HT_7_ receptors, albeit with a corresponding attenuation of selectivity with respect to 5-HT_2A_ receptors. By comparing the phenyl derivative **4** with the naphthyl compound **15**, it can be noticed that the 5-HT_6_R SI passes from 17 to 252 for 5-HT_1A_R and from 31 to 103 for 5-HT_7_R, but for 5-HT_2A_R the SI decreases from 4 to 2.5. In contrast, all benzyl derivatives **16**–**18** exhibit a more than 50-fold loss in affinity toward the 5-HT_6_ receptor with respect to the phenyl derivatives. Notably, derivative **19**, featuring a benzyl entity separated from the 1,3,5-triazine scaffold by three carbons, experiences a partial restoration in bond affinity, albeit displaying only a mediocre selectivity. Intriguingly, this is particularly surprising as the selenophenyl ether **5**, maintaining the three-carbon linker of **19** as spacer from the 1,3,5-triazine scaffold, results in drops of about 2-fold in both affinity and selectivity for 5-HT_6_R. The radioligand-based data indicated the naphthyl derivatives **11**–**15**—the most potent chemical subgroup of the series at 5-HT_6_R examined—in the low double-digit or even single-digit nanomolar range; thus, they were selected for the intrinsic activity assays (Table 1, Figure S4, and Table S3). The data indicate that **11**–**15** are potent antagonists of 5-HT_6_R (*K*_b_ in the range of 7.87–3.1 nM), well correlating with their affinities toward 5-HT_6_R measured in RBA. Overall, these results confirmed the general tendency observed for our previous other 5-HT_6_R triazine ligands, with a majority also presenting an antagonistic mode of action,^51,71−73,97^ with the exception of only a few halogen-substituted phenyl-thioether derivatives.^96^

##### 5-HT_6_R Docking Analysis

**Figure 3:**
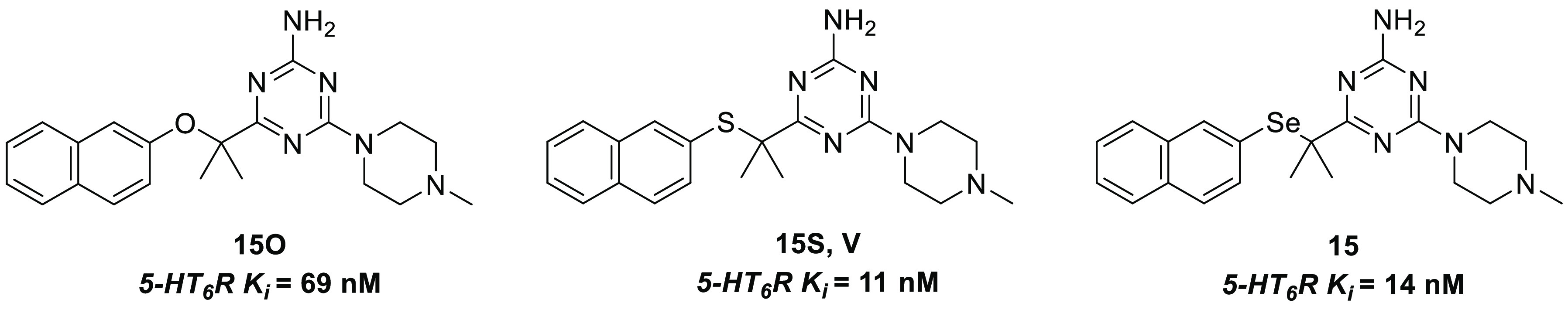
Structures of the oxygen (**15O**) and sulfur (**15S**, **V**)^11^ analogs of **15**. The synthesis and characterization of **15O** are shown in the Supporting Information.

In order to better explain the trends of the interactions with 5-HT_6_R for the investigated series (**VI**, **VII**, **1**–**19**) at a molecular level, docking studies have been performed. Due to the antagonistic mode of action of the modeled compounds, the GPCRdb homology model of 5-HT_6_R^98,99^ was used, which captures the inactive conformation of the receptor. At first, **4**, **9**, and **15**, possessing a dimethyl group in the branching linker and different types of aromatic moieties (phenyl, 2,5-dimethylphenyl, and β-naphthyl, respectively), were considered, along with the oxygen (**15O**) and sulfur (**15S**, shown as **V** in Figure 2) analogs of **15** (Figure 3).^11^ It can be observed that, within this set of compounds, the sulfur- and selenium-containing compounds are more active than the oxygen-containing analog. In addition, modifications of the aromatic moiety led to changes in 5-HT_6_R activity in the following order: phenyl < 2,5-dimethylphenyl < β-naphthyl (*K*_i_ = 165 nM vs 75 nM vs 14 nM, respectively).

**Figure 4:**
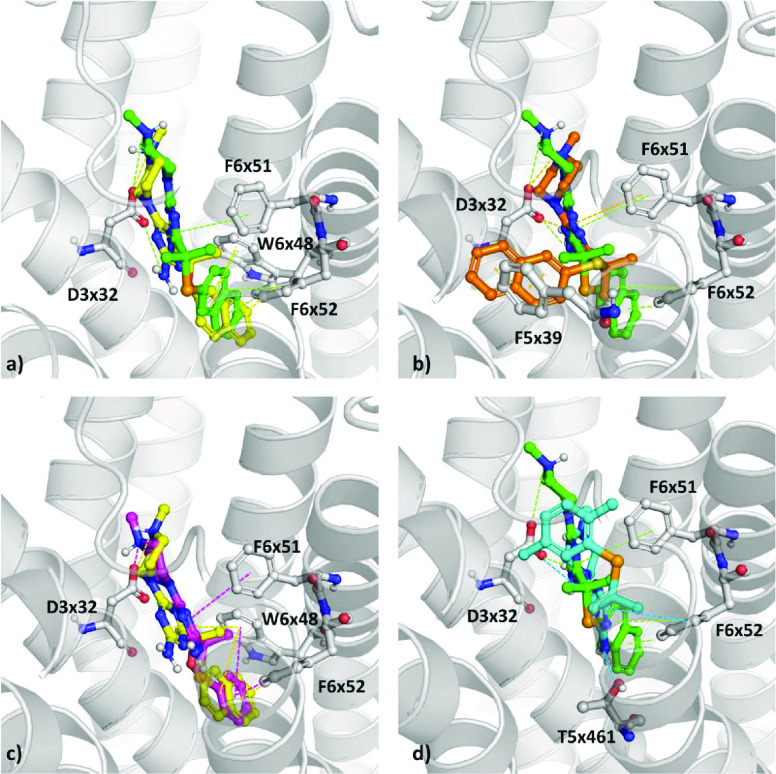
Docking results of the selected compounds to 5-HT_6_R homology model: (a) **15**, green; **15O**, yellow. (b) **15**, green; **15S**, orange. (c) **15O**, yellow; **4**, magenta. (d) **15**, green; **9**, cyan. Models used are from refs (98 and 99).

All analyzed compounds (**4**, **9**, **15**, **15O**, and **15S**) fit well in the 5-HT_6_R binding pocket and formed a charge-assisted hydrogen bond with aspartic acid from the third transmembrane helix (D3x32 according to the GPCRdb numbering, Asp106), which is reported in many studies as essential for the 5-HT_6_R activity.^48,100−102^ In all studied cases, the triazine ring is located in the proximity of the phenylalanine cluster (F6x51 (Phe284) and F6x52 (Phe285)), resulting in the formation of the π–π contacts (Figure 4).

More detailed analysis of compounds containing different chalcogens (**15**, **15O**, **15S**) revealed that **15** and **15O** adopted similar binding poses (Figure 4a), with naphthyl moieties oriented toward the inner part of the pocket and π–π interactions with W6x48 (Trp281) and F6x52. The main difference in the orientations of **15** and **15O** is related to the position of the triazine ring and its substituents. For **15**, the amine group attached to triazine faces toward D3x32, while for **15O**, the amine group is oriented in the opposite direction. As the amine group in **15** acts as an H-bond donor and takes part in the formation of the additional hydrogen bond with D3x32, this variation in the position of the amine group might be crucial for the activity of **15**. On the other hand, the binding pose of **15S** is different (Figure 4b), and the naphthyl moiety is oriented toward the outer side of the pocket, with its position being stabilized by π–π interactions with F5x39 (Phe188). Nevertheless, the positions of the triazine ring and piperazine are almost identical for **15** and **15S** (triazine rings in both cases form π–π contacts with F6x51), and amine groups attached to this moiety form hydrogen bonds with D3x32. Having in mind that the 5-HT_6_R activities of **15** and **15S** are similar (*K*_i_ = 14 nM and 11 nM, respectively), while the activity of **15O** is approximately 6 times lower (*K*_i_ = 69 nM), it seems that the formation of hydrogen bonds by the amine group of triazine ring is more important for 5-HT_6_R activity than the orientation of the aromatic moiety.

The differences in the binding orientation observed for the three analogs **15O**, **15S**, and **15** are intriguing due to their high structural similarity. Thus, we delved deeper into this issue by analyzing their mutual overlap and detailed interactions within the binding pocket (Figures S1 and S2). Although **15** and **15O** look similar at first glance, detailed examination demonstrates that the amino groups on triazine are directed in two different directions, which indicates that the compounds are inverted (about 180° in relation to the other). In **15** and **15S**, the triazine and piperazine positions are much closer, and the difference starts from the carbon, to which a chalcogen and two methyl groups are attached. Superimposition of **15O** and **15S** indicates poses rotated in an even different way than in the previous two cases. The differences in active conformations shown in the docking studies have their source in differences in the sizes and chemical properties of the linker heteroatoms (O, S, Se), where both the radius of the heteroatom and the dipole moment of the molecule increase in the order O < S < Se. This differentiation of properties (O vs S vs Se) gives rise to a variety of intramolecular interactions, the type (e.g., chalcogen bonds possible for S and Se but not for O) and the strength of which are crucial for the resulting conformation of the given molecule (**15**, **15S**, **15O**), subsequently conditioning its entry and positioning in the binding pocket.

In order to confirm the obtained docking poses and their representativeness for the compound orientation in the binding site, molecular dynamics (MD) simulations were carried out for **15** and **15S**, using Desmond (length of each simulation: 250 ns); analysis of changes in the ligand–protein contacts occurring during MD simulations is visualized in Figure S1. Figures S2 and S3 analyze the presence of interactions with particular amino acids at a given time. The consistency in the occurrence of particular contacts during the simulation confirms the validity of the obtained docking poses.

On the other hand, the influence of the aromatic moiety on compound affinity toward 5-HT_6_R was studied with compounds **4**, **9**, and **15**. Compound **4** (Figure 4c) displayed a pose analogous to that of **15O**, with an aromatic ring oriented toward the inner part of the protein and lack of a hydrogen bond between the amine group attached to the triazine ring and D3x32. The weaker 5-HT_6_R affinity of **4** (*K*_i_ = 165 nM vs 69 nM for **15O**) might also be conditioned by its less effective stabilization by π–π interactions due to the presence of only one aromatic ring instead of two, as in the case of naphthyl derivatives. Compound **9** adopted a different binding mode (Figure 4d), with the triazine ring stabilized by π–π contacts with F6x51 and F6x52 and the amine group forming a hydrogen bond with T5x461 (Thr196). The aromatic ring of the 2,5-dimethylphenyl group is oriented outside of the binding pocket, yet without the formation of the π–π contact with F5x39.

Summing up, the main components of the compounds presented in the study are as follows: a positive ionizable group (present in the form of the protonated tertiary amine in the piperazine moiety) forms the charge-assisted hydrogen bond with D3x32, and an aromatic triazine ring stabilizes compounds in the binding pocket via π–π contacts with phenylalanines from the sixth and fifth transmembrane helices (TM6 and TM5). In addition, for the most potent compounds, the amine group substituting the triazine ring acts as a hydrogen bond donor for the charge-assisted contact with D3x32. Finally, a big aromatic moiety, oriented either toward the inner part of the pocket, and thus being part of the π–π contact with W6x48, or to the outer side, making an analogous interaction with F5x39, also plays a huge role in the provision of high 5-HT_6_R activity, with the increasing activity being related to the increasing aromatic surface.

Both experimental and computational studies indicated the most favorable 5-HT_6_R action profile for the 2-naphthyl derivatives, **13**–**15**. These compounds demonstrated particularly high affinity (*K*_i_ < 15 nM), potent antagonistic activity (*K*_b_ < 15 nM), significant selectivity over 5-HT_1A_R and 5-HT_7_R, and potent action on 5-HT_2A_R (*K*_i_ < 50 nM) in the case of **14** and **15**, while almost 15-fold 5-HT_6_R/5-HT_2A_R selectivity was noticed for **13**.

Thus, the three compounds **13**–**15** were selected for the subsequent extended biological assays.

### Neuroprotection

#### Neurotoxicity and Neuroprotection in Neuroblastoma SH-SY5Y

The potential toxicity of novel compounds in the search for therapeutics should always be considered. Neurotoxicity is specific in this respect, as the normal function of the nervous system, transmitting and processing signals in the brain, is crucial, and the tested compound should not affect it. In this study, the human neuroblastoma cell line SH-SY5Y was used for neurotoxicity evaluation *in vitro*. The compounds showed moderate toxicity, with IC_50_ values in the range of 40.60–66.10 μM (**13** > **15** > **14**) (Table 2), i.e., at a concentration >1000-fold higher than the active ones toward 5-HT_6_R (*K*_i_, *K*_b_, Table 1). Since the compounds have a satisfactory safety profile and do not exhibit specific toxicity against SH-SY5Y cells, they can be considered suitable for further studies regarding their neuroprotective properties.

**Table 2:** IC_50_ Values Determined by Fitting a Sigmoidal Dose–Response Curve to the Data Using GraphPad Prism

**Compd**	**IC_50_^a^ ± SD (μM)**
**13**	40.60 ± 2.86
**14**	66.10 ± 6.24
**15**	53.20 ± 4.96

a The mean value of IC_50_ from the MTS assay in SH-SY5Y cells at 27 h of exposure. The IC_50_ value of each compound was defined as the concentration (μM) that caused 50% inhibition of cell viability in SH-SY5Y cells compared to vehicle-treated cells.

To investigate the neuroprotective effect of the compounds, rotenone, a toxin that blocks the mitochondrial electron transport chain by inhibiting complex I, was used;^103^ two methods were applied since rotenone impairs mitochondrial energy metabolism and increases ROS. The first method is an MTS-based viability assay (an improved version of MTT). The second method is based on ROS measurement with 2′,7′-dichlorofluorescin diacetate (2′7′DCFH_2_-DA). Despite the limitations associated with the use of fluorescent probes, particularly their lack of specificity for any specific ROS, this method is sufficient to gain a general understanding of oxidative stress and potential neuroprotective activity of the compounds of interest.

**Figure 5:**
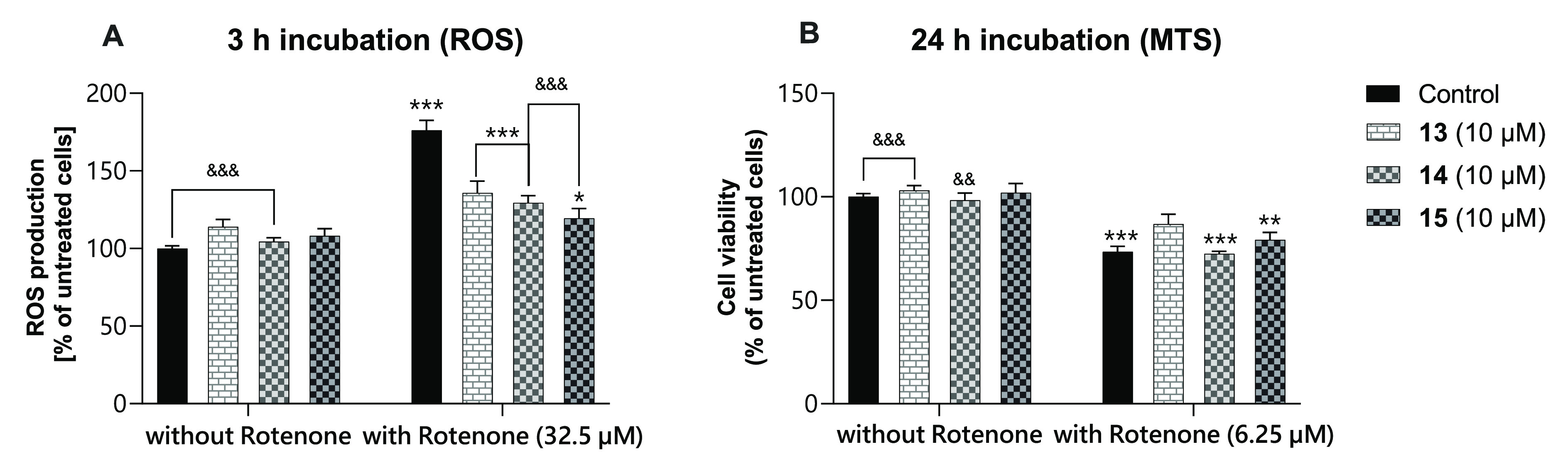
Neuroprotective effect of **13**–**15** on rotenone-induced neurotoxicity. SH-SY5Y cells were pretreated with the cited compounds for 1 h, and then rotenone at a concentration of 32.5 μM (A) or 6.25 μM (B) was added and incubated for further 3 h (A) or 24 h (B), respectively. The level of ROS is presented in panel A, and the metabolic activity of the cells is presented in panel B. One-way ANOVA determined the significance of the difference with the post-hoc Dunnett’s test (α = 0.05). *P < 0.05; **P < 0.01; ***P < 0.001 (vs control cells); ^&&^*P* < 0.01; ^&&&^*P* < 0.001 (vs rotenone-treated cells).

The results, shown in Figure 5, demonstrated that, when SH-SY5Y cells were pretreated with the tested compounds, the intracellular level of ROS after 3 h was 1.47 times lower compared to the level induced by rotenone alone. Treatment with the tested compounds alone did not affect ROS production. Contrary to the findings of the DCFH_2_-DA assay, we did not find such protective activity in the MTS assay. The metabolic activity of the cells was only slightly higher after pretreatment with **15** (79% vs 73% for rotenone, where the control is 100%) and the most visible after pretreatment with **13** (86% vs 73%). It is worth noting that the concentration of rotenone in this study was adjusted based on a dose–response curve of rotenone, in which a significant increase in ROS level and decrease in metabolic activity of SH-SY5Y cells *in vitro* were observed. Even though our studies examining rotenone-induced toxicity have employed short-term and relatively high levels of rotenone exposure, over a lifetime, the patients may have been exposed to the toxins for several years at relatively low concentrations.^104^

Summing up, the results of these assays confirm the neuroprotective properties of the tested compounds **13**–**15**, predominantly via inhibition of ROS production and the significantly lower effect on the metabolic activity of SH-SY5Y cells. The β-naphthyl dimethyl-branched derivative **15** turned out to be the relatively most potent neuroprotective agent in these studies. Furthermore, the trends of the action estimated in both assays suggest that the neuroprotective mechanisms are related to the antioxidant properties of **13**–**15**, which require a deeper insight.

### Antioxidative Mechanisms of Action for **13**–**15***In Vitro*

#### Total Antioxidant Capacity

**Figure 6:**
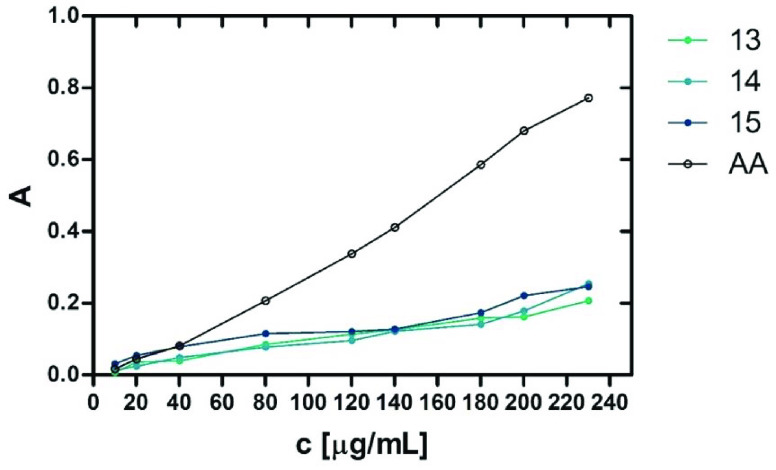
Absorbance (*A*) vs concentration (*c*, μg/mL) graphs of total antioxidant capacity for **13**–**15** vs reference ascorbic acid (AA).

In order to estimate the potential molecular mechanisms of the neuroprotective action found for **13**–**15***in vitro*, the total antioxidant capacity test was performed. To measure the oxidation power, the reaction with Mo(VI) was used, as described by Prieto et al.^105^ Compounds **13**–**15** all displayed total antioxidant capacity with different dose-dependent power, as shown in Figure 6 (Table S4), and the antioxidant potency increased with concentration. At higher concentrations, the increase for the tested selenium compounds **13**–**15** was much lower compared to the reference ascorbic acid (AA).

**Figure 7:**
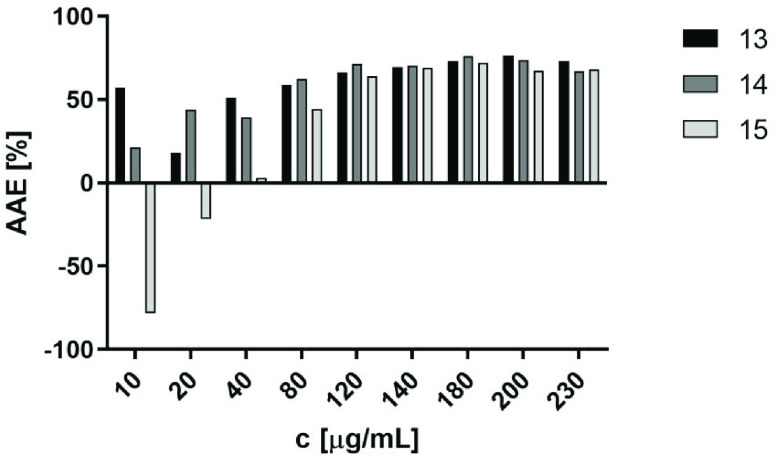
Graph of ascorbic acid equivalents (%AAE) for **13**, **14**, and **15** at different concentrations.

To better illustrate the antioxidative characteristics of each compound **13**, **14**, and **15**, the results expressed as ascorbic acid equivalents (AAE) are shown in Figure 7. In this way, the distinct difference of the reductive properties of **15** from those of **13** and **14** at the lower concentrations, which equalized at about 120 μg/mL, can be seen (for details, see Table S5).

In general, **15** turned out to be the relatively most potent at a lower concentration up to 40 μg/mL, displaying significantly stronger reductive action than that of reference ascorbic acid (AA) at their tested concentrations of 10–20 μg/mL, corresponding at 40 μg/mL, while it was less potent compared to AA at higher concentrations (80–230 μg/mL). It is worth noting that antioxidative action of **15** superior to that of AA was detected at concentration <40 μg/mL (∼ <90 μM), while actions that were neuroprotective in neuroblastoma and antagonistic for 5-HT_6_R were found at 10 μM and 15 nM, respectively. Although the concentrations used in the test did not reach the values corresponding to *K*_i_, the trend of the increasingly favorable AAE for **15** indicates that, with a further decrease in the concentration of **15** (10 μM or less), the antioxidant effect of **15** can still be significant and more potent than that of AA. This also suggests that antioxidant effects may contribute to the neuroprotective action confirmed for **15** at 10 μM in neuroblastoma cells in response to rotenone in neuroprotection assays (Figure 5).

On the other hand, results of the total antioxidant assays indicate that the antioxidative action of **15** grows only slightly with an increase in concentration—conditions which may guarantee antioxidant effects but showed cytotoxic effects, inhibiting the viability of neuroblastoma (Table 2). The IC_50_ value was estimated based on the MTS assay that evaluates the toxic effect of the compound, focusing on how it affects cell viability. The reduced cell viability corresponds to the reduced capacity of dehydrogenase to transform tetrazolium salt into formazan. One of the factors that may impair the function of mitochondrial dehydrogenase is the presence of ROS. The antioxidant action of **15** allows us to exclude oxidative toxicity mechanisms caused by this compound. At the cellular level, however, ROS might act as signaling molecules or cause cell damage. Which of these roles is undertaken depends on the equilibrium between ROS production and scavenging.^106^ It is worth noting that high levels of antioxidants (e.g., vitamin C) have been proposed to create a state of oxidation–antioxidation imbalance that could disrupt the physiological activities of ROS.^107,108^ In this frame, the antioxidative capacity of **15**, significantly higher than that of AA at concentrations around toxic levels, may contribute to the inhibition of viability of the neuroblastoma cell observed in our neurotoxicity test (Table 2). Nevertheless, this hypothesis refers to concentrations significantly higher than the 5-HT_6_R *K*_i_ value.

Thus, the total antioxidant capacity found for **15** seems to be promising for potential dual, neuroprotective/antioxidative, and 5-HT_6_R antagonistic actions, which may enhance the therapeutic efficacy desired in the treatment of AD. In the cases of **13** and **14**, the assay also confirmed the noticeable reductive action, but both compounds were ∼2–3-fold less potent in comparison to AA along all tested concentrations (10–230 μg/mL).

To conclude, the results of the total antioxidant capacity assay confirmed a favorable dose-dependent reductive activity for **13**–**15**, which seems to be the main (bio)chemical reason for the neuroprotective effects observed in the *in vitro* neuroblastoma model. The capacity of the dimethyl-branched β-naphthyl derivative **15** was especially beneficial and superior with respect to the other two tested compounds, **13** and **14**, due to maintaining the reductive effects at lower concentrations, closer to the pharmacologically active doses.

#### Thiophenol Assay

**Figure 8:**
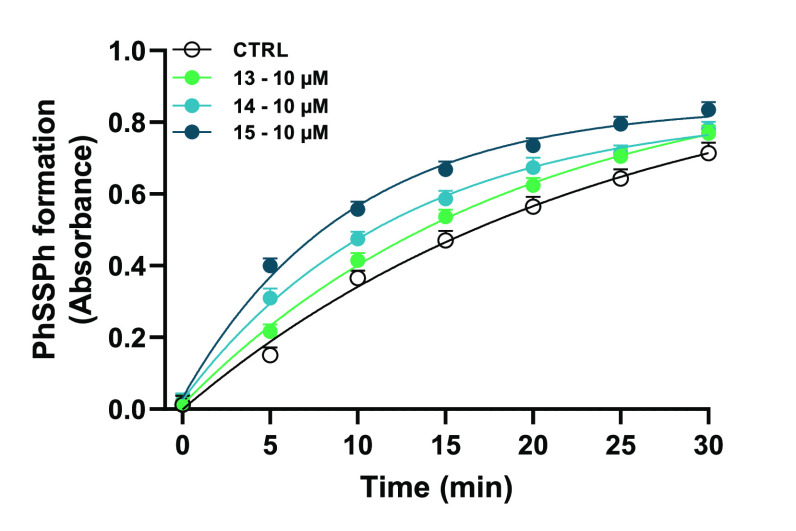
PhSSPh formation with respect to time in the presence and absence of the catalysts **13**–**15**. The reactions were carried out at 25 °C using PhSH (10 mM) in methanol, catalysts (10 μM), and H_2_O_2_ (37.5 mM). Data are expressed as mean ± SEM of at least three independent experiments.

For a deeper insight into molecular mechanisms of neuroprotection, the GPx-like activity for the β-naphthyl Se-ethers **13**–**15** was tested with a thiophenol assay, as described by Mouithys-Mickalad et al.^109^ All the compounds showed a GPx-like activity, although with different potency (Table 3, Figure 8). Indeed, **15** was the most effective catalyst, showing a reaction rate (*t*_1/2_) 2.5-fold lower than that of the control, followed by **14** and **13** (reaction rates 2- and 1.3-fold lower with respect to control). The rate constant (*K*) values were in agreement with those of the reaction rate and highlighted the strong effect of **15** (Table 3).

**Table 3:** Reaction Rates (*t*_1/2_) and Rate Constants (*K*) of the Reduction of Hydrogen Peroxide (37.5 mM) with Benzyl Thiol (10 mM) in the Presence of the **13**–**15** at a Concentration of 10 μM^a^

**Compd**	***t*_1/2_ (min)**	***K* (s^–1^)**
Control	16.52 ± 1.40	2.54 ± 0.50
**13**	13.11 ± 0.70	3.20 ± 0.19
**14**	8.60 ± 0.23**	4.85 ± 0.20 ***
**15**	6.52 ± 0.18***	6.42 ± 0.17***

a Data are expressed as mean ± SEM of at least three independent experiments. **p < 0.01 and ***p < 0.001 vs control (one-way ANOVA followed by Dunnett’s multiple comparison post-test).

Glutathione peroxidases, especially GPx4, are responsible for protecting cells from death through ferroptosis, and decreased expression of these enzymes has been reported in AD patients.^110^ Therefore, the GPx-like activity of studied compounds **13**–**15** suggests that they are capable of protecting cells from oxidative damage and possibly could decrease neuronal loss in AD.

The results of the thiophenol assay, in accordance with those coming from the total antioxidant capacity test, confirmed the antioxidative properties of the tested Se-ether triazines **13**–**15**. Thus, due to the chemical properties found for **13**–**15**, they may contribute to miscellaneous molecular mechanisms responsible for neuroprotective effects, which can be applicable for potential treatments of neurodegenerative diseases such as AD. Considering the results of both tests, **15** showed the most beneficial effects and, therefore, seems to be the most suitable candidate for further extended pharmacological screening.

### Antioxidant and Pro-inflammatory Gene Expression and NRF2 Localization Regulated by **15**

In line with biochemical data, a gene expression profile in SH-SY5Y cells upon treatment with **13**–**15** (and donepezil as reference) at two different concentrations (1 μM and 10 μM) was examined.

**Figure 9:**
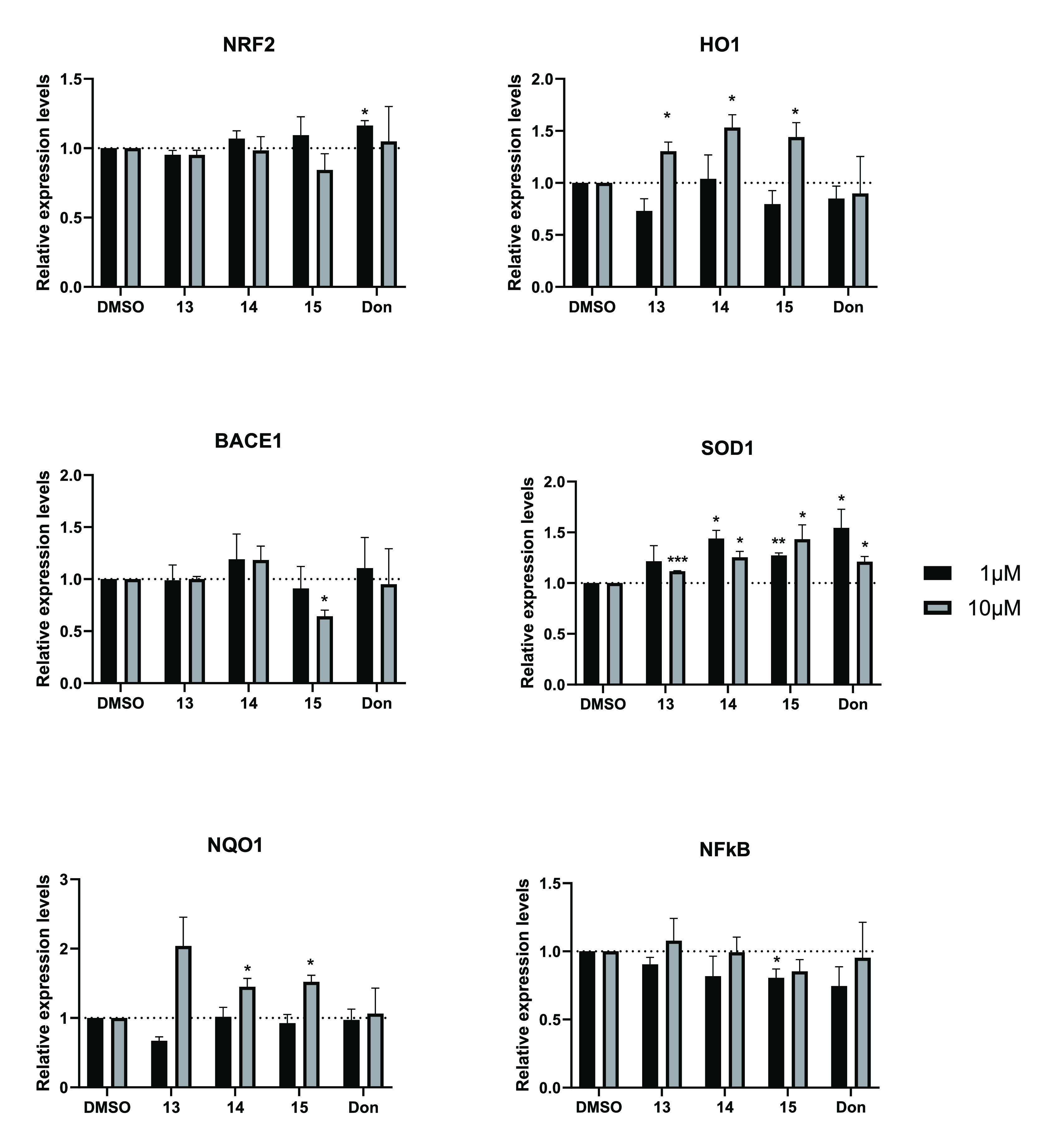
qRT-PCR analysis for the indicated transcripts in SH-SY5Y treated with **13**, **14**, **15**, and donepezil at 1 μM (black columns) or 10 μM (gray columns) for 24 h. DMSO (control) represent the cells treated with the vehicle. The values are calculated by the 2(−Δ*Ct*) method, expressed as fold of expression vs the control (arbitrary value = 1) and shown as mean ± SEM. Statistically significant differences are reported (*p < 0.05; **p < 0.01; ***p < 0.001) for three independent experiments.

Specifically, the expression of the antioxidant genes HO-1, SOD-1, and NQO-1 was induced in response to the treatment, while the expression of nuclear factor kappa B (NFkB, related to cell inflammation) was impaired. Notably, with respect to **15**, also the expression of BACE1 was impaired (Figure 9).

These data confirmed that antioxidant and pro-inflammatory genes were regulated in response to the tested compounds **13**–**15** and that their effects are even more evident with respect to that of donepezil.

Notably, the mRNA expression of NRF2 was not significantly affected by the cited compounds, despite the previously shown genes being directly regulated by the NRF2 pathway. NRF2 reduces oxidative stress in response to the detection of both ROS and RNS (reactive nitrogen species). In these conditions, the NRF2 protein translocates from the cytoplasm into the nucleus, where its binding to the antioxidant response elements (AREs) regulates the expression of several genes, including the antioxidant and anti-inflammatory ones. In AD, NRF2 nuclear translocation is impaired together with synaptic plasticity and memory.^111^

**Figure 10:**
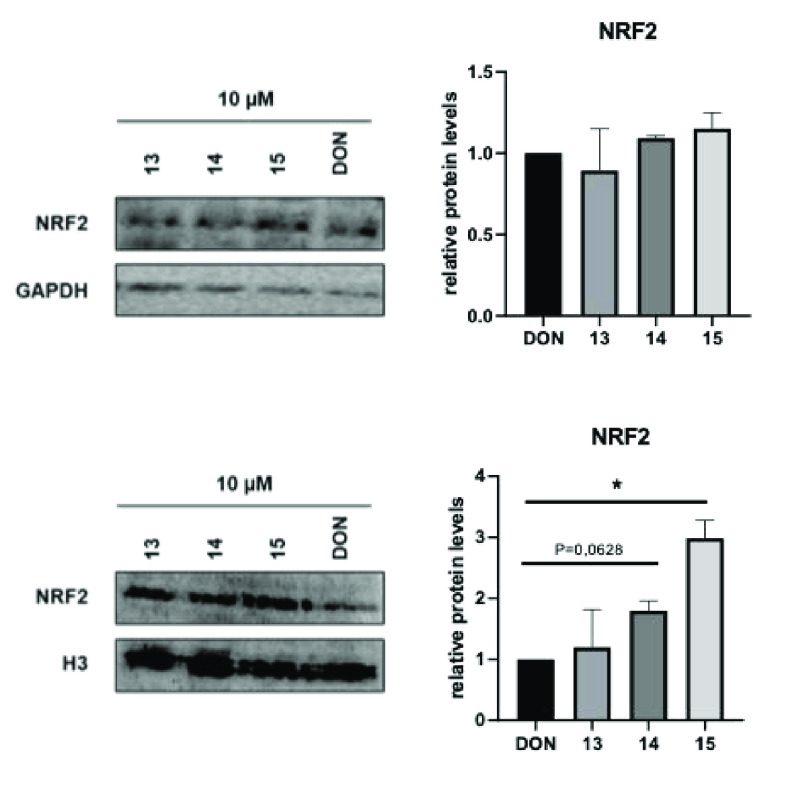
Western blotting of NRF2 and GAPDH (total proteins’ normalizer) (Top) or H_3_ (nuclear proteins’ normalizer) (bottom) and their respective densitometric analysis (right panels) in the SH-SY5Y cell line upon treatment with 10 μM **13**, **14**, **15**, and donepezil (**DON**) for 24 h. *p*-values were obtained using Student’s *t*-test (*p < 0.05) for two independent experiments.

In line with this knowledge about NRF2, its nuclear localization was assessed through a differential protein extraction (nucleus/cytoplasm). While the NRF2 cytoplasmic abundance was not significantly affected by the treatment with the tested compounds, NRF2 nuclear expression is significantly higher in **15**-treated cells with respect to the other compounds and, more interestingly, with respect to donepezil at the same concentration (Figure 10).

Therefore, based on these data, **15** is demonstrated as an inducer of NRF2 nuclear translocation, and this localization is *bona fide* related to the observed transcriptional regulation of antioxidant and anti-inflammatory genes. In conclusion, this compound should be a potential candidate for novel therapeutic approaches for neurodegenerative diseases, including AD.

### *In Vitro* ADME for **13–15**

#### Permeability

The parallel artificial membrane permeability assay (PAMPA) was used to quantify the passive diffusion of substances through synthetic membranes, whose composition has been optimized to model the permeability behavior of phospholipid-based biomembranes.^112^ In the context of the blood–brain barrier (BBB) permeability, the PAMPA provides insights into how efficiently compounds can cross the BBB. Compounds demonstrating high permeability in the PAMPA for BBB are more likely to reach the brain tissue. This is crucial for drugs targeting neurological disorders and CNS-related conditions, as effective BBB penetration is necessary for the drug to exert its therapeutic effects within the brain. By measuring the rate at which the compound crosses this membrane, researchers can infer its potential to cross the actual BBB *in vivo*. As shown in Table 4, all tested compounds have an excellent permeability (*Pe*) with the highest value of 10.54 ± 4.85 for **14**. Interestingly, **13** had only a slightly lower *Pe* value of 7.31 ± 1.94 with a lower retention mass (14% vs 47%) simultaneously. All test compounds are likely to penetrate the BBB efficiently, making them promising candidates for drug development targeting the CNS.

**Table 4:** Permeability Coefficient of **13–15** and Caffeine (as a Control)

**Compd**	***P*_e_^a^^,^^b^ (×10^–6^ cm/s) ± SD**	**Drug retention (%)**
**13**	7.31 ± 1.94	14
**14**	10.54 ± 4.85	47
**15**	6.79 ± 1.27	46
caffeine	2.35 ± 0.02	21

a PAMPA plate’s manufacturer breakpoint for permeable compounds: *P*_e_ ≥ 1.5 × 10^–6^ cm/s.

b Tested in triplicate.

#### Metabolic Stability

Compounds **13**–**15** were also investigated to determine their metabolic stability in a rat liver microsome (rLM) model.^97^ Compound **14** showed good metabolic stability above 75% (**14**: 77.64%, Table 5). Furthermore, the number of metabolites formed was as low as three. **13** and **15** proved to be metabolically unstable in rats (only 53.33% of **13** and 36.72% of **15** remained in the reaction mixture, Table 5). The numbers of metabolites formed reached two (in case of **15**, with one biotransformation product accounting for the majority in the mixture analyzed) and three (in case of **13**). For comparative purposes, the AD drug, donepezil, was also tested and exhibited partial metabolism (75.13% of the parent compound remained in the reaction mixture). The predicted metabolic pathways indicated demethylation and *N-*oxidation, similar to findings already reported in the literature.^113^

**Table 5:** Metabolic Stability Test Results for **13**–**15**

**Compd**	**% remaining in reaction mixture**	**Molecular mass [*m*/*z*]**	***t*_R_ (min)**	**Metabolite**	**% remaining in reaction mixture**	**Molecular mass [*m*/*z*]**	***t*_R_ (min)**	**Proposed metabolic pathway**
**Rat Liver Microsomes**								
**13**	53.33	429.27	4.98	M_1_	34.98	445.10	5.20	hydroxylation
				M_2_	10.31	415.17	4.84	demethylation
				M_3_	1.37	431.07	5.38	demethylation and hydroxylation

**14**	77.64	443.10	5.36	M_1_	12.94	429.20	5.22	demethylation
				M_2_	8.33	459.13	5.59	hydroxylation
				M_3_	1.09	462.53	5.63	demethylation and double hydroxylation

**15**	36.72	443.37	5.38	M_1_	54.88	429.20	5.20	demethylation
				M_2_	8.40	459.20	5.60	hydroxylation

donepezil	75.13	380.31	4.94	M_1_	11.34	396.35	5.12	*N*-oxidation
				M_2_	8.22	290.25	3.79	fragmentation
				M_3_	3.90	366.15	4.49	*O*-demethylation
				M_4_	0.89	366.28	4.56	*O*-demethylation
				M_5_	0.52	396.21	4.41	*O*-demethylation

**Human Liver Microsomes**								
**15**	68.93	442.97	5.38	M_1_	16.05	429.20	5.21	demethylation
				M_2_	4.57	459.13	5.62	hydroxylation
				M_3_	4.03	397.28	4.62	fragmentation
				M_4_	2.05	399.34	6.02	fragmentation
				M_5_	1.56	399.14	3.82	fragmentation
				M_6_–M_9_				unidentified

Compound **15**, the most promising from a therapeutic point of view, was additionally tested in human liver microsomes. The results obtained indicate much better metabolic stability of **15** tested in human than in rLMs, where the compound underwent only 31% biotransformation, with the formation of numerous (nine) but negligible metabolites (Table 5).

Mass spectral analysis supported by *in silico* data allowed us to determine the most probable metabolic pathways of the tested ligands, which are hydroxylation at the naphthyl ring and demethylation at piperazine (Figures S5–S23).

These studies allowed us to examine the sensitivity of C(sp^3^)–Se bonds in the oxidative conditions of liver microsomes. The results of detailed analyses of mass spectra for intact **13**–**15** and their metabolites (Table S7) indicate high stability along the C(sp^3^)–Se bonds of the highly active 5-HT_6_R Se ligands (**13**–**15**) in the oxidative conditions of liver microsomes, corresponding to potential therapeutic effects in rats and humans.

In general, the ADME *in vitro* results predict promising PK properties for the β-naphthyl selenoethers **13**–**15**. Such properties, combined with a low risk of neurotoxicity but comprehensive neuroprotective effects confirmed *in vitro*, promote these compounds for advanced preclinical studies in the search for a drug candidate. In light of the whole *in vitro* screening carried out so far, the dimethyl branched β-naphthyl selenoether **15** demonstrated favorable properties; thus, it was selected for extended *in vitro* ADME and further *in vivo* assays in rats.

### Extended *In Vitro* ADME for **15**

#### Clearance in Rat and Human Microsomes

The preliminary data on metabolic stability in rat and human liver microsomes of **15** encouraged us to conduct more precise research in order to determine the PK parameters *in vitro*, such as intrinsic clearance (CL_int_) and half-life (*t*_1/2_) for **15**. The obtained data (Table 6, Figures S20–S23) confirmed higher metabolic stability of **15** in the presence of human liver microsomes than in rat ones. The calculated CL_int_ and *t*_1/2_ in humans were 3- and 2-fold higher than in rats, respectively. Higher metabolic stability in humans compared to rats is generally preferred in drug development. Compounds that are metabolically stable in humans are less likely to interact with other drugs by interfering with or inducing drug-metabolizing enzymes, especially from the cytochrome P450 family. This reduces the potential for adverse drug–drug interactions, which can be a significant safety concern. Furthermore, higher metabolic stability in humans helps maintain consistent drug exposure levels over time. This is critical for achieving the desired therapeutic effect and for ensuring that patients receive a consistent and predictable dose of the drug. Last but not least, from the point of medicinal chemistry, greater metabolic stability in humans can simplify the drug development process, as it reduces the need for extensive modifications to enhance stability and predictability in human subjects. This can lead to faster and more cost-effective drug development.^114^

**Table 6:** Comparison of *In Vitro* Pharmacokinetic Parameters in Different Matrices for **15**

**Matrix**	**CL_int_ (mL/min/kg)**	***t*_1/2_ (min)**
Rat liver microsomes	139.3	8.9
Human liver microsomes	47.5	17.1

#### Action on Receptor Off-Targets

Achieving selectivity across a broader spectrum of receptors contributing to CNS regulation is a crucial determinant of the pharmacological attributes of the most promising compound (**15**) within our series. Thus, we additionally assessed the activity of **15** against important CNS receptors, i.e., histamine H_3_, muscarinic acetylcholine M_1_, cannabinoid CB_1_, α_2_-adrenergic (α_2_-AR), and NMDA receptors. Subsequently, we conducted RBAs on the aforementioned receptors to rule out the potential for pharmacological interactions (Table 7).

**Table 7:** Radioligand Binding Assay for M_1_, H_3_, α_2_-AR, CB_1_, and NMDA Receptor^a^

	**Receptor and % of control specific binding**				
**Compd**	**M_1_**	**H_3_**	**α_2_-AR**	**CB_1_**	**NMDA**
**15**	6%	0%	15%	4%	0%
Atropine	99%				
Pirenzepine	90%				
Methoctramine	44%				
Scopolamine	100%				
(*R*)-(−)-α-Methylhistamine		99%			
Pitolisant		71%			
Clonidine			98%		
(*R*)-(+)-WIN55,212-2				85%	
AM251				100%	
MK-801					100%

a Results are presented as a percentage of control specific binding at 1 μM concentration of **15** and reference compounds.

Our investigations, employing a compound concentration of 1 μM, revealed negligible impact, with receptor effects falling much below the 50% threshold and ranging between 0% and 15%. This observation implies a lack of sufficient action to perform full dose–response binding assays. Consequently, our findings confirm the significant selectivity of **15** in its interaction with the 5-HT_6_R within a broader receptor panel.

#### Toxic Effects on HepG2 and HEK-293

**Figure 11:**
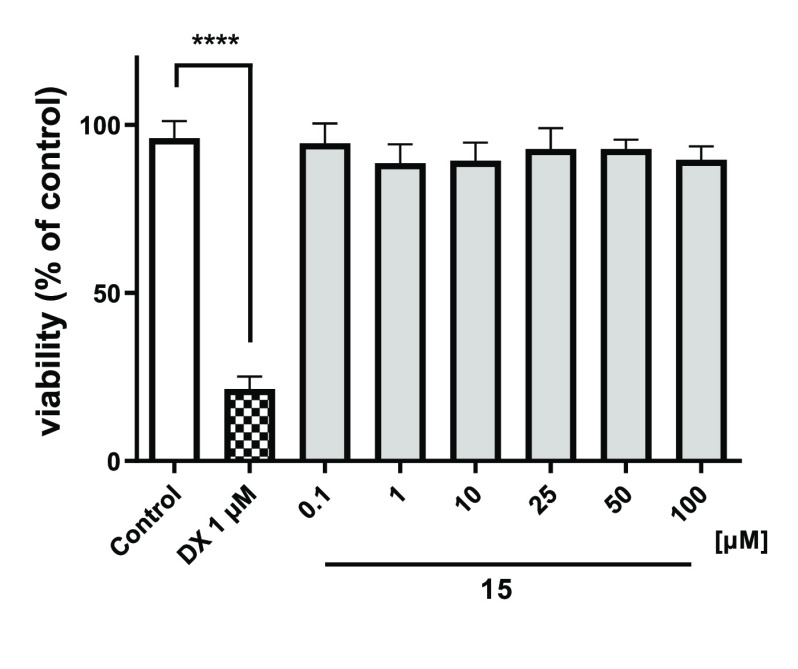
Viability of HepG2 cells after 72 h of incubation in the presence of **15** and the reference drug doxorubicin (DX, 1 μM). The statistical significance (GraphPad Prism 8.0.1) was evaluated by a one-way ANOVA, followed by Bonferroni’s Comparison Test. ****p < 0.0001 compared with control (DMSO 1% in growth media).

**Figure 12:**
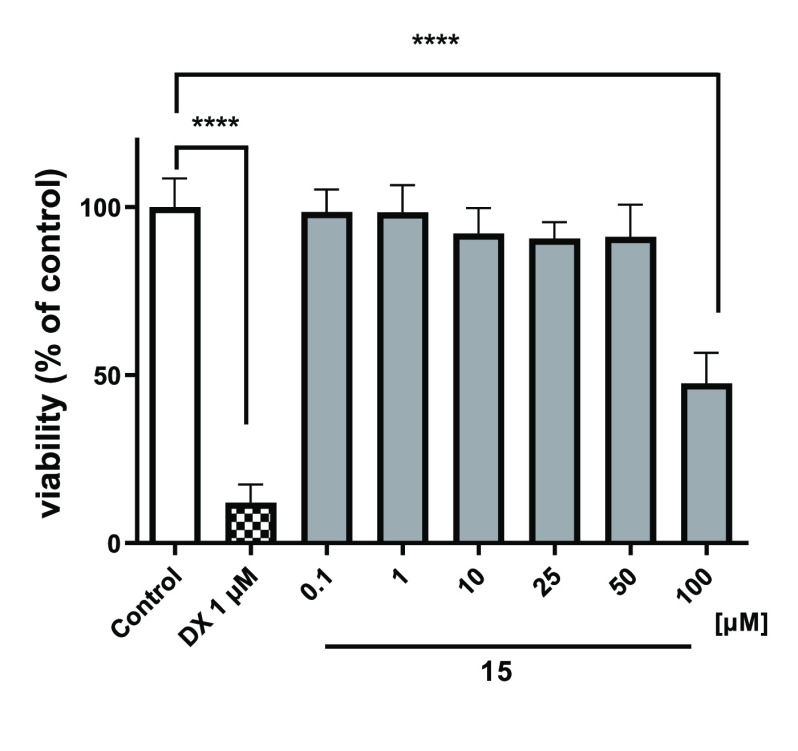
Viability of HEK-293 cells after 72 h of incubation in the presence of **15** and the reference drug doxorubicin (DX, 1 μM). The statistical significance (GraphPad Prism 8.0.1) was evaluated by a one-way ANOVA, followed by Bonferroni’s Comparison Test. ****p < 0.0001 compared with control (DMSO 1% in growth media).

In terms of determining the safety profile of **15** in more detail, hepatotoxic and nephrotoxic effects of the compound in HepG2 and HEK-293 cell lines, respectively, were investigated. Our assessments revealed that **15** did not exhibit any hepatotoxic effects across the tested concentration range (0.1–100 μM) in HepG2 cells, and similarly, it did not exert any adverse effects on HEK-293 cells within the concentration range of 0.1–50 μM (Figures 11 and 12). Consequently, based on these findings, **15** can be considered safe regarding hepatotoxic and nephrotoxic concerns.

#### hERG Inhibitory Properties

In the next step, **15** was investigated for its hERG inhibitory properties *in vitro*. In the light of the results obtained, **15** showed moderate potency to inhibit the activity of the hERG channel. The half-maximal inhibitory concentration (IC_50_) determined for **15** in the functional assay using the QPatch automated patch clamp system was 1.36 ± 0.16 μM (Table 8, Figure S24). Despite the ability to modulate the channel activity in micromolar concentration, the inhibitory potency for this seleno compound was still lower in comparison to that of a safe, marketed drug, verapamil (IC_50_ hERG = 0.419 ± 0.031 μM), serving as reference hERG modulator in the current study, which indicates the relatively good cardiac safety profile of **15**.

**Table 8:** Inhibitory Potencies of **15** and Verapamil at Human Recombinant hERG Potassium Channel

**Compd**	**hERG channel inhibition IC_50_ ± SEM (μM)**
**15**	1.36 ± 0.16
Verapamil	0.419 ± 0.031

#### Mutagenicity Assay

**Figure 13:**
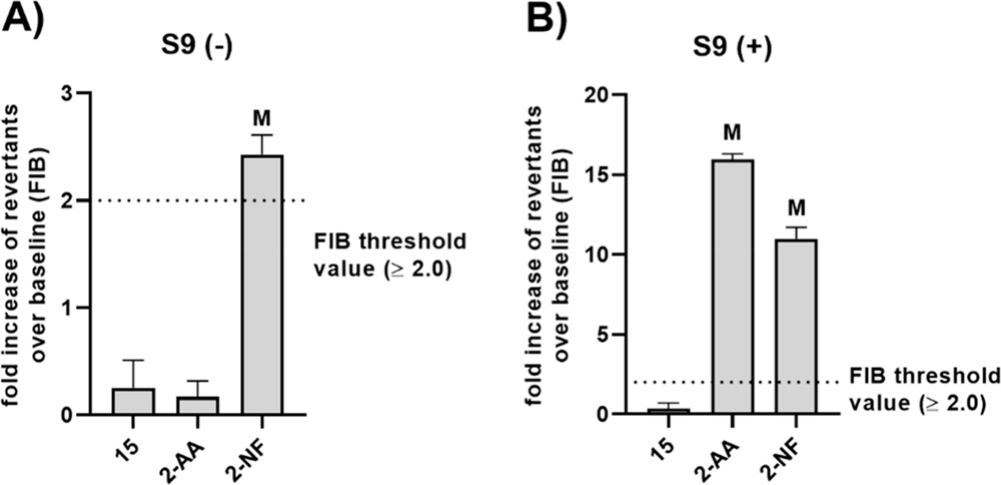
Ames mutagenicity test. Fold increase of histidine prototrophy revertants over baseline (FIB) ± SD (*n* = 3) for *Salmonella typhimurium* strain TA98, exposed to 10 μM concentration of **15**, 2-aminoanthracene (2-AA), or 2-nitrofluorene (2-NF) in the absence (A) or presence (B) of rat liver S9 fraction. M, mutagenic action observed (FIB ≥ 2.0).

As the last *in vitro* assay on safety, **15** was tested for potential mutagenic activity in an Ames test with *Salmonella typhimurium* strain TA98, which proved its good safety profile (Figure 13A). Ames assay was also conducted for **15** in the presence of rat liver S9 fraction, containing cytosolic and microsomal enzymes, to verify if metabolic activation of **15** would induce its mutagenicity. Performed experiments showed that the metabolites of **15** also do not increase the mutation rate in the tested *S. typhimurium* strain (Figure 13B). At the same time, the conducted assay confirmed that the reference mutagen, 2-nitrofluorene, causes a significant increase in the number of shift mutations in the utilized bacterial strain and that 2-aminoanthracene requires metabolic activation to gain mutagenic properties.

### *In Vivo* ADMET for **15**

The aim of the PK studies was the first assessment of the basic PK parameters of the tested compound **15** in a representative species of rodents (rats that are small enough not to require much compound but large enough for a large volume of blood and organs) and the determination of their penetration into various organs (heart, lungs, liver, kidneys), including the target organ (brain). Rats were administered with **15** at a single dose of 1 mg/kg intraperitoneal (i.p.), determined in behavioral studies, because this route is most often used in our laboratory for *in vivo* screening of new compounds, with low impact of stress on laboratory rodents.

**Figure 14:**
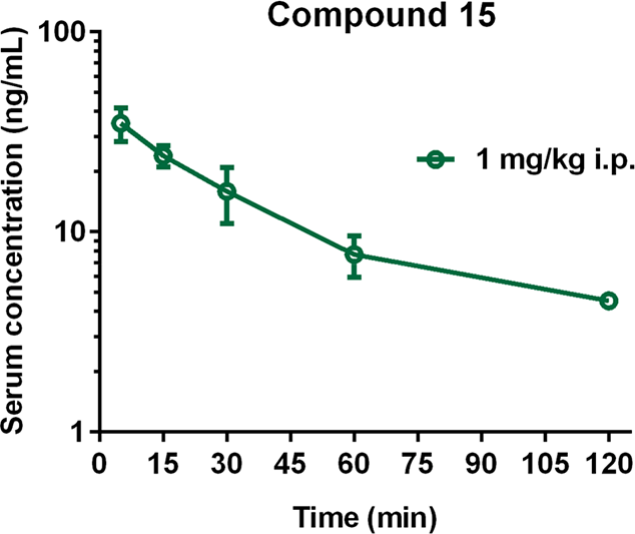
Serum concentration–time profiles of **15** following i.p. administration to rats at a dose of 1 mg/kg (mean ± SD, *n* = 3–4).

Compound **15**’s mean serum concentration–time profiles and key PK parameters were calculated using a non-compartmental approach. Figure 14 shows that **15** administered at a dose of 1 mg/kg i.p. in male Wistar rats (200–230 g) was rapidly absorbed from the peritoneal cavity (*T*_max_ = 5 min), whereas *C*_max_ was 35 ng/mL. The area under the concentration–time curve from the time of dosing to the time of the last measurable concentration (AUC_0–*t*_) for serum was 1404.8 ng·min/mL. The apparent volume of distribution (*V*_*z*_/*F*) during the terminal phase was 54.8 L/kg, and clearance (CL/*F*) was 32.6 L/h/kg. The tested compound was characterized by a slow terminal elimination, resulting in a favorable value for serum elimination half-time (*t*_0.5λ*_z_*_ = 69 min, Table 9).

**Table 9:** Pharmacokinetic Parameters^a^ of **15** in Serum Following 1 mg/kg i.p. Administration to Rats, Assessed Using Non-compartmental Analysis (Mean ± SD, *n* = 3–4)

*C*_max_ (ng/mL)	34.96 ± 6.67
*T*_max_ (min)	5.00 ± 0.00
AUC_0–*t*_ (ng·min/mL)	1404.79 ± 278.44
*V*_*z*_/*F* (L/kg)	54.79 ± 19.21
CL/*F* (L/h/kg)	32.64 ± 3.16
λ_*z*_ (min^–1^)	0.0106 ± 0.0028
*t*_0.5λ*_z_*_ (min)	68.66 ± 17.87
MRT (min)	83.98 ± 21.05

a *C*_max_, maximum concentration; *T*_max_, time to reach the maximum concentration; AUC_0–*t*_, area under the serum concentration–time curve from the time of dosing to the time of the last measurable concentration; *V*_*z*_/*F*, volume of distribution at the elimination phase; CL/*F*, oral clearance; λ_*z*_, terminal elimination rate constant, calculated using log-linear regression of the terminal portions of the serum concentration–time curves; *t*_0.5λ*_z_*_, half-life in the elimination phase; MRT, mean residence time.

**Figure 15:**
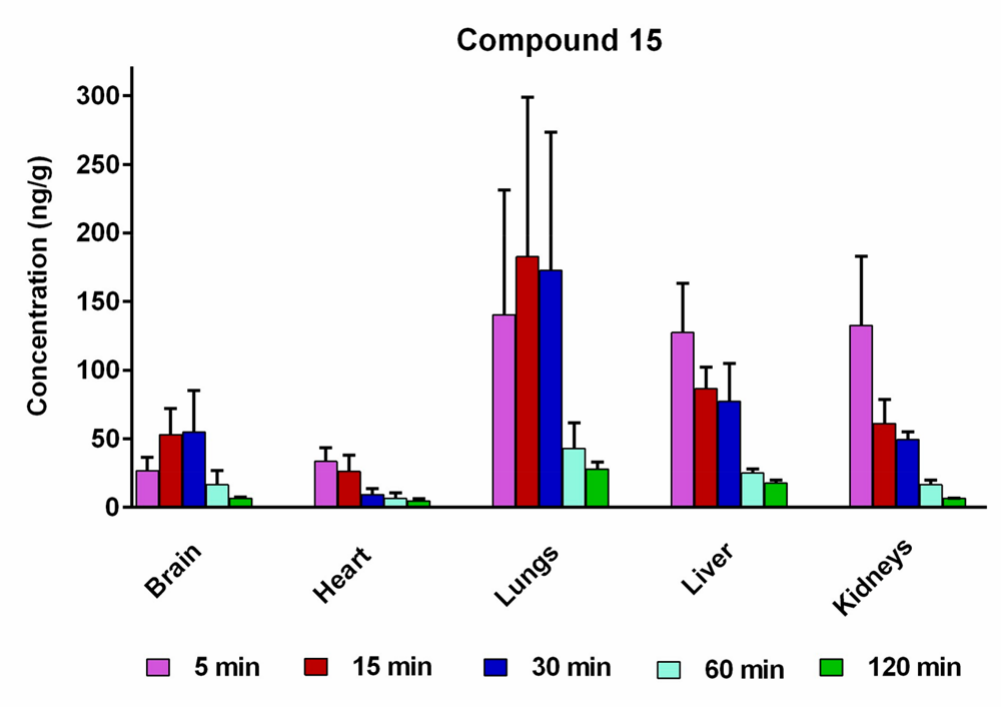
Concentrations of **15** in rat tissues at 5, 15, 30, 60, and 120 min after i.p. administration of 1 mg/kg of **15** in rats (mean ± SD, *n* = 3–4).

The tissue distribution of **15** was assessed at five different time points (5, 15, 30, 60, and 120 min) after single i.p. administration at the dose of 1 mg/kg in rats (*n* = 3–4 per time point). The results are presented in Figure 15.

The tissue distribution profiles of **15** reveal that this compound was rapidly absorbed and readily diffused throughout all analyzed tissues (Figure 15). Following its i.p. administration, *C*_max_ for **15** was observed in heart, liver, and kidney tissues at 5 min, in lung tissue at 15 min, and in brain tissue at 20 min post-dosing, after which the concentrations declined markedly over the next 60–120 min, indicating that the tested compound does not accumulate substantially in any of the analyzed tissues.

Notably, the concentrations of **15** in brain tissue were highest, reaching up to 52.94 and 54.87 ng/g after 15 and 30 min, respectively (Figure 15). The concentrations determined in serum at these points were 2 and 3 times lower, respectively (Figure 14), suggesting that **15** can efficiently cross the BBB. The compound concentrations under investigation in the heart tissue were much lower than in the tissues mentioned above. The calculated tissue-to-serum AUC ratios (*K*_p_) followed the same pattern as the values of *C*_max_, reaching the highest value for lung tissue and the lowest for heart tissue (Table 10).

**Table 10:** Pharmacokinetic Parameters^a^ of **15** in Tissues Following 1 mg/kg i.p. Administration to Rats, Assessed Using Non-compartmental Analysis (Mean ± SD, *n* = 3–4)

	**Brain**	**Heart**	**Lungs**	**Liver**	**Kidneys**
*C*_max_ (ng/g) ± SD	58.89 ± 26.4	33.56 ± 10.1	182.69 ± 68.3	127.40 ± 35.8	132.64 ± 50.3
*T*_max_ (min) ± SD	20 ± 8.7	5 ± 0.0	15 ± 0.0	5 ± 0.0	5 ± 0.0
AUC_0–*t*_ (ng·min/g) ± SD	3038.86 ± 1426.9	1238.51 ± 537.6	9992.23 ± 4724.8	5432.84 ± 1251.6	3815.50 ± 850.9
λ_*z*_ (min^–1^) ± SD	0.013 ± 0.0039	0.018 ± 0.0047	0.0084 ± 0.0036	0.0082 ± 0.00074	0.016 ± 0.0030
*t*_0.5λ*_z_*_ (min) ± SD	58.64 ± 20.5	41.37 ± 12.8	94.68 ± 45.4	85.13 ± 7.4	43.97 ± 8.3
MRT (min) ± SD	71.04 ± 26.2	65.81 ± 8.5	108.24 ± 58.7	96.70 ± 2.7	47.70 ± 9.1
*K*_p_ ± SD	2.16 ± 0.63	0.88 ± 0.21	6.85 ± 1.98	3.87 ± 0.18	2.72 ± 0.07

a *C*_max_ , maximum concentration; *T*_max_, time to reach the maximum concentration; AUC_0–*t*_, area under the serum concentration–time curve from the time of dosing to the time of the last measurable concentration; λ_*z*_, terminal elimination rate constant, calculated using log-linear regression of the terminal portions of the serum concentration–time curves; *t*_0.5λ*_z_*_, half-life in the elimination phase; MRT, mean residence time; *K*_p_, tissue-to-serum AUC ratio.

**Figure 16:**
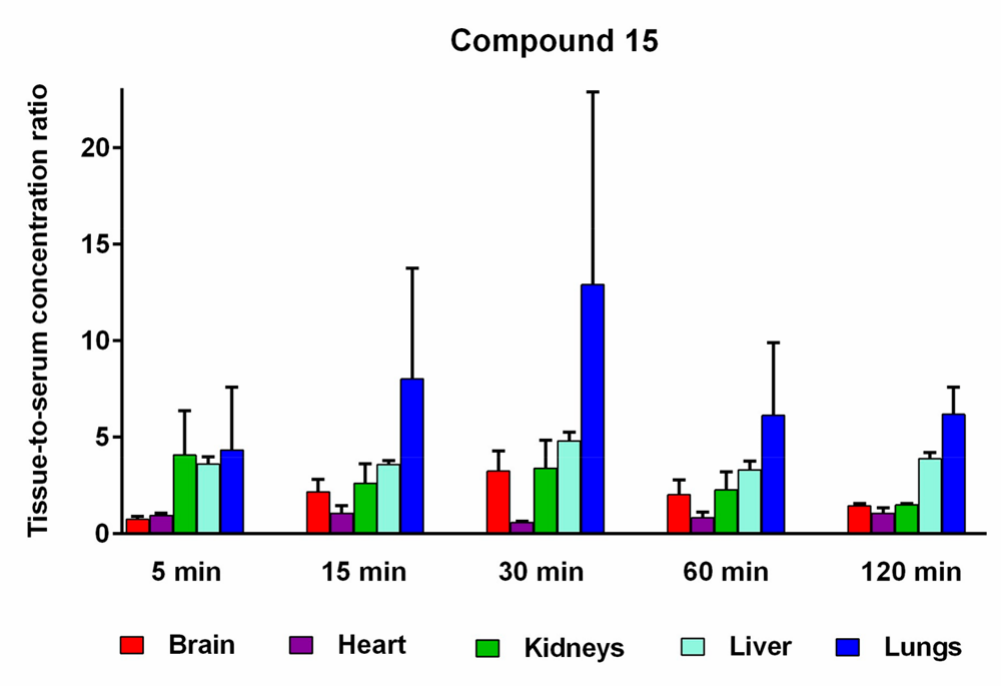
Tissue-to-serum concentration ratios of **15** following i.p. administration of a dose of 1 mg/kg (mean ± SD, *n* = 3–4).

The elimination rates of **15** from brain tissue were similar to those observed in serum. Exceptions were the kidneys and heart, where the terminal half-life was shorter than those in serum and other tissues tested. Similarly, mean residence time (MRT) values were the shortest in these organs, and the highest values of this parameter were observed in the lungs (Table 10). As expected, tissue-to-serum concentration ratios (Figure 16) were very low for the heart, and the highest values were noted for the lungs.

The results of these *in vivo* studies allowed us to extend the knowledge about the ADME properties of **15** initially estimated *in vitro*. The studied compound **15** demonstrated an excellent permeability, with a *Pe* value >2-fold higher than that estimated for well-permeable caffeine (Table 4). In accordance with the high permeability found in the PAMPA, the PK parameters evaluated *in vivo* indicated rapid absorption of **15** from the extensive distribution in the peritoneal cavity (*V*/*F* = 54.8 L/kg) (Table 9).

Furthermore, the high serum clearance (CL/*F* = 32.6 L/h/kg) and a mean *t*_0.5λ*_z_*_ of 69 min can be observed, surpassing the clearance rates of other drugs commonly used for AD, such as donepezil (1 mL/min/kg i.m.), rivastigmine (0.69 mL/min/kg i.m.), and memantine (211 mL/min/kg p.o.). This suggests that in young male rats, following single administrations, there is no significant long-term accumulation of **15**. However, it is important to consider that in older rats, the CL/*F* of **15** may exhibit a different, potentially lower value, as confirmed by published data on age-related PK differences observed with donepezil.^115^ Moreover, it is worth noting that multiple doses of **15** could potentially lead to a reduction in CL/*F*, as observed in other studies, such as those involving memantine, where clearance decreased by 1.73-fold after oral administration of multiple doses.^116^ Given that drugs for AD are predominantly used in older populations and significant PK changes occur with age, including reductions in renal and hepatic clearance,^117^ these findings are essential for a comprehensive understanding of drug behavior in this context. Beyond age-related differences, it is substantial to recognize that the clearance of compounds in rats may vary from that in human patients due to differences in enzymatic activity. To address the issue of species-dependent clearance for **15**, *in vitro* studies were conducted to assess clearance (CL and *t*_1/2_) in both human and rat microsomes, as described above. The results of those studies (Table 6) reveal that the clearance in the human microsome model is approximately 3 times slower than that in rat microsomes, offering an encouraging prognosis for a similar relationship in an *in vivo* setting.

On the other hand, the volume of distribution of **15** was approximately 82-fold greater than the average volume of rat total body water (0.67 L/kg),^118^ suggesting that **15** could be extensively distributed in the tissues and organs with a large degree of tissue binding. The maximum concentration in rat brain was observed slightly later than in serum and was higher until the end of the monitoring (Figures 14 and 15). This behavior may suggest favorable properties of **15** to maintain a therapeutic concentration longer. In addition, the brain-to-serum AUC ratio (*K*_p,brain_) is the most widely used *in vivo* parameter to classify compounds regarding CNS distribution, where *K*_p,brain_ = 1 is used as a cutoff.^119^ Thus, the *K*_p,brain_ = 2.16 for **15** (Table 10) indicates an excellent BBB penetration. The confirmed *in vivo* presence of **15** in brain tissue (Table 10, Figure 15) is essential for potential drugs in the treatment of AD, in particular for those acting via 5-HT_6_R, which is present almost exclusively in the CNS. In addition, good penetration into the brain is crucial for the various neuroprotective mechanisms found for **15***in vitro* to translate into the desired therapeutic effects, i.e., inhibiting neurodegeneration.

According to our preliminary experiments, **15** is mainly distributed to the most abundant blood-supply tissues, such as lungs, kidneys, and liver, which implies that the distribution of **15** might depend on the blood flow and perfusion rate of the organ. These findings may also indicate that **15** is metabolized and excreted via these organs. According to the concentration–time profiles examined in liver and kidneys, **15** decreased more rapidly in kidneys than in liver, which shows that they kidneys played a more important role (Figure 15, Table 10). The relatively high accumulation of compounds in liver and kidneys could cause a risk of hepato- or nephrotoxic effects, which seem to be negligible in the case of **15** due to the *in vitro* confirmed lack of influence on the viability of either liver (HepG2) or kidney (HEK-293) cell lines, even at concentrations as high as 50 μM.

In summary, the comprehensive results of ADME *in vivo* in rats, in line with the initial ADMET studies *in vitro*, demonstrated favorable PK properties for **15**, which may be considered a potentially valuable pharmaceutical agent capable of targeting the CNS with a favorable *in vitro* safety profile and satisfactory PK properties after a single i.p. administration.

### *In Vivo* Behavioral Assays for **15**

**Figure 17:**
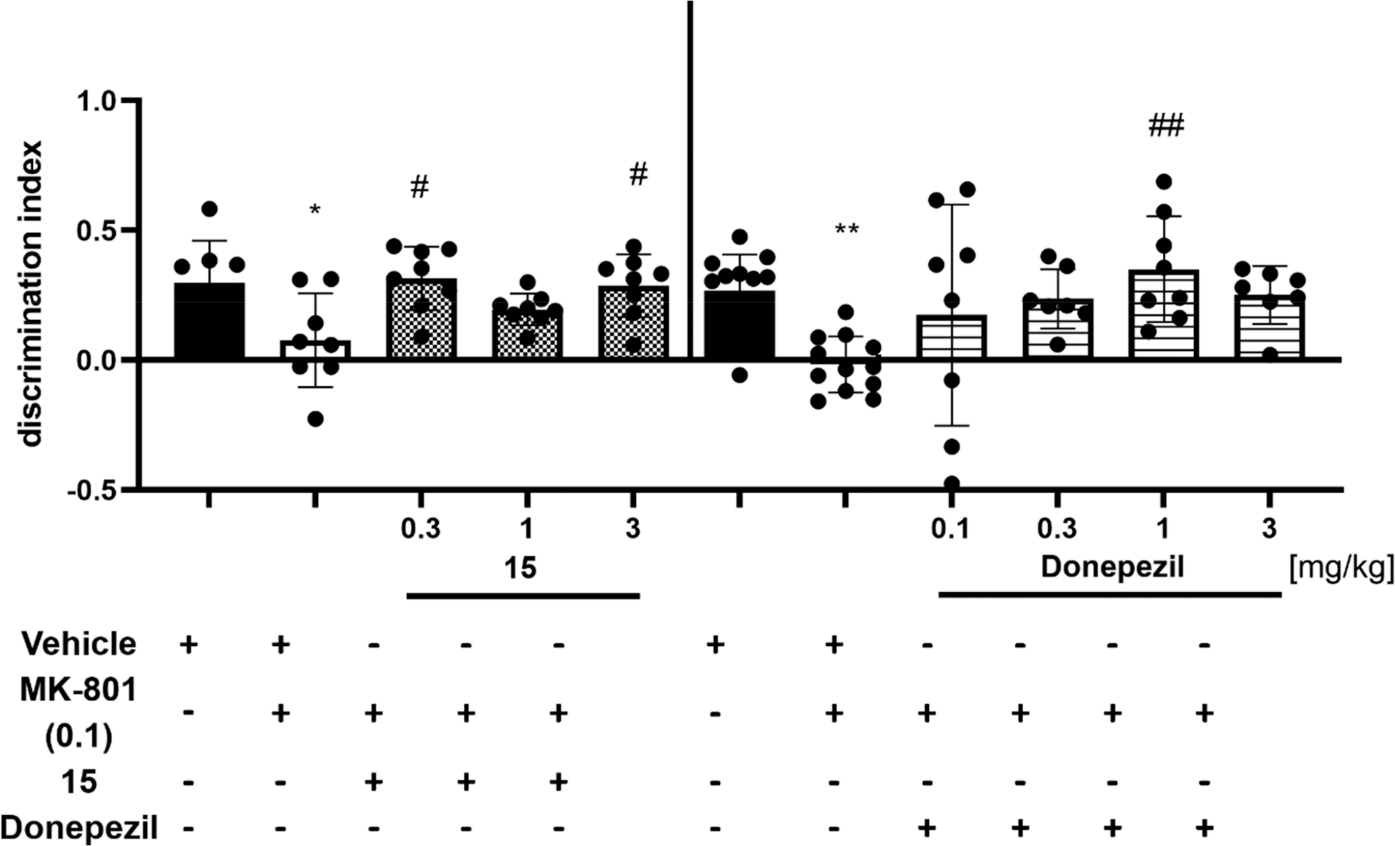
Impact of **15** on the MK-801-induced memory impairment in the NOR test. Donepezil and MK-801 were given i.p. 30 min before while **15** was administered i.p. 60 min before the T1 session. The observation of rats was carried out for 3 min. The data are shown as the mean ± SEM for 8 rats and were statistically evaluated by one-way ANOVA followed by Bonferroni’s post-hoc test. *p < 0.05, ** *p* < 0.01 vs vehicle-treated group; ^#^*p* < 0.05, ^##^*p* < 0.001 vs MK-801 treated group. One-way ANOVA for discrimination index (DI) in NOR test: for **15**, *F*(4,35) = 4.2918, *p* < 0.01; for donepezil, *F*(5,49) = 4.5718, *p* < 0.01.

Based on *in vitro* data obtained, as well as the PK assays *in vivo*, **15** was selected for *in vivo* behavioral studies. First, the ability of **15** to reverse memory impairment was investigated in the novel object recognition (NOR) test. The NOR test was chosen based on our earlier studies with triazine ligands of the 5-HT_6_ receptor.^51^ Thus, we investigated the effect of acute administration of **15** in the NOR test in rats upon MK-801-induced memory impairment. The induction of cognitive deficits in animals is considered a valid model approach to study impairments that occur in humans as, for example, a consequence of developmental intellectual disabilities, aging, or disease processes.^120^ MK-801 (dizocilpine) is an NMDA receptor antagonist capable of inducing cognitive impairments in rodent models related to human cognitive deficits associated with CNS disorders such as dementia^121^ and schizophrenia.^122^ Cognitive deficits induced by MK-801 can be antagonized by putative cognition enhancers with a characteristic pharmacological profile. The 5-HT_6_ receptor ligands were shown to ameliorate these deficits,^34,37^ supporting the predictive validity of animal models with MK-801-induced cognitive deficits. The preference of rats to explore the novel object rather than the familiar object in the T2 session denotes the ability of the investigated compound, given jointly with 0.1 mg/kg of MK-801, to reverse MK-801-induced memory impairment in the NOR test. To give thought to rats’ preference for novel object exploration, the discrimination index (DI) was used (Figure 17). Compound **15**, in a statistically significant manner, reversed MK-801-induced memory impairment, measured by DI level, when injected at doses of 0.3 mg/kg and 3 mg/kg. **15**, administered at a dose of 1 mg/kg, also reversed MK-801-induced memory impairments, but the results of DI were not statistically significant (Figure 17). The reference memory enhancer, donepezil, reversed memory disturbances in the dose range of 0.3–3 mg/kg, but the statistically significant level was reached only for the dose of 1 mg/kg (Figure 17). Thus, the desired pharmacological effect of **15** can be considered more potent than that of donepezil.

In parallel with the evaluation of DI in the T2 phase in the NOR test, the total exploratory time of objects in the recognition phase (T2) was determined after i.p. co-administration of **15** and MK-801 to analyze the impact of the administered compounds on the exploratory activity of rats. **15**, injected with MK-801 (0.1 mg/kg), did not alter the total exploratory activity in T2 (Table 11). Therefore, the observed impact on memory processes in the T2 phase of **15** (Figure 17) appears to be specific, and no disruptive effects related to, e.g., the properties of hyperlocomotor activity of the compound were observed.

**Table 11:** Effects of **15** and Donepezil Administered Jointly with MK-801 on the Exploratory Activity of Rats in the Novel Object Recognition Test^a^

**Treatment**	**Dose (mg/kg)**	**Total exploratory time in T2 (s)**
Vehicle + vehicle	0 + 0	31.88 ± 2.6
Vehicle + MK-801	0 + 0.1	36.63 ± 2.8
**15** + MK-801	0.3 + 0.1	36.25 ± 3.4
	1 + 0.1	33.25 ± 2.7
	3 + 0.1	30.88 ± 3.7
		*F*(4,35) = 0.7015; NS

Vehicle + vehicle	0 + 0	50.31 ± 3.21
vehicle + MK-801	0 + 0.1	51.33 ± 4.86
Donepezil + MK-801	0.1 + 0.1	52.50 ± 3.66
	0.3 + 0.1	42.57 ± 4.06
	1 + 0.1	47.63 ± 5.06
	3 + 0.1	52.14 ± 4.51
		*F*(5,49) = 0.6161; NS

a The data are presented as the mean ± SEM of *N* = 8–14 rats. The data were statistically evaluated by one-way ANOVA followed by Bonferroni’s post-hoc test. NS, not significant.

In the next step, the antidepressant-like properties were assessed for **15**. In the forced swim test (FST), **15** did not show antidepressant-like activity in the whole dose range used. We did not observe a shortening of immobility time after treatment of **15** vs vehicle-treated rats (Table 12).

**Table 12:** Impact of **15** on the Immobility Time in the Forced Swim Test^a^

**Treatment**	**Dose (mg/kg)**	**Immobility time (s)**
**Vehicle**	0	224.00 ± 11.30
**15**	1	244.10 ± 3.40
	3	211.25 ± 13.00
	10	197.57 ± 16.30
		*F*(3,26) = 2.5472; NS

a **15** and vehicle were administered i.p. 60 min before the test. The rats were observed for 5 min. The data are presented as the mean ± SEM of *N* = 6–8 rats. The data were statistically evaluated by one-way ANOVA followed by Bonferroni’s post-hoc test. NS, not significant.

**Figure 18:**
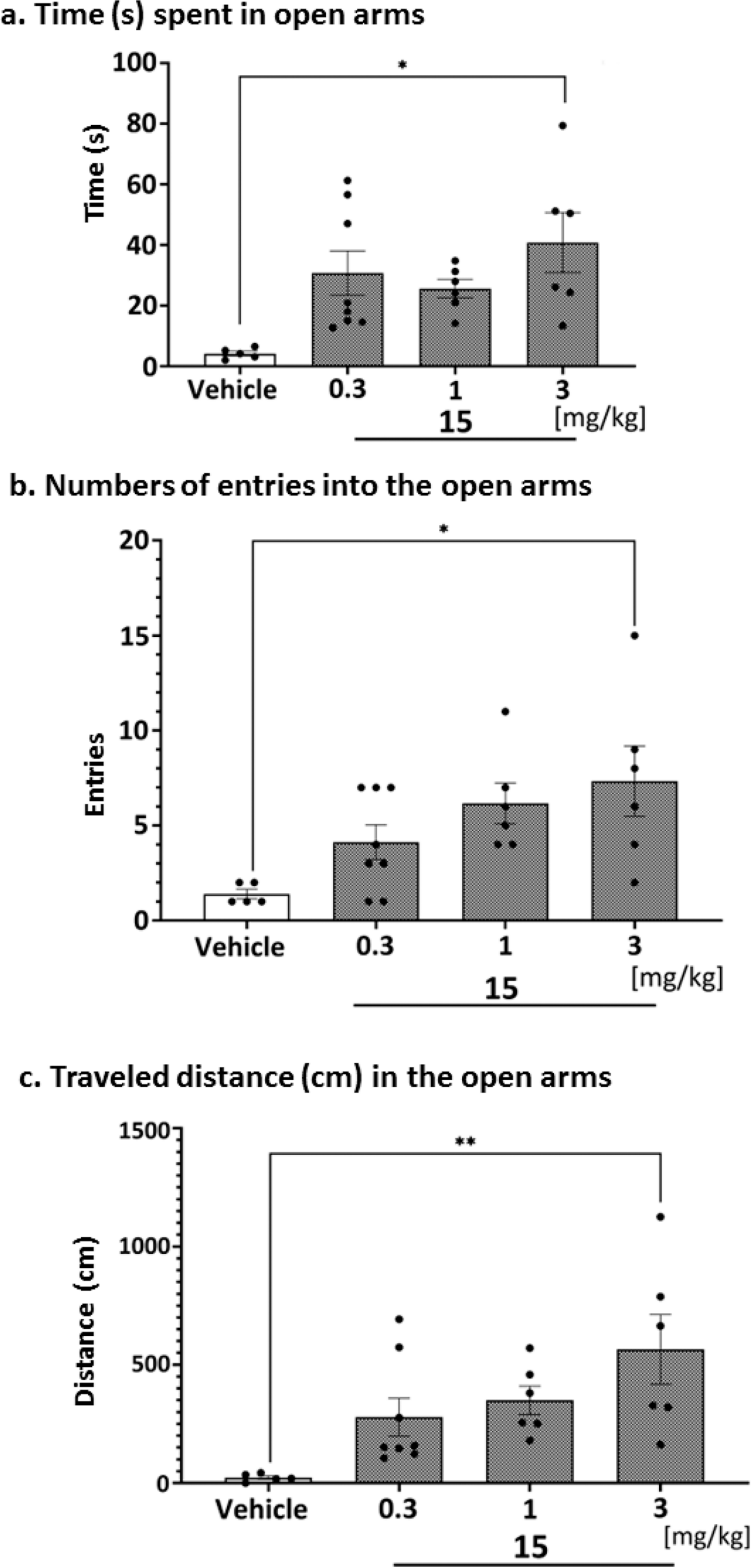
Effect of **15** in the elevated plus-maze (EPM) test. Increased open-arm exploration denotes reduced anxiety. **15** was administered i.p. for 60 min in the test. Values represent the mean ± SEM of the time spent in open arms (a), entries into the open arms (b), and distance covered on the open arms (c) during a 5 min test session compared to the respective vehicle group (one-way ANOVA followed by Bonferroni’s post-hoc test: (a) *F*(3,21) = 4.3870, *p* < 0.05; (b) *F*(3,21) = 4.1764, *p* < 0.05; (c) *F*(3,21) = 4.1764, *p* < 0.05). *p < 0.05, **p < 0.01 vs respective vehicle-treated group; *N* = 6–8.

The potential anxiolytic-like activity of **15** was investigated in the elevated plus-maze (EPM) test. **15** showed anxiolytic-like properties in the entire range of doses used (0.3, 1, and 3 mg/kg), but only the dose of 3 mg/kg of **15** was statistically significant, and the time spent in the open arms and the number of entries were 10 times and 5 times longer vs the vehicle-treated group, respectively (Figure 18). However, we cannot firmly conclude that the observed effect is related to the anxiolytic activity of the dose of 3 mg/kg of **15**, although a statistically significant increase in spontaneous activity was observed, assessed simultaneously with the anxiolytic properties. Administration of **15** at lower doses (0.3 and 1 mg/kg) did not change the total exploratory activity measured simultaneously with the anxiolytic activity (Table 13).

**Table 13:** Effect of **15** on Total Exploration in the Elevated Plus-Maze Test in Rats^a^

**Treatment**	**Dose (mg/kg)**	**Total distance (cm)**	**X Ambulation**	**Y Ambulation**
Vehicle	0	2540 ± 533	91 ± 22	38 ± 9
**15**	0.3	3638 ± 199	134 ± 12	67 ± 6
	1	3975 ± 234;	148 ± 11	70 ± 4
		*p* < 0.05		
	3	3857 ± 207;	149 ± 10	93 ± 10;
		*p* < 0.05		*p* < 0.001
		*F*(3,21) = 4.4094;	*F*(3,21) = 3.4709;	*F*(3,21) = 8.1654;
		*p* < 0.05	*p* < 0.05	*p* < 0.001

a **15** was administered i.p. 60 min before the test. The data are presented as the mean ± SEM of *N* = 6–8 rats for the total distance, X ambulation, and Y ambulation during 5 min test session compared to the respective vehicle group. The data were statistically evaluated by one-way ANOVA followed by Bonferroni’s post-hoc test. NS, not significant.

The results of behavioral studies indicated a significant potency of **15**, more pronounced than that of donepezil, to reverse memory disturbances in the NOR test at a dose as low as 0.3 mg/kg, as well as an anxiolytic-like effect in the EPM test. The procognitive properties *in vivo* are most probably associated with the strong antagonistic action of **15** on 5-HT_6_R, but also with a satisfying PK profile identified for this compound both *in vivo* and *in vitro*. These results, together with the impressive neuroprotective effects demonstrated by **15** in a variety of *in vitro* assays, indicate this β-naphthyl dimethyl-branched selenoether-triazine derivative to be a very promising agent in the search for an innovative therapy for AD that can fight not only the symptoms but also the causes of this severe neurodegenerative disease.

## Conclusions

Despite the plethora of preclinical studies indicating the 5-HT_6_R agents as very promising to become novel therapies for AD, the clinical outcome has yet been disappointing. Considering the complex etiology of this neurodegenerative disease and the high need for novel, innovative therapy regimens, the design of compounds with both multidirectional and unconventional profiles might be the key to a successful approach. In recent years, our research group developed a new family of potent and selective 5-HT_6_R ligands with a 1,3,5-triazine core which, unlike other known ligands, possess neither an indole moiety nor a sulfonyl group in their structures.^40,51,71−73^ The described triazine-based derivatives with oxygen or sulfur atoms in their linkers showed rather moderate 5-HT_6_R potency and considerable selectivity over other serotonin receptors, and the most promising ones reduced MK-801-induced memory impairments in NOR test in rats. In this work, we decided to combine those interesting results with the potential of selenium organic compounds to mimic GPx, which can be highly useful in AD treatment.^75,76^ Although the challenges were high, especially in the area of chemical synthesis, our efforts turned out to be successful. Hence, by exchanging the heteroatom in the linker, we designed and characterized first-in-class selenium-containing 5-HT_6_R agents (**1**–**19**), with particularly highly potent ones (**13**–**15**) compelling neuroprotective properties together with promising pharmacodynamic and pharmacokinetic profiles.

The new compounds **3**, **6**–**15**, and **17** were obtained with a 3–4-step synthesis, in which the most complicated step was to obtain commercially unavailable diselenide compounds of naphthalene or a substituted benzene moiety. After unsuccessful attempts using various literature methods, a sophisticated organometallic chemistry method applying a Grignard reaction, developed and described by us in this work for the first time, was effective.

The whole series **1**–**19** was tested in RBAs, where the compounds presented a wide range of affinities toward 5-HT_6_R, with the most potent compounds substituted with a naphthyl ring reaching *K*_i_ values even below 10 nM. Pharmacological profiles of the most potent 5-HT_6_R agents, **13**–**15**, were compared with those of previously described oxygen- and sulfur-containing agents, proving that the introduction of selenium into the structures of the ligands is a favorable modification. Selenium-containing ligands turned out to be 4–6 times more active than the corresponding oxygen-containing analogs and similarly (for dimethyl branching in the linker) up to 2 times more active (for methyl and ethyl branching in the linker) when compared to corresponding sulfur-containing analogs. The most potent subgroup, **13**–**15**, was also tested for intrinsic activity in the cAMP assay, where they turned out to be antagonists with highly corresponding values of *K*_i_ and *K*_b_. The influence of chalcogen substitution on activity toward 5-HT_6_R was investigated *in silico*. We performed and described, to the best of our knowledge, the first docking and MD studies of seleno compounds to the 5-HT_6_R, upon a challenging parametrization of Se. The obtained results showed that, only in sulfur and selenium derivatives, the amine group attached to triazine is employed to form hydrogen bonds that are responsible for the stronger, compared to the O-analog, 5-HT_6_R affinity observed *in vitro*. Computer-aided SAR analysis also established that the presence of the β-naphthyl ring, as in **15**, among tested aromatic moieties is the most beneficial for activity toward 5-HT_6_R, very likely due to the stronger stabilization via π–π interactions with the aromatic amino acids abundant in the 5-HT_6_R binding pocket.

Due to excellent results in the pharmacological screening, **13**–**15** were further investigated for neuroprotective properties in comprehensive *in vitro* studies. All three compounds protected cells from oxidative stress in the neuroblastoma SH-SY5Y model. They also presented a total antioxidant capacity similar to that of ascorbic acid at low concentrations, most potent in the case of **15** at concentrations close to pharmacologically active doses. Further mechanistic studies underlying antioxidant properties of the compounds demonstrated that **13**–**15** regulate antioxidant and pro-inflammatory genes such as NRF2, HO-1, SOD1, and NQO1 and that their effect is even more evident with respect to donepezil. Especially the dimethyl-branched **15** occurred as an inducer of NRF2 nuclear translocation, which may lead to an increased expression of antioxidant genes. These results further confirm that the introduction of selenium into the structure of the 5-HT_6_R triazine ligands broadens their mode of action by involving numerous mechanisms of antioxidant effects. This is especially beneficial in the context of searching for innovative therapies for AD, where oxidative stress plays an essential role in the development of the disease. The introduction of selenium into our 1,3,5-triazine-based serotonin-receptor ligands and subsequent optimization toward selective 5-HT_6_R resulted not only in modulating a single target (i.e., 5-HT_6_R) but also in mimicking GPx, which can be highly useful in AD treatment.^75,76^ This combination approach provides a very promising strategy, not reported before, meeting the polypharmacology criteria, which seem necessary to deal with neurodegenerative diseases involving several pathophysiological processes.^123^

In order to determine the drug-likeness of **13**–**15***in vitro*, an assessment of PK properties was performed. All three compounds presented favorable permeability in the PAMPA and satisfying metabolic stability in rLMs (**13**, **14**) and hLMs (**15**) models. **15**, as hit structure in this study, was tested for its affinity toward a broader panel of CNS targets, showing minimal impact (0–15% at 1 μM) on histamine H_3_, muscarinic M_1_, cannabinoid CB_1_, α_2_ adrenergic, and NMDA receptors, indicating a significant selectivity for 5-HT_6_R. As part of broader safety considerations, **15** was examined *in vitro* for hERG inhibition, hepatotoxicity (HepG2), nephrotoxicity (HEK-293), and mutagenic effects (Ames test). It exhibited moderate hERG inhibition, 3 times weaker than that of verapamil, as well as no cytotoxicity on HepG2 and HEK-293 cell lines. Finally, **15** demonstrated safety in an Ames test with *S. typhimurium* TA98, even in the presence of rat liver S9 fraction, suggesting it has a low risk of mutagenicity.

The subsequent *in vivo* data showed that **15** was rapidly absorbed after i.p. administration and extensively distributed to tissues, including brain. Thus, **15** is able to cross the BBB, which is vital for therapeutic effects *in vivo* in AD. Considering the promising ADMET data *in vitro* and *in vivo*, **15** was administered in rats for behavioral studies *in vivo*. The NOR, EPM, and FST tests indicate its potent procognitive-like action that was able to significantly hamper the effects of memory impairment induced by MK-801 at a dose of **15** (0.3 mg/kg) even lower than that of the donepezil used as control, as well as anxiolytic-like properties (3 mg/kg).

In summary, the β-naphthyl dimethyl-branched selenoether-triazine derivative **15** is a first-in-class selenium-containing, highly potent 5-HT_6_R antagonist with very good ADMET properties and confirmed BBB penetration *in vivo*, exhibiting a comprehensive neuroprotection profile combined with a procognitive activity in rats. The overall favorable data further suggest more advanced and broader clinical studies for **15**, which possesses considerable potential to become a clinical candidate, possibly bringing novelty and effectiveness to AD therapy.

## Experimental Section

### Chemistry

^1^H NMR and ^13^C NMR spectra were routinely recorded in DMSO-*d*_6_ at 500 and 126 MHz, respectively, in the basic form of final compounds (except **9**, **10**, and **15**, for which spectra were recorded for hydrochlorides) on an FT-NMR 500 MHz JEOL (JNM-ECZR500 RS1 version ECZR) apparatus. ^77^Se NMR spectra were recorded in DMSO-*d*_6_ at 95 MHz on an FT-NMR 400 MHz Bruker Avance spectrometer. Spectroscopy was carried out at ambient temperature using the solvent signal as an internal standard. Chemical shifts in spectra were reported in parts per million (ppm) on the δ scale with coupling constants (*J*) values in Hertz. The UPLC-MS/MS system consisted of a Waters Acquity Premier (Waters Corp., Milford, MA, USA) coupled with a Waters Xevo TQ-S Cronos mass spectrometer (electrospray ionization (ESI) mode). Chromatographic separations were carried out using an Acquity UPLC BEH (bridged ethylene hybrid) C18 column, 2.1 × 100 mm, and 1.7 μm particle size, equipped an Acquity UPLC BEH C18 VanGuard precolumn, 2.1 × 5 mm, and 1.7 μm particle size. The column was maintained at 40 °C and eluted under gradient conditions using from 95% to 0% of eluent A over 10 min, at a flow rate of 0.3 mL min^–1^. Eluent A: water/formic acid (0.1%, v/v); eluent B: acetonitrile/formic acid (0.1%, v/v). Chromatograms were recorded using Waters eλ PDA detector. Spectra were analyzed in the 200–500 nm range with 1.2 nm resolution and a sampling rate of 20 points/s. MS detection settings of Waters Xevo TQ-S Cronos mass spectrometer were as follows: source temperature 150 °C, desolvation temperature 350 °C, desolvation gas flow rate 600 L h^–1^, cone gas flow 100 L h^–1^, capillary potential 3.00 kV, cone potential 30 V. Nitrogen was used for both nebulizing and drying gas. The data were obtained in a scan mode ranging from 50 to 1000 *m*/*z* in 0.5 s intervals. The data acquisition software was MassLynx V 4.2 (Waters). Melting points were determined on a Buchi 530 melting point apparatus and are uncorrected. Elemental analysis has been performed for **3**, **6**–**15**, and **17** on a ThermoFisher FlashSmart CNHS/O apparatus (for data see the Supporting Information). Thin-layer chromatography (TLC) was performed on Merck silica gel 60 F254 plates, and the spots were visualized by UV light. All chemicals were purchased from Merck (Darmstadt, Germany), AlfaAesar/ThermoFisher (Schwerte, Germany), or Sigma-Aldrich (Schellendorf, Germany), as well as the analytical-grade solvents. The final compounds **3**, **6**–**15**, and **17** possess a purity >95%, confirmed by ^1^H and ^13^C NMR, as well as elemental analysis and HPLC.

### General Procedure for the Synthesis of Diselenides **20**–**25** with Grignard Reaction

Magnesium (56.5 mmol) was added to a dry flask under inert conditions, then 30 mL of anhydrous THF and 1–2 tiny crystals of iodine were added. A suitable halobenzene (54 mmol) was dissolved in 30 mL of anhydrous THF and added dropwise to the reaction flask; then, the reaction mixture was heated to reflux. After refluxing for 2–5 min, 325-mesh selenium (54 mmol) was slowly added, and the reaction mixture was stirred at room temperature for 1 h under TLC control (petroleum ether:ethyl acetate 9:1). Subsequently, ethanol (15 mL) and 20% ammonium chloride solution (15 mL) were added dropwise, respectively, and the reaction mixture was stirred in open atmosphere for 2 h. The mixture was extracted with DCM (4 × 90 mL). Organic fractions were combined, washed with water, dried over Na_2_SO_4_, and evaporated under vacuum. The desirable product was obtained as a mixture of organic selenium derivatives and used in the crude form for further synthesis without additional purification.

The selenoether ester intermediates **26**–**30**, **41**, **43**, and **44** were prepared as described previously.^93,94,124^

### General Procedure for the Synthesis of Selenoether Ester Intermediates **31**–**40**, **42**

An appropriate diaryldiselenide (9 mmol) was dissolved in a 1:1 mixture of water and THF (50 mL) under nitrogen gas. NaBH_4_ (45 mmol) was added. The reaction mixture was stirred at room temperature for around 30 min, and then a solution of suitable bromoester (18 mmol) in THF (5 mL) was added without opening the reaction apparatus. The reaction mixture was stirred at room temperature and monitored via TLC (24–48 h). Subsequently, the solution was stirred for further 30 min on air, and then the reaction mixture was diluted with 50 mL of a saturated aqueous solution of NH_4_Cl and extracted with dichloromethane. The combined organic phases were dried and filtered, and the solvent was evaporated under reduced pressure to get the product, which was finally purified using flash chromatography.

#### Methyl 2-((2,5-dimethylphenyl)selanyl)propanoate **31**

Yellowish oil, yield 55.3%. ^1^H NMR (500 MHz, DMSO-*d*_6_) δ 7.35 (s, 1H), 7.14 (d, *J* = 7.7 Hz, 1H), 7.04 (d, *J* = 7.4 Hz, 1H), 3.88 (q, *J* = 7.0 Hz, 1H), 3.50 (s, 3H), 2.29 (s, 3H), 2.22 (s, 3H), 1.40 (d, *J* = 6.9 Hz, 3H) ppm.

#### Methyl 2-((2,5-dimethylphenyl)selanyl)butanoate (**32**)

Yellowish oil, yield 49.7%. ^1^H NMR (500 MHz, DMSO-*d*_6_) δ 7.34 (s, 1H), 7.13 (d, *J* = 7.4 Hz, 1H), 7.03 (d, *J* = 7.4 Hz, 1H), 3.65 (dd, *J* = 8.6, 6.6 Hz, 1H), 3.50 (s, 3H), 2.29 (s, 3H), 2.22 (s, 3H), 1.80 (q, *J* = 7.1 Hz, 1H), 1.69 (q, *J* = 6.8 Hz, 1H), 0.89 (t, *J* = 7.3 Hz, 3H) ppm.

#### Ethyl 2-((2,5-dimethylphenyl)selanyl)pentanoate (**33**)

Yellowish oil, yield 44.3%. ^1^H NMR (500 MHz, DMSO-*d*_6_) δ 7.34 (dt, *J* = 7.2, 3.4 Hz, 1H), 7.11 (dd, *J* = 11.1, 4.0 Hz, 1H), 7.04–7.01 (m, 1H), 3.97–3.87 (m, 2H), 3.71–3.62 (m, 1H), 2.29 (s, 3H), 2.20 (s, 3H), 1.82–1.71 (m, 1H), 1.68–1.54 (m, 1H), 1.41–1.10 (m, 2H), 1.00 (dd, *J* = 8.0, 6.2 Hz, 3H), 0.82 (t, *J* = 7.4 Hz, 3H) ppm.

#### Methyl 2-((2,5-dimethylphenyl)selanyl)-2-methylpropanoate (**34**)

Yellowish oil, yield 61.2%. ^1^H NMR (500 MHz, DMSO-*d*_6_) δ 7.28 (dd, *J* = 2.9, 1.6 Hz, 1H), 7.18 (t, *J* = 5.1 Hz, 1H), 7.12–7.09 (m, 1H), 3.50 (s, 3H), 2.31 (s, 3H), 2.21 (s, 3H), 1.48–1.42 (s, 6H) ppm.

#### Ethyl 2-((5-chloro-2-fluorophenyl)selanyl)pentanoate (**35**)

Yellowish oil, yield 55.6%. ^1^H NMR (500 MHz, DMSO-*d*_6_) δ 7.57 (dd, *J* = 5.7, 2.6 Hz, 1H), 7.27 (ddd, *J* = 8.7, 4.4, 2.6 Hz, 1H), 7.02 (t, 1H), 4.08 (qd, *J* = 7.1, 2.0 Hz, 2H), 3.72 (dd, *J* = 8.9, 6.4 Hz, 1H), 1.95–1.70 (m, 2H), 1.48–1.37 (m, 2H), 1.15 (t, *J* = 7.1 Hz, 3H), 0.92 (t, *J* = 7.4 Hz, 3H) ppm.

#### Methyl 2-(naphthalen-1-ylselanyl)propanoate (**36**)

Yellowish oil, yield 48.3%. ^1^H NMR (500 MHz, DMSO-*d*_6_) δ 8.30 (d, *J* = 8.6 Hz, 1H), 7.95 (d, *J* = 8.0 Hz, 1H), 7.92 (d, *J* = 8.0 Hz, 1H), 7.85 (dd, *J* = 7.2, 0.9 Hz, 1H), 7.60–7.56 (m, 1H), 7.55–7.49 (m, 1H), 7.43 (dd, *J* = 8.0, 7.4 Hz, 1H), 3.91 (q, *J* = 6.9 Hz, 1H), 3.37 (s, 3H), 1.38 (d, *J* = 6.9 Hz, 3H) ppm.

#### Methyl 2-(naphthalen-1-ylselanyl)butanoate (**37**)

Yellowish oil, yield 37.7%. ^1^H NMR (500 MHz, DMSO-*d*_6_) δ 8.29 (d, *J* = 8.6 Hz, 1H), 7.96 (d, *J* = 8.2 Hz, 1H), 7.92 (d, *J* = 8.2 Hz, 1H), 7.85 (d, *J* = 7.2, Hz, 1H), 7.62–7.58 (m, 1H), 7.56–7.51 (m, 1H), 7.46–7.41 (m, 1H), 3.70 (dd, *J* = 8.6, 6.6 Hz, 1H), 3.37 (s, 3H), 1.80–1.68 (m, 2H), 0.88 (t, *J* = 7.3 Hz, 3H) ppm.

#### Methyl 2-(naphthalen-2-ylselanyl)propanoate (**38**)

Yellowish oil, yield 22.0%. ^1^H NMR (500 MHz, DMSO-*d*_6_) δ 8.11 (s, 1H), 7.92–7.86 (m, 2H), 7.85 (d, *J* = 8.6, Hz, 1H), 7.56 (dd, *J* = 8.4, 1,6 Hz, 1H), 7.52–7.47 (m, 2H), 3.92 (q, *J* = 6.9 Hz, 1H), 3.37 (s, 3H), 1.35 (d, *J* = 6.9 Hz, 3H) ppm.

#### Methyl 2-(naphthalen-2-ylselanyl)butanoate (**39**)

Yellowish oil, yield 43.9%. ^1^H NMR (500 MHz, DMSO-*d*_6_) δ 8.12 (s, 1H), 7.91–7.85 (m, 2H), 7.83 (d, *J* = 8.6, Hz, 1H), 7.58 (dd, *J* = 8.4, 1,6 Hz, 1H), 7.53–7.48 (m, 2H), 3.81 (dd, *J* = 8.3, 6.6 Hz, 1H), 3.53 (s, 3H), 1.82–1.67 (m, 2H), 1.70 (dt, *J* = 14.0, 7.0 Hz, 1H), 0.90 (t, *J* = 7.3 Hz, 3H) ppm.

#### Methyl 2-methyl-2-(naphthalen-2-ylselanyl)propanoate (**40**)

Yellowish oil, yield 53.2%. ^1^H NMR (500 MHz, DMSO-*d*_6_) δ 8.11 (s, 1H), 7.84–7.80 (m, 2H), 7.76 (d, *J* = 8.4 Hz, 1H), 7.61 (d, *J* = 8.4 Hz, 1H), 7.52–7.49 (m, 2H), 3.62 (s, 3H), 1.60 (s, 6H) ppm.

#### Methyl 2-(benzylselanyl)propanoate (**42**)

Yellowish oil, yield 85.0%. ^1^H NMR (500 MHz, DMSO-*d*_6_) δ 7.32 (d, *J* = 7.1 Hz, 4H), 7.26–7.19 (m, 1H), 3.98 (s, 2H), 3.64 (s, 3H), 3.54 (q, *J* = 7.1 Hz, 1H), 1.42 (d, *J* = 7.1 Hz, 3H) ppm.

### General Procedure for the Synthesis of Selenoether Derivatives of 1,3,5-Triazine **1**–**19**

All final compounds **1**–**19** were prepared as described in ref (51). Briefly, sodium (8 mmol) was dissolved in 10 mL of absolute methanol, and then the (4-methyl-1-piperazinyl)biguanide dihydrochloride (3 mmol) and a suitable carboxylic acid ester-containing Se-ether (3–5 mmol) were added. The reaction mixture was refluxed for 15–30 h. After cooling to room temperature, the solvent was evaporated, and the residue was dissolved in water (10 mL), stirred for 30 min at room temperature, and kept overnight in a refrigerator. The precipitated triazine product was isolated by filtration and crystallized from methanol to give the desired final products as solids in basic form (method A). In case of a lack of desirable precipitate, the product was extracted with dichloromethane and purified by flash chromatography (method B).

#### 4-(4-Methylpiperazin-1-yl)-6-(1-(phenylselanyl)propyl)-1,3,5-triazin-2-amine (**1**)

Prepared according to Ali et al.^93^

#### 4-(4-Methylpiperazin-1-yl)-6-(1-(phenylselanyl)butyl)-1,3,5-triazin-2-amine (**2**)

Prepared according to Ali et al.^94^

#### 4-(4-Methylpiperazin-1-yl)-6-(1-(phenylselanyl)pentyl)-1,3,5-triazin-2-amine (**3**)

Method B, white solid, yield 18.5%, mp 278–279 °C (hydrochloride). ^1^H NMR (500 MHz, DMSO*-d*_6_) δ 11.76 (s, 1H), 7.54 (m, 2H) 7.41–7.32 (m, 3H), 4.54–4.37 (m, 2H), 4.11 (t, *J* = 7.5 Hz, 8.5 Hz, 1H), 3.47–3.44 (m, 3H), 3.07 (m, 3H), 2.75 (s, 3H), 2.02 (s, 1H), 1.83 (s, 1H), 1.41–1.23 (m, 6H), 0.82 (t, *J* = 7.0 Hz, 3H) ppm. ^13^C NMR (126 MHz, DMSO-*d*_6_) δ 161.76, 135.32, 129.16, 128.66, 51.30, 41.91, 40.11, 39.93, 30.92, 29.38, 21.69, 21.60, 13.76, 13.71 ppm. ^77^Se NMR (95 MHz, DMSO*-d*_6_) δ 460.66 ppm. MS (ESI) *m*/*z* calcd for C_19_H_28_N_6_Se [M+H]^+^ 421.16, found [M+H]^+^ 421.27.

#### 4-(4-Methylpiperazin-1-yl)-6-(2-(phenylselanyl)propan-2-yl)-1,3,5-triazin-2-amine (**4**)

Prepared according to Ali et al.^93^

#### 4-(4-Methylpiperazin-1-yl)-6-(3-(phenylselanyl)propyl)-1,3,5-triazin-2-amine (**5**)

Prepared according to Ali et al.^94^

#### 4-(1-((2,5-Dimethylphenyl)selanyl)ethyl)-6-(4-methylpiperazin-1-yl)-1,3,5-triazin-2-amine (**6**)

Method B, yellowish solid, yield 28.4%, mp 107 °C (free base). ^1^H NMR (500 MHz, DMSO-*d*_6_) δ 7.38 (s, 1H), 7.06 (d, *J* = 7.5 Hz, 1H), 6.95 (d, *J* = 6.0 Hz, 1H), 6.85–6.7668 (m, 2H), 4.02 (q, *J* = 7.0 Hz, 3.59 (br s, 4H), 2.24–2.20 (m, 10H), 2.14 (s, 3H), 1.57 (q, *J* = 6.0 Hz, 7.0 Hz, 6.0 Hz, 3H) ppm. ^13^C NMR (126 MHz, DMSO-*d*_6_) δ 178.56, 167.53, 164.86, 136.79, 135.99, 134.54, 131.23, 130.15, 128.63, 55.46, 55.34, 46.31, 43.40, 42.87, 31.23, 22.19, 20.93, 20.24 ppm. ^77^Se NMR (95 MHz, DMSO- *d*_6_) δ 398.07 ppm. MS (ESI) *m*/*z* calcd for C_18_H_26_N_6_Se [M+H]^+^ 407.15, found [M+H]^+^ 406.95.

#### 4-(1-((2,5-Dimethylphenyl)selanyl)propyl)-6-(4-methylpiperazin-1-yl)-1,3,5-triazin-2-amine (**7**)

Method A, yellowish solid, yield 33.3%, mp 285 °C (hydrochloride). ^1^H NMR (500 MHz, DMSO-*d*_6_) δ 7.37 (s, 1H), 7.04 (d, *J* = 8.0 Hz, 1H), 6.93 (d, *J* = 7.75 Hz, 1H), 6.84–6.75 (m, 2H), 3.76 (dd, *J* = 15.0 Hz, 1H), 3.67–3.57 (m, 4H), 3.13 (s, 1H), 2.24–2.20 (m, 8H), 2.14 (s, 3H), 2.11–2.02 (m, 1H), 1.88–1.80 (m, 1H), 0.84 (t, *J* = 7.0 Hz, 7.5 Hz, 10.0 Hz, 3H) ppm. ^13^C NMR (126 MHz, DMSO-*d*_6_) δ 177.82, 167.53, 164.87, 136.67, 135.95, 134.38, 131.40, 130.13, 128.53, 54.83, 51.03, 49.13, 46.31, 42.87, 27.22, 22.18, 20.94, 13.32, 12.97 ppm. ^77^Se NMR (95 MHz, DMSO- *d*_6_) δ 402.05 ppm. MS (ESI) *m*/*z* calcd for C_19_H_28_N_6_Se [M+H]^+^ 421.16, found [M+H]^+^ 421.21.

#### 4-(1-((2,5-Dimethylphenyl)selanyl)butyl)-6-(4-methylpiperazin-1-yl)-1,3,5-triazin-2-amine (**8**)

Method B, white solid, yield 7.6%, mp 143–145 °C (free base). ^1^H NMR (500 MHz, DMSO-*d*_6_) δ 7.34 (s, 1H), 7.03 (d, *J* = 7.5 Hz, 1H), 6.92 (d, *J* = 8.5 Hz, 1H), 6.79–6.73 (m, 2H), 3.82 (dd, *J* = 15.0 Hz, 1H), 3.57–3.48 (m, 4H), 2.22–2.18 (m, 10H),, 2.13 (s, 3H), 2.06–1.99 (m, 1H), 1.81–1.74 (m, 1H), 1.32–1.18 (m, 2H), 0.79 (t, *J* = 7.5 Hz, 7.0 Hz, 3H) ppm. ^13^C NMR (126 MHz, DMSO d6) δ 178.02, 167.50, 164.83, 136.66, 135.95, 134.32, 131.34, 130.15, 128.57, 54.78, 49.04, 46.26, 42.83, 36.04, 22.15, 21.45, 20.93, 14.14 ppm. ^77^Se NMR (95 MHz, DMSO- *d*_6_) δ 378.39 ppm. MS (ESI) *m*/*z* calcd for C_20_H_30_N_6_Se [M]^+^ 434.17 found [M]^+^ 434.96.

#### 4-(2-((2,5-Dimethylphenyl)selanyl)propan-2-yl)-6-(4-methylpiperazin-1-yl)-1,3,5-triazin-2-amine (**9**)

Method B, white solid, yield 5.5%, mp 265–267 °C (hydrochloride). ^1^H NMR (500 MHz, D_2_O) δ 7.15 (d, *J* = 8.5 Hz, 1H), 7.10 (d, *J* = 5.75 Hz, 2H), 3.50 (dd, *J* = 21.5 Hz, 1H), 3.20 (s, 1H), 3.05 (s, 1H), 2.84–2.83 (m, 1H), 2.81 (s, 4H), 2.76 (m, 1H), 2.17 (s, 3H), 2.10 (s, 3H), 1.68 (s, 7H), 1.03 (t, *J* = 7.0 Hz, 1H) ppm. ^13^C NMR (126 MHz, DMSO-*d*_6_) δ 139.69, 139.40, 135.26, 130.49, 129.65, 128.41, 71.14, 51.53, 48.56, 41.95, 39.84, 39.02, 26.42, 22.38, 20.18, 18.37 ppm. ^77^Se NMR (95 MHz, DMSO- *d*_6_) δ 402.09 ppm. MS (ESI) *m*/*z* calcd for C_19_H_28_N_6_Se [M]^+^ 420.15, found [M]^+^ 420.84.

#### 4-(1-((5-Chloro-2-fluorophenyl)selanyl)butyl)-6-(4-methylpiperazin-1-yl)-1,3,5-triazin-2-amine (**10**)

Method B, yellowish solid, yield 36.1%, mp 122–124 °C (free base). ^1^H NMR (500 MHz, DMSO-*d*_6_) δ 11.43 (s, 1H), 7.68 (dd, *J* = 8.5 Hz, 1H), 7.45 (m, 1H), 7.30 (t, *J* = 8.5 Hz, 1H), 4.52–4.44 (d, *J* = 38.0 Hz, 2H), 4.17 (t, *J* = 7.75 Hz, 7.25 Hz, 1H), 3.43–3.32 (m, 3H), 2.99 (m, 2H), 2.71 (s, 3H), 2.02–1.95 (m, 1H), 1.84–1.77 (m, 1H), 1.40–1.27 (m, 2H), 0.85 (t, *J* = 7.0 Hz, 7.5 Hz, 5 Hz, 3H) ppm. ^13^C NMR (126 MHz, DMSO-*d*_6_) δ 164.61, 161.20, 159.29, 134.42, 133.51,129.14, 129.11, 117.61, 117.41, 55.47, 52.12, 49.11, 42.52, 35.38, 21.19, 14.20, 14.15, 14.10 ppm. ^77^Se NMR (95 MHz, DMSO- *d*_6_) δ 361.23 ppm. MS (ESI) *m*/*z* calcd for C_18_H_24_ClFN_6_Se [M+H]^+^ 459.10, found [M+H]^+^ 459.22.

#### 4-(4-Methylpiperazin-1-yl)-6-(1-(naphthalen-1-ylselanyl)ethyl)-1,3,5-triazin-2-amine (**11**)

Method B, yellowish solid, yield 33.2%, mp 221–224 °C (hydrochloride). ^1^H NMR (500 MHz, DMSO-*d*_6_) δ 8.29–8.19 (m, 1H), 7.91–7.85 (m, 3H), 7.51–7.47 (m, 2H), 7.40 (t, *J* = 15.5 Hz, 1H), 6.85–6.75 (m, 2H), 4.06 (q, *J* = 6.5 Hz, 7.5 Hz, 7.0 Hz, 1H), 3.54–3.34 (m, 4H), 3.13 (s, 1H), 2.25–2.14 (m, 3H), 2.12 (s, 3H), 2.08–2.07 (m, 1H), 1.54 (d, *J* = 7.0 Hz, 3H) ppm. ^13^C NMR (126 MHz, DMSO-*d*_6_) δ 161.24, 137.86, 137.83, 135.57, 133.99, 131.02, 129.35, 127.57, 127.55, 126.93, 126.91, 126.47, 126.06, 51.67, 42.37, 42.33, 40.43, 38.87, 38.23, 17.55 ppm. ^77^Se NMR (95 MHz, DMSO-*d*_6_) δ 383.19 ppm. MS (ESI) *m*/*z* calcd for C_20_H_24_N_6_Se [M+H]^+^ 429.13, found [M+H]^+^ 429.20.

#### 4-(4-Methylpiperazin-1-yl)-6-(1-(naphthalen-1-ylselanyl)propyl)-1,3,5-triazin-2-amine (**12**)

Method B, yellowish solid, yield 24.0%, mp 245–247 °C (hydrochloride). ^1^H NMR (500 MHz, DMSO-*d*_6_) δ 8.22–8.20 (m, 1H), 7.90–7.84 (m, 3H), 7.51–7.47 (m, 2H), 7.38 (t, *J* = 15.5 Hz, 1H), 6.85–6.74 (m, 2H), 3.80 (dd, *J* = 15.0 Hz, 1H), 3.55 (m, 1H), 3.54–3.50 (m, 1H), 3.41–3.33 (m, 2H), 2.25–2.13 (m, 3H), 2.12 (s, 3H), 2.12–2.00 (m, 2H), 1.90–1.81 (m, 1H), 0.81 (t, *J* = 7.5 Hz, 7.0 Hz, 3H) ppm. ^13^C NMR (126 MHz, DMSO-*d*_*6*_) δ 177.35, 167.50, 164.71, 134.96, 134.78, 134.08, 129.41, 129.36, 129.12, 127.88, 127.21, 126.73, 126.40, 55.46, 54.70, 52.04, 51.44, 46.28,42.73, 27.01, 13.22 ppm. ^77^Se NMR (95 MHz, DMSO- *d*_6_) δ 381.26 ppm. MS (ESI) *m*/*z* calcd for C_21_H_26_N_6_Se [M+H]^+^ 443.15, found [M+H]^+^443.14.

#### 4-(4-Methylpiperazin-1-yl)-6-(1-(naphthalen-2-ylselanyl)ethyl)-1,3,5-triazin-2-amine (**13**)

Method B, yellowish solid, yield 31.2%, mp 214–215 °C (hydrochloride). ^1^H NMR (500 MHz, DMSO-*d*_6_) δ 8.07 (s, 1H), 7.86–7.81 (m, 2H), 7.78 (d, *J* = 9.0 Hz, 1H), 7.58 (dd, *J* = 10.0, 1H), 7.50–7.45 (m, 2H), 6.88–6.78 (m, 2H), 4.20 (q, *J* = 7.0 Hz, 7.0 Hz, 7.5 Hz, 1H), 3.57–3.51 (m, 6H), 2.15–2.11 (m, 2H), 2.09 (s, 3H), 1.62 (q, *J* = 6.0 Hz, 7.0 Hz, 5.5 Hz, 3H) ppm. ^13^C NMR (126 MHz, DMSO-*d*_6_) δ 162.15, 135.09, 133.77, 132.92, 128.83, 128.22, 128.13,127.51, 127.30, 125.52, 51.73, 42.43, 40.63, 40.46, 40.29, 40.12, 18.36 ppm. ^77^Se NMR (95 MHz, DMSO- *d*_6_) δ 458.26 ppm. MS (ESI) *m*/*z* calcd for C_20_H_24_N_6_Se [M+H]^+^ 429.13, found [M+H]^+^ 429.27.

#### 4-(4-Methylpiperazin-1-yl)-6-(1-(naphthalen-2-ylselanyl)propyl)-1,3,5-triazin-2-amine (**14**)

Method B, yellowish solid, yield 43.9%, 203–205 °C (hydrochloride). ^1^H NMR (500 MHz, DMSO-*d*_6_) δ 8.06 (s, 1H), 7.86–7.80 (m, 2H), 7.77 (d, *J* = 8.5 Hz, 1H), 7.57 (dd, *J* = 10.5 Hz, 1H), 7.50–7.44 (m, 2H), 6.87–6.77 (m, 2H), 3.93 (dd, *J* = 15.0 Hz, 1H), 3.55–3.43 (m, 4H), 2.21–2.09 (m, 3H), 2.08 (s, 3H), 2.07–2.01 (m, 2H), 1.94–1.85 (m, 1H), 0.87 (t, *J* = 7.5 Hz, 7.0 Hz, 3H) ppm. ^13^C NMR (126 MHz, DMSO-*d*_6_) δ 177.70, 167.53, 164.78, 133.97, 132.46, 132.41, 131.37 128.63, 128.16, 127.80, 127.05, 126.74, 55.46, 54.70, 51.61, 46.25, 42.81, 40.62,27.08, 13.24 ppm. ^77^Se NMR (95 MHz, DMSO- *d*_6_) δ 458.29 ppm. MS (ESI) *m*/*z* calcd for C_21_H_26_N_6_Se [M+H]^+^ 443.15, found [M+H]^+^ 443.23.

#### 4-(4-methylpiperazin-1-yl)-6-((2-(naphthalen-2-yl)propan-2-yl)selanyl)-1,3,5-triazin-2-amine (**15**)

Method B, yellowish solid, yield 18.3%, mp 244–245 °C (hydrochloride). ^1^H NMR (500 MHz, DMSO-*d*_6_) δ 7.98 (s, 1H), 7.89–7.88 (m, 1H), 7.85–7.83 (m, 1H), 7.77 (d, *J* = 8.5 Hz, 1H), 7.53–7.47 (m, 2H), 7.48 (dd, *J* = 10.0 Hz, 1H), 6.75 (s, 2H), 3.54 (s, 2H) 3.30 (s, 4H), 2.20 (t, J = 5.5 Hz, 5.0 Hz, 1H), 2.13 (s, 1H), 2.06 (s, 3H), 1.63 (s, 6H) ppm. ^13^C NMR (126 MHz, DMSO-*d*_6_) δ 138.04, 134.44, 133.57, 133.30, 128.66, 128.47, 128.21, 128.02, 127.33, 51.74, 42.42, 40.47, 40.30, 40.14 ppm. ^77^Se NMR (95 MHz, DMSO- *d*_6_) δ 458.23 ppm. MS (ESI) *m*/*z* calcd for C_21_H_26_N_6_Se [M+H]^+^ 443.15, found [M+H]^+^ 443.27.

#### 4-((benzylselanyl)methyl)-6-(4-methylpiperazin-1-yl)-1,3,5-triazin-2-amine (**16**)

Prepared according to Ali et al.^94^

#### 4-(1-(benzylselanyl)ethyl)-6-(4-methylpiperazin-1-yl)-1,3,5-triazin-2-amine (**17**)

Method B, red solid, yield 13.5%, mp 154–155 °C (free base). ^1^H NMR (500 MHz, DMSO-*d*_6_) δ 11.81 (s, 1H), 8.53–7.87 (m, 1H), 7.36–7.34 (d, *J* = 7.5 Hz, 2H), 7.30–7.19 (m, 3H), 4.75–4.58 (m, 2H), 4.10 (q, *J* = 12.0 Hz, 17.0 Hz, 12.0 Hz, 2H) 3.97 (q, *J* = 7.0 Hz, 2H), 3.52–3.49 (m, 4H), 3.16 (s, 2H), 2.75 (s, 3H), 1.61 (q, *J* = 7.0 Hz, 3H) ppm. ^13^C NMR (126 MHz, DMSO-*d*_6_) δ 161.78, 138.59, 128.94, 128.81, 128.39, 126.73, 51.30, 48.57, 41.92, 39.92, 39.75, 39.58, 27.65, 17.78 ppm. ^77^Se NMR (95 MHz, DMSO-*d*_6_) δ 428.80 ppm. MS (ESI) *m*/*z* calcd for C_17_H_24_N_6_Se [M+H]^+^ 393.13, found [M+H]^+^ 393.17.

#### 4-(1-(benzylselanyl)propyl)-6-(4-methylpiperazin-1-yl)-1,3,5-triazin-2-amine (**18**)

Prepared according to Ali et al.^94^

#### 4-(3-(benzylselanyl)propyl)-6-(4-methylpiperazin-1-yl)-1,3,5-triazin-2-amine(**19**)

Prepared according to Ali et al.^94^

### Molecular Modeling

The 5-HT_6_R homology model in its inactive conformation was fetched from the GPCRdb.^98,99^ The protein was prepared for docking in the Protein Preparation Wizard (Schrödinger Suite, version 2022-4) with the protonation states generated for pH 7.4. The preparation of compounds for docking was carried out in LigPrep (protonation states also generated for pH 7.4). The docking was carried out using the Induced Fit Docking panel (binding site centering on the ASP106; D3x32) in extra precision. The obtained docking poses were visualized in Pymol.

### Neurotoxicity

Human neuroblastoma cell line SH-SY5Y (ATCC no. CRL-2266) was used for neurotoxicity evaluation. The cells (8 × 10^3^ cells/100 μL/well) were cultured in transparent 96-well plates (Nunc) in DMEM/F12 supplemented with 10% FBS in the presence of DMSO < 0.1%, vehicle control (Veh) or increasing concentration of **13**–**15** (0.78 × 10^–6^–50 × 10^–6^ M, performed as 2-fold serial dilution for dose–response analysis). The highest concentration tested (50 μM) was due to the solubility limit of the compounds in the culture medium. Treatment with compounds was performed for 27 h. After the incubation time, the cell viability was examined using an MTS-based CellTiter96 AQueous One Solution Cell Proliferation Assay (Promega, Madison, WI, USA) following the manufacturer’s protocol. Briefly, 20 μL of MTS solution was pipetted into each well containing 100 μL of culture or culture medium (negative control) and incubated at 37 °C for 1 h. The absorbance was measured at 490 nm using the multimode plate reader Tecan Spark (Tecan, Männedorf, Switzerland). A reference wavelength of 630 nm was used to subtract the background. IC_50_ values were calculated by fitting a nonlinear regression to a sigmoidal dose–response curve in GraphPad Prism version 8.0.1.

### Neuroprotection in SH-SY5Y

To investigate the neuroprotective effect of compounds, two methods were used since rotenone impairs mitochondrial energy metabolism and increases ROS. The first method is MTS-based viability assays (an improved version of MTT) commercially available from PROMEGA. The second method is based on ROS measurement with 2′,7′-dichlorofluorescein diacetate (2′7′DCFH2-DA).

All treatments (except seeding the cells on the first day) were carried out with warmed HBSS containing 25 mM HEPES (hereafter referred to as HBSS), and during the operational steps, the cells were kept at 37 °C to minimize temperature stress. SH-SY5Y (ATCC no. CRL-2266) cells (2 × 10^4^ cells/well) were seeded in a black-sided, clear-bottom 96-well plate (Life Technologies) in DMEM/F12 supplemented with 10% FBS and cultured for 24 h. On the following day, the medium was removed from the cells, and they were washed once with HBSS and treated with the non-fluorescent dye 2′7′ DCFH_2_-DA (Millipore 287810, final concentration 50 μm, freshly prepared in warm HBSS) for 45 min. In the following step, the cells were washed once with HBSS following a 1 h pretreatment with HBSS containing the tested compounds **13**–**15** at a concentration of 10 μM. After that, rotenone was added at concentrations of 32.5 μM or 6.25 μM, and the cells were exposed for 3 h (ROS assay) or 24 h (MTS assay), respectively. To determine the most appropriate concentration of rotenone that results in 50% cell death, we tested various concentrations of rotenone in our preliminary experiments. Positive control was conducted by incubating the cells alone with rotenone in an adequate concentration. As a vehicle control, the cells were incubated in HBSS with 0.1% DMSO. The absorbance (MTS assay) was measured at 490 nm using the multimode plate reader Tecan Spark (Tecan, Männedorf, Switzerland). A reference wavelength of 630 nm was used to subtract the background. The fluorescence (2′7′DCFH2-DA assay) was measured at Ex/Em = 505/550 nm with the same multimode plate reader.

### Analysis of the Antioxidant Properties

#### Total Antioxidant Capacity

The total antioxidant capacity of compounds was assessed by the phosphomolybdenum method.^105^ This method is routinely applied in the laboratory to screen samples for natural sources of vitamins and powerful antioxidants. It evaluates both water-soluble and fat-soluble antioxidants (total antioxidant capacity). The methodology of this study is based on the reduction of Mo(VI) to Mo(V) by antioxidant compounds, followed by the formation of a bluish-green phosphate/Mo(V) complex at acidic pH, with maximum absorption at 695 nm. A 0.1 mL aliquot of the sample solution in dimethyl sulfoxide (in different concentrations) containing the reducing compound was combined in an Eppendorf tube with 1.0 mL of the reagent solution (0.6 M sulfuric acid, 28 mM sodium phosphate, and 4 mM ammonium molybdate, mixed by volume 1:1:1). The tubes were capped and incubated in a thermal block at 95 °C for 90 min. After cooling the samples to room temperature, the absorption of the solutions was measured at a wavelength of 695 nm using a UV–vis spectrophotometer relative to the blank. A blank solution containing 1.0 mL of reagent solution and 0.1 mL of dimethyl sulfoxide was incubated under the same conditions as the rest of the samples. Ascorbic acid (solutions in dimethyl sulfoxide; the concentration range from 10 to 230 mg per 1 mL) was used as the positive control. For the samples of the analyzed compounds, the total antioxidant capacity was estimated as the equivalent of ascorbic acid (AAE) by using the following equation:
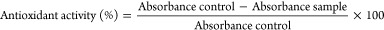


The experiments were carried out in triplicate, and results are given as the arithmetic mean. The data in all the experiments were analyzed using Statistica software.

#### RNA Extraction, Reverse Transcription, and qPCR

RNAs were extracted by ReliaPrep RNA Tissue Miniprep System (Promega) and reverse transcribed with iScriptTM cDNA Synthesis Kit (Bio-Rad Laboratories). cDNAs were amplified by qPCR reaction using GoTaq qPCR Master Mix (Promega), as reported in ref (94). Relative amounts obtained with the 2(−ΔC_t_) method were normalized with respect to the housekeeping gene L32. The relative PCR primers’ sequences are reported in Table 14.

**Table 14:** PCR Primers’ Sequences

**Primer name**	**Primer sequence**
hL32 FW	GGAGCGACTGCTACGGAAG
hL32 REV	GATACTGTCCAAAAGGCTGGAA
hNRF2 FW	AGGTTGCCCACATTCCCAAA
hNRF2 REV	ACGTAGCCGAAGAAACCTCA
hHO-1 FW	ACCTTCCCCAACATTGCCAG
hHO-1 REV	CAACTCCTCAAAGAGCTGGATG
hBACE1 FW	CCCGGGAGACCGACGAA
hBACE1 REV	CACCAGGATGTTGAGCGTCT
hSOD1 FW	AGGCATGTTGGAGACTTGGG
hSOD1 REV	TGCTTTTTCATGGACCACCAG
hNQO1 FW	GCTGGTTTGAGCGAGTGTTC
hNQO1 REV	CTGCCTTCTTACTCCGGAAGG
hNFkB FW	GCTTAGGAGGGAGAGCCCA
hNFkB REV	CTTCTGCCATTCTGAAGCCG

#### Protein Extraction and Western Blot Analysis

For total protein extract, cells were lysed in Laemmli buffer, while for nuclear protein isolation, cells were lysed in Lysis Buffer (MgCl_2_ 1.5 mM, KCl 10 mM, Tris-HCl 20 mM pH 7.5, DTT 1 mM) and after 15 strokes with douncer, nuclei and cytoplasm were separated by centrifugation (1500 RCF, 4 °C, 5 min). Subsequently, the proteins were resolved on SDS-PAGE and transferred to 0.45 μm nitrocellulose membrane (162-0115; Bio-Rad Laboratories). The following primary antibodies were used for immunoblotting: α-NRF2 (ab137550, Abcam), α-GAPDH (MAB-374, Millipore Corp.), or α-H3 (06755, Millipore Corp.), the last two used as loading controls (of total and nuclear protein extracts). The immune complexes were detected with horseradish peroxidase–conjugated species-specific secondary antiserum α-rabbit 172-1019 and α-mouse 170-6516 (Bio-Rad Laboratories), then by enhanced chemiluminescence reaction (Bio-Rad Laboratories). Densitometric analysis of protein expression was performed by using the Fiji ImageJ image processing package.

#### Thiophenol Assay

The glutathione peroxidase (GPx)-like activity of tested compounds was determined using the thiophenol assay.^109^ In more detail, 20 μL of the sample (10 μM) was added to a mixture of H_2_O_2_ (90 μL; 37.5 mM) and PhSH in methanolic solution (90 μL; 10 mM). The absorbance increase due to the formation of diphenyldisulfide (PhSSPh) was monitored for 30 min (25 °C) at 305 nm by using the Cytation 1 Cell Imaging Multimode Reader (BioTeK Instruments Inc., Winooski, VT, USA). The kinetics of the reaction were compared with the control. Results were expressed as reaction rates, namely the time taken for 50% completion of the PhSH oxidation to its disulfide (*t*_1/2_) and rate constant (*K*).

#### Statistical Analysis

Data from at least three experiments, in which each treatment was performed in triplicate, are expressed as mean ± standard error (SE). The statistical analysis and data representation were performed by GraphPad Prism (Version 8.00) software (GraphPad Software, Inc., San Diego, CA, USA). The difference between treatments was evaluated using a one-way analysis of variance (one-way ANOVA), followed by Dunnett’s multiple comparison post-test, and considered statistically significant when a *p*-value < 0.05 was obtained.

### ADME Studies *In Vitro* and *In Vivo*

#### Parallel Artificial Membrane Permeability Assay (PAMPA)

To evaluate parallel artificial membrane permeability, a precoated PAMPA Plate System (Gentest, Corning, Tewksbury, MA, USA) was used as we described previously.^97^ Caffeine served as the reference compound with high permeability. Concentrations of the investigated compounds were quantified through liquid chromatography/mass spectrometry (LC/MS) using a Waters TQ Detector mass spectrometer (Waters Corp., Milford, MA, USA), employing an internal standard. The experimentation was replicated thrice to ensure reliability. The permeability coefficients (*Pe*) expressed in units of 10^–6^ cm/s were computed following the manufacturer’s provided formula.

#### Metabolic Stability

The evaluation of metabolic stability and metabolic pathways in Phase I involved the incubation of compounds with rat or human liver microsomes (rLMs) for 120 min at a temperature of 37 °C, adhering to a previously described protocol.^97^ The intrinsic clearance (CL_int_) and half-life (*t*_1/2_) of **15** were estimated by incubation in the presence of rat or human liver microsomes. The disappearance of compound (50 μM) was determined at 5, 15, 30, and 60 min of incubation in 100 mM Tris-HCl buffer (37 °C). The UPLC/MS Waters ACQUITY TQD system with the TQ detector (Waters, Milford, MA, USA) analysis with the use of IS allowed for determination according to the formulas provided by Obach.^125^

rLMs and hLMs were purchased from (Sigma-Aldrich, St. Louis, MO, USA). The LC/MS analysis was used as a tool for potential metabolite determination. The procedure was supported by the *in silico* prediction of possible metabolic pathways using MetaSite 8.0.1. Software (Molecular Discovery Ltd., Hertfordshire, UK).

#### HepG2 and HEK-293 Toxicity Studies

The *in vitro* toxicity of **15** was evaluated using *hepatoma* HepG2 (ATCC HB-8065) and HEK-293 (ATCC CRL-1573). Cells were grown under the previously described conditions.^73^**15** was incubated in 96-well plates with cells for 72 h in the final concentration range (0.1–100 μM), whereas the reference DX at 1 μM. The cells’ viability was determined by CellTiter 96 AQueous Non-Radioactive Cell Proliferation Assay (MTS), which was purchased from Promega (Madison, WI, USA). The absorbance was measured using a Spark Cyto microplate reader (Tecan, Männedorf, Switzerland) at 490 nm. **15** was tested in triplicate. Data are presented from two independent experiments.

#### Instrumentation and Chromatographic Conditions

The *in vitro* evaluation of metabolic stability was performed by 120 min incubation of compounds with rat liver microsomes (rLMs) at 37 °C according to the previously described procedure.^97,126^ rLMs were provided by (Sigma-Aldrich, St. Louis, MO, USA). Determination of the most probable structures of 5-HT_6_R ligands’ metabolites was performed using LC/MS analyses.

The levels of **15** in serum and tissues were measured by a simple and sensitive reversed-phase high-performance liquid chromatography method with ultraviolet detection (HPLC/UV). The HPLC analysis was carried out on a Merck-Hitachi LaChrom Elite series liquid chromatographic system (Merck-Hitachi, Japan) equipped with an L-2130 pump (Hitachi LaChrom Elite), L-2300 thermostated column compartment (Hitachi LaChrom Elite), L-2130 vacuum degasser (Hitachi LaChrom Elite), L-2200 autosampler (VWR, Darmstadt, Germany), and L-2400 ultraviolet–visible (UV–vis) detector (Hitachi LaChrom Elite). Data acquisition was controlled by an EZChrome Elite v. 3.3.2 software (VWR, Darmstadt, Germany) chromatographic workstation. Chromatographic separation was accomplished on a Supelcosil LC-PCN (250 mm × 4.6 mm i.d., 5 μm particle size) analytical column, protected with the Supelcosil LC-PCN guard column (Sigma-Aldrich, Germany) under isocratic conditions. The mobile phase was a mixture of potassium dihydrogen phosphate buffer (0.01 M, pH 4.6) filtered through a 0.22-μm membrane filter (Sigma-Aldrich) with acetonitrile and methanol (51:9:40, v/v/v). The mobile phase was pumped from the reservoir to the column at a flow rate of 1 mL/min. Chromatograms were monitored at 218 nm, and the column temperature was maintained at 38 °C. The retention times (*t*_R_) for **15** and internal standard (IS) were approximately 9.3 and 15.1 min, respectively. The total run time for each sample analysis was 17 min. The calibration curve of **15** constructed by plotting the ratio of the analyte to the IS peak area versus the concentration of analyte was linear in the range of 5–100 ng/mL in rat serum and 5–100 ng/g in brain and heart homogenates, 5–200 ng/g in liver, kidneys and lungs homogenate with a coefficient correlation (*R*) value >0.994 in each case. The interday and intraday precision and accuracy of quality control samples, evaluated in both serum and tissue homogenates, were all within 10%. The lower limit of quantification was 5 ng/mL in serum and 5 ng/g in all analyzed tissue homogenates. The mean extraction recoveries were around 87.5% and 89.2% for serum and tissue homogenates, respectively. The mean recovery of IS was 88.3%.

#### Preparation of Serum and Tissue Samples

All frozen samples were thawed at room temperature before an extraction process.

Samples of rat serum (0.5 mL) were combined with 10 μL of the IS (4-(4-methylpiperazin-1-yl)-6-(1-phenoxypropyl)-1,3,5-triazine-2-amine) methanol solution at 25 ng/mL in a microcentrifuge polypropylene tube 2 mL (Eppendorf). The samples were alkalized with 50 μL of 4 M sodium hydroxide solution, vortex-mixed, and extracted with 1 mL of ethyl acetate:hexane (30:70, v/v) mixture on a shaker (VXR Vibrax, IKA, Germany) for 20 min. The samples were then centrifuged (Eppendorf, Mini Spin Plus, Bionovo, Poland) at 14,000*g* for 10 min, and the organic layers were transferred into new Eppendorf tubes (1.5 mL) containing 100 μL of methanol and 0.1 M sulfuric acid (10:90, v/v) mixture. Then, the samples were shaken and centrifuged again. Finally, 10–80 μL of each acidic layer was injected into the HPLC system.

Tissue samples were thawed, weighed (0.2–0.25 g), and homogenized using a MICCRA D-1 homogenizer (ART Prozess & Labortechnik GmbH & Co., Germany) in physiological saline (1:4, w/v), after which 10 μL of an IS solution (50 ng/mL) was combined with 500 μL of each tissue (brain, heart, liver, kidney, and lung) homogenate sample. These homogenates were then processed and analyzed identically to serum samples.

### Off-Target Radioligand Binding Studies

10 mM stock solutions of tested compounds were prepared in DMSO. Each compound was tested in a screening assay at a final concentration of 1 μM. Results were expressed as percent inhibition of specific control binding. Radioligand binding was performed on membranes from CHO-K1 cells, which were stably transfected with the human muscarinic M_1_ and histamine H_3_ receptor. Adrenergic α_2_ (α_2_-AR), cannabinoid CB_1_, and NMDA (MK-801) receptors were prepared from rat cortex tissue. Binding experiments were conducted in 96-well microplates, and the reaction mix included a solution of the test compound, radioligand, and diluted membranes or the tissue suspension. Specific assay conditions for each target are shown in Table 15. The reaction was terminated by rapid filtration through GF/B or GF/C filter mate presoaked with 0.3% polyethyleneimine for 30 min. Ten rapid washes with 200 μL of 50 mM Tris buffer (4 °C, pH 7.4) were performed using an automated harvester system Harvester-96 MACH III FM (Tomtec). The filter mates were dried at 37 °C in a forced air fan incubator, and then solid scintillator MeltiLex was melted on filter mates at 90 °C for 5 min. Radioactivity was counted in a MicroBeta2 scintillation counter (PerkinElmer) at approximately 30% efficiency.

**Table 15:** Radioligand Binding Assay Conditions

**Receptor**	**Radioligand/final concentration**	**Blank (nonspecific)**	**Assay buffer**	**Incubation conditions**
M_1_	[3H]-Scopolamine,	10 μM Atropine	PBS pH 7.4	120 min,
	0.3 nM			27 °C
H_3_	[3H]-*N*-α-Methylhistamine,	10 μM (*R*)-(−)-α-Methylhistamine	50 mM Tris-HCl, 5 mM MgCl_2_ pH 7.4	60 min,
	1.0 nM			27 °C
α_2_-AR	[3H]-Clonidine	10 μM Clonidine	50 mM Tris-HCl pH 7.6	30 min,
	4 nM			25 °C
CB_1_	[3H]-CP-55,940,	10 μM (*R*)-(+)-WIN55, 212-2	50 mM Tris-HCl, 5 mM MgCl_2_, 0.1% BSA pH 7.4	120 min,
	0.2 nM			24 °C
NMDA (MK-801)	[3H]-MK-801,	10 μM MK-801	50 mM Tris-HCl, 10 mM EDTA pH 7.4	120 min,
	5 nM			24 °C

### hERG Inhibition Studies

Electrophysiology experiments were carried out on a QPatch16X automatic patch clamp platform (Sophion Bioscience) using previously described methods.^127^ The intracellular flow channel of a disposable 16-site patch chip plate (QPlate 16 Large) was first primed using intracellular (IC) buffer containing 5.374 mM CaCl_2_, 1.75 mM MgCl_2_, 31.25 mM KOH, 10 mM EGTA, 10 mM HEPES, 120 mM KCl, and 4 mM Na2-ATP (pH 7.2, 290 mOsm). CHO cells, stably expressing the human ERG potassium channel (Kv11.1), were resuspended using robotic system of QPatch instrument in extracellular Ringer’s solution (EC = 2 mM CaCl_2_, 1 mM MgCl_2_, 10 mM HEPES, 4 mM KCl, 145 mM NaCl, 10 mM glucose, pH 7.4, 310 mOsm) and applied to the pipetting wells of QPlates. Gigaseals were formed following the standard protocol provided by Sophion Bioscience for CHO cells. During the experiment, cells were kept in a holding potential of −90 mV between the programmed stimulation protocols. Whole-cell potassium currents were measured in response to repeatedly executed voltage protocols that constituted of the following steps: brief clamping to −50 mV (200 ms), subsequent depolarization to 20 mV for 4000 ms, and final repolarization to −50 mV when the outward tail current was measured for 4000 ms. Reagents were added to the measurement sites of QPlate in the sequential application protocol, including the addition of saline and six increasing concentrations of tested compounds. After each reagent addition, voltage protocols were executed 10 times every 14 s. The electrophysiological recordings were analyzed using QPatch Assay Software (v5.0, Sophion Bioscience). The peak values of the tail currents for the last three voltage protocols after each vehicle or compound application were averaged, and the obtained means were taken for further data analysis employing Prism software (v8.4.3, GraphPad Software). The average current peak values determined in the presence of particular concentrations of tested compounds were subject to sigmoidal dose–response curve fitting, where the bottom plateau parameter was constrained to 0. Respective IC_50_ values were then calculated from the obtained sigmoidal dose–response curves. The final results represent the mean of three independent experiments carried out on distinct cells.

### Ames Mutagenicity Test

Ames microplate format assay was performed with *S. typhimurium* strain TA98, enabling the detection of frameshift mutations. Bacterial strain, as well as exposure and indicator medium, were purchased from Xenometrix AG (Allschwil, Switzerland), and the test was performed following the manufacturer’s instructions. The mutagenic potential of tested structures was evaluated by incubating bacteria incapable of producing histidine, with evaluated compounds for 90 min in exposure medium containing limited amount of histidine. The occurrence of reversion events to histidine prototrophy was observed as a growth of bacteria in the indicator medium without histidine after 48 h of incubation at room temperature. Bacterial growth in 384-well plates was visualized by the color change of the medium from violet to yellow due to the addition of pH indicator dye. The compound was classified as mutagenic if the fold increase in the number of positive wells over the solvent control baseline (FIB) was greater than 2.0. The FIB was determined by dividing the mean number of revertants for the tested compound by the solvent control baseline. The solvent control baseline was defined as the mean number of positive wells in the negative control sample, increased by one standard deviation (SD).

Some experiments were conducted in the presence of rat liver S9 fraction to simulate the metabolic activation of tested compounds. The S9 reaction mix for incubation with bacteria and tested compounds consisted of 30% (v/v) S9 preparation diluted in regeneration buffer containing 33 mM KCl, 8 mM MgCl_2_, 5 mM glucose-6-phosphate, 40 mM NADP, and 100 mM NaH_2_PO_4_. Rat liver S9 fraction was obtained from male Wistar rats (200–230 g). Livers were dissected and washed with 0.05 M Tris-HCl buffer (pH 7.4). Organs were weighed and chopped into small pieces using surgical scissors. Tissue was then homogenized in 4 vol (w/v) of Tris-HCl buffer at 24,000 rpm for 45 s, using an IKA Ultra-Turrax T18 homogenizer equipped with S18N-10G dispersing tool (Staufen im Breisgau, Germany) and obtained homogenate was centrifuged at 9000*g* for 20 min at 4 °C. The supernatant was collected, and the protein concentration in the obtained S9 fraction was adjusted to 30 mg/mL by dilution with an appropriate volume of Tris-HCl buffer.

### Pharmacokinetic Data Analysis

Basic PK parameters of **15** were obtained from non-compartmental analysis through the Phoenix WinNonlin (8.3 version, Pharsight, Certara Inc., Princeton, NJ, USA) program. The peak concentration (*C*_max_) and the time to reach *C*_max_ (*T*_max_) were obtained directly from individual concentration–time profiles. The linear trapezoidal rule was applied to calculate the areas under the concentration–time curve (AUC) from the time of dosing to the last measured data point (AUC_0–*t*_) or infinity (AUC_0–∞_). The terminal slope (λ_*z*_) was estimated by linear regression, and the terminal half-life (*t*_0.5λ*_z_*_) was calculated as ln 2/λ_*z*_. The clearance (CL/*F*) was calculated as *D*/AUC_0–∞_, and the volume of distribution based on the terminal phase (*V*_*z*_/*F*) was estimated as *D*/(λ_*z*_·AUC_0–∞_), where *F* is the bioavailability of i.p. administration. The mean residence time (MRT) was defined as AUMC_0–∞_/AUC_0–∞_, where AUMC is the area under the first moment curve. The tissue-to-serum AUC ratio (*K*_p_) of **15** was calculated by dividing the AUC_tissue_ by the AUC_serum_. Data are presented as mean ± SD.

### *In Vivo* Pharmacokinetic Studies (ADME)

#### Animals

The experiments were performed on male Wistar rats (200–230 g) obtained from an accredited animal facility at the Jagiellonian University Medical College, Poland. The animals were housed in a group of four in a controlled environment (ambient temperature 21 ± 2 °C; relative humidity 50–60%; 12-h light/dark cycles (lights on at 8:00)). Standard laboratory food (LSM-B) and filtered water were freely available. Animals were assigned randomly to treatment groups. All the experiments were performed by two observers unaware of the treatment applied between 9:00 and 14:00 on separate groups of animals. Prior to the PK experiments, the rats fasted for 12 h with free access to water. All animals were used only once. Procedures involving animals and their care were conducted following current European Community and Polish legislation on animal experimentation. Additionally, all efforts were made to minimize animals’ suffering and to use only the number of animals necessary to produce reliable scientific data. The experimental protocols and procedures described were approved by the I Local Ethics Commission in Cracow (no. 309/2019) and complied with the European Communities Council Directive of 24 November 1986 (86/609/EEC), and were following the 1996 NIH Guide for the Care and Use of Laboratory Animals.

#### Application to a Pharmacokinetic Study in Rats

To assess the PK profile and tissue penetration of **15**, the male Wistar rats received a single intraperitoneal (i.p.) injection with this compound dissolved in Tween (vehicle volume 1 mL/kg) at a dose of 1 mg/kg, determined in behavioral studies. The animals were killed by decapitation at 5, 15, 30, 60, and 120 min after administration of **15** (3–4 animals per time point), and blood samples (approximately 5–6 mL) were collected into tubes. Moreover, five tissues (i.e., brain, heart, lungs, liver, and kidneys) were harvested and rinsed with cold saline. Blood was allowed to clot for 15–20 min at room temperature and then centrifuged (2500 rpm for 10 min). The obtained serum and tissues were stored frozen at −80 °C until analysis.

### Behavioral Assays *In Vivo*

#### Animals

Male Wistar rats (8 weeks old, weighing 200–260 g) were obtained from an accredited animal facility at the Jagiellonian University Medical College, Poland. Animals were housed in groups of four in a controlled environment (ambient temperature 21 ± 2 °C; relative humidity 50–60%; 12-h light/dark cycles (lights on at 8:00)). Standard laboratory food (LSM-B) and filtered water were freely available. For 1 week before experiments, animals were handled to acclimate them to the researchers’ touch to minimize the stress reaction of animals. Rats were assigned randomly to treatment groups. All the experiments were performed by two observers unaware of the treatment applied between 9:00 and 14:00 on separate groups of animals. All rats were used only once. All compounds were i.p. injected in a volume of 2 mL/kg. Procedures involving animals and their care were conducted under current European Community and Polish legislation on animal experimentation. Additionally, all efforts were made to minimize animal suffering and to use only the number of animals necessary to produce reliable scientific data. Approval for the procedures described in this paper was obtained from the I Local Ethics Commission in Cracow (no. 309/2019, 17.07.2019), complied with the European Communities Council Directive of 24 November 1986 (86/609/EEC), and were under the 1996 NIH Guide for the Care and Use of Laboratory Animals.

#### Drugs

**15** was suspended in 1% Tween 80 immediately before administration, while MK-801 (MK-801 maleate, Bio-Techne, Warszawa, Poland) was dissolved in distilled water. All compounds were given in a volume of 2 mL/kg. **15** was administered i.p. for 60 min while MK-801 was given i.p. 30 min before testing. Control animals received vehicle (1% Tween 80 (Sigma-Aldrich, Poznań, Poland)) according to the same schedule.

#### Novel Object Recognition (NOR) Test

The protocol was adapted from the original work,^128,129^ and the test and the administration of compounds were done according to the previously described protocol (**15** and MK-801 were administered 60 and 30 min, respectively, before the T1 phase (the familiarization phase)).^73^ The discrimination index (DI) was calculated according to the formula
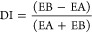
where EB is the exploration time of a novel object during T2 session and EA is the exploration time of a familiar object during T2 session.

MK-801 was chosen as the memory disturbance-induced compound based on the literature data, which indicates that ligands of 5-HT_6_R may prevent memory disturbances in rats induced by MK-801.^37,73^ To assess the impact of the injected compounds on the rats’ exploratory activity, the total exploration time in T2 phase was measured.

#### Forced Swim Test (FST)

The experiment was carried out according to the method of Porsolt;^130^ the procedure and administration of compounds were done according to the previously described protocol.^73^ Immobility was assigned when no additional activity was observed other than that necessary to keep the rat’s head above the water. Fresh water was used for each animal.

#### Elevated Plus-Maze (EPM) Test

The testing procedure was based on a method described by Pellow and File;^131^ the procedure and administration of compounds were done according to the previously described protocol.^73^ The EPM test is an “unconditional” anxiety-like test based on rodents’ natural aversion to heights and open space.

#### Exploratory Activity Measured in the EPM Test

The experiment was performed using the EPM apparatus (details see above). Total ambulation (the total distance covered by a rat and ambulation along the X and Y axes) was taken to discern drug effects on general activity from those on open-arm exploration during a 5 min test period (i.e., the time equal to the observation period in the EPM test). The rats’ behavior was not videotaped during the test.

### Statistical Analysis of Behavioral Studies

STATISTICA 13 (StatSoft) was used for the statistical analysis of results. All behavioral results are shown as the means ± SEM. The data were evaluated by an analysis of variance (one-way ANOVA), followed by Bonferroni’s multiple comparison test; *p* < 0.05 was considered significant.

## Supporting Information Available

Synthesis of compound **15O**; HPLC traces, mass spectra, and ^1^H, ^13^C, and ^77^Se NMR spectra of final compounds **3**, **6**–**15**, **15O**, and **17**; Table S1, elemental analysis of final compounds **3**, **6**–**15**, and **17**; Table S2, structures of intermediate compounds **31**–**42**; Figures S1–S3, additional data of molecular modeling; details for the functional bioassays for the 5-HT6 receptor; Figure S4, dose–response curves from functional in vitro assay for cAMP response of compounds **11**–**15** and references (Olanzapine, SB752457); Table S3, cAMP 5-HT_6_ functional assay for compound **15**; Table S4, absorbance results recorded for the analyzed compounds for all tested concentrations, compared to those for ascorbic acid (AA); Table S5, ascorbic acid equivalents (%AAE) for compounds **13**–**15** at different concentrations; Table S6, effect of compound **15** in the EPM test; Table S7, analysis of a possible cleavage of **13**–**15** along C(sp^3^)–Se bonds in the microsome assays, based on LC/MS results as well as Figures S5–S23, showing metabolic stability results; and Figure S24, dose-dependent inhibition of hERG channel activity by **15** (PDF)
PDB files of docking models for compounds **4**, **9**, **15**, **15O**, and **15S** (ZIP)
Molecular formula strings (CSV)

## Dedication

The article is dedicated to Thomas Zwergel, who passed away during its preparation. He was a dear friend who always irradiated benevolence, infectious optimism, an appreciation for life, an elegant self-irony, and a wonderful sense of humor. All authors would like to recognize him for the wonderful discussions about science and beyond.

## Abbreviations Used

2′7′DCFH_2_-DA: 2′,7′-dichlorofluorescin diacetate
5-HT_*x*_R: serotonin receptor 5-HT_*x*_
AA: ascorbic acid
AAE: ascorbic acid equivalents
ARE: antioxidant response element
BACE1: beta-site amyloid precursor protein cleaving enzyme 1
BBB: blood–brain barrier
BPSD: behavioral and psychological symptoms of dementia
FDA: U.S. Food & Drug Administration
GPx: glutathione peroxidase
hLMs: human liver microsomes
HO-1: heme oxygenase-1
MRT: mean residence time
MTS: colorimetric cell proliferation assay
MWM: Morris water maze
NOR: novel object recognition
NQO-1: quinone oxidoreductase-1
NRF2: nuclear factor erythroid 2-related factor 2
PhSSPh: diphenyl disulfide
PK: pharmacokinetic
RBA: radioligand binding assay
rLM: rat liver microsome
Sec: selenocysteine
SOD: superoxide dismutase
SRT: social recognition
Y-CAT: Y-maze continuous spontaneous alternation
