# Supplementary material for: First-in-Class Selenium-Containing Potent Serotonin Receptor 5-HT6 Agents with a Beneficial Neuroprotective Profile against Alzheimer’s Disease
Source: J Med Chem. 2024 Jan 8;67(2):1580–610. doi: 10.1021/acs.jmedchem.3c02148 (PMC10823479; doi:10.1021/acs.jmedchem.3c02148)
Supplement: Supplementary file 1 — jm3c02148_si_001.pdf [file jm3c02148_si_001.pdf]

Supporting Information

First-in-Class Seleniuim-Containing Potent Serotonin Receptor 5-HT6 Agents with a Beneficial Neuroprotective Profile against Alzheimer's Disease

Patryk Pyka,[†,‡,$,§] Wawrzyniec Haberek,[†,‡,$,§] Małgorzata Więcek,[†] Ewa Szymanska,[†] Wesam Ali,[†,‡] Agnieszka Cios,[⊥] Magdalena Jastrzębska-Więsek,[⊥] Grzegorz Satała,[‖] Sabina Podlewska,[‖] Silvia Di Giacomo,[#,¶] Antonella Di Sotto,[#] Sabrina Garbo,[∇] Tadeusz Karcz,[†] Chiara Lambona,[⊗] Francesco Marocco,[∇] Gniewomir Latacz,[†] Sylwia Sudoł-Tałaj,[†,$] Barbara Mordyl,[□] Monika Głuch-Lutwin,[□] Agata Siwek,[□] Kinga Czarnota-Łydka,[†,$] Dawid Gogola,[†,$] Agnieszka Olejarz-Maciej,[†] Natalia Wilczyńska-Zawal,[⊥] Ewelina Honkisz-Orzechowska,[†] Małgorzata Starek,[△] Monika Dąbrowska,[△] Katarzyna Kucwaj-Brysz,[†] Rossella Fioravanti,[⊗] Muhammad Jawad Nasim,[‡] Marius Hittinger,[○,▼] Anna Partyka,[⊥] Anna Wesołowska,[⊥] Cecilia Battistelli,[∇,*] Clemens Zwergel,[‡,⊗,○,*] and Jadwiga Handzlik[†,*]

[†] *Department of Technology and Biotechnology of Drugs, Jagiellonian University, Medical College, Medyczna 9, 30− 688 Kraków, Poland*

[‡] *Division of Bioorganic Chemistry, School of Pharmacy, Saarland University, Campus B 2.1, D−66123 Saarbrücken, Germany*

[$] *Doctoral School of Medical and Health Sciences, Jagiellonian University Medical College, św. Łazarza 15, 31− 530 Kraków, Poland*

[⊥] *Department of Clinical Pharmacy, Faculty of Pharmacy, Jagiellonian University, Medical College, Medyczna 9, 30− 688 Kraków, Poland*

[‖] *Department of Medicinal Chemistry, Maj Institute of Pharmacology, Polish Academy of Sciences, Smętna 12, 31−343 Kraków, Poland*

[#] *Department of Physiology and Pharmacology "V. Erspamer", Sapienza University of Rome, Piazzale Aldo Moro 5, 00185 Rome, Italy*

[¶] *Italian National Institute of Health (ISS), Viale Regina Elena 299, 00161 Rome, Italy*

[∇] *Department of Molecular Medicine, Istituto Pasteur Italia, Fondazione Cenci−Bolognetti, Sapienza University of Rome, Viale Regina Elena 324, 00161 Rome, Italy*

[⊗] *Department of Drug Chemistry and Technologies, Sapienza University of Rome, Piazzale Aldo Moro 5, 00185 Rome, Italy*

[□] *Department of Pharmacobiology, Faculty of Pharmacy, Jagiellonian University, Medical College, Medyczna 9, 30− 688 Kraków, Poland*

[△] *Department of Inorganic and Analytical Chemistry, Jagiellonian University, Medical College, Medyczna 9, 30− 688 Kraków, Poland*

[○] *Department of Drug Discovery, Pharmbiotec gGmbH, Nußkopf 39, 66578 Schiffweiler, Germany*

[▼] *Department of Drug Delivery, Pharmbiotec gGmbH, Nußkopf 39, 66578 Schiffweiler, Germany*

[§]*equal contribution*

* *corresponding authors (C.B. cecilia.battistelli@uniroma1.it, C.Z. clemens.zwergel@uniroma1.it and J.H. j.handzlik@uj.edu.pl*

Table of contents

Synthesis of compound 15O S3

HPLC-traces, Mass-spectra and ^{1}H, ^{13}C, and ^{77}Se NMR spectra of final compounds 3, 6-15, 15-O, and 17 .... S4

Table S1. Elemental analysis of final compounds 3, 6-15, and 17 .... S32

Table S2. Structures of intermediate compounds 31-42 .... S33

Figures S1-S3 additional data of Molecular Modelling S34

Functional bioassays for 5-HT_6 receptor S37

Figure S4 Dose−response curves from functional in vitro assay for a cAMP response of compounds 11-15 and references (Olanzapine, SB752457) S38

Table S3. cAMP 5-HT6 functional assay for compound 15 .... S39

Table S4. The results of absorbance recorded for the analyzed compounds for all tested concentrations, compared to those for ascorbic acid (AA) S40

Table S5. The ascorbic acid equivalents (%AAE) for compounds 13, 14, and 15 at different concentrations. S40

Table S6. Effect of compound 15 in the EPM test S41

Figures S5-S23 Metabolic stability results. S42

Table S7. Analysis of a possible cleavage of 13-15 along C(sp3)-Se bounds in the microsome assays, based on LC/MS results based on Figures S5-S23. .... S53

Figure S24 - Dose-dependent inhibition of hERG channel activity by 15 .... S54

References S54

Synthesis of compound 15O

The final compound **15O** was obtained according to the synthetic path and procedure previously described by us. [1]

*General procedure for the synthesis of reference compound **15O***

Sodium (10 mmol) was dissolved in 20 ml of absolute methanol, then 4-methylpiperazine-1-yl biguanide hydrochloride (5 mmol) and methyl α-(β-naphthyloxy)-isobutyrate (5 mmol) was added. The reaction mixture was refluxed for 16 hours. Subsequently, the reaction mixture was cooled to room temperature, quenched with 10 ml water, and the resulting precipitate was isolated by filtration. Crystallization from methanol gave the desired final product as a white solid.

4-(4-methylpiperazin-1-yl)-6-(2-(naphthalen-2-yloxy)propan-2-yl)-1,3,5-triazin-2-amine (**15O**) White solid, yield: 27.7 %. ^{1}H NMR (500 MHz, DMSO-d_6) δ 7.70 (d, J = 7.6 Hz, 1H), 7.64 (d, J = 9.0 Hz, 1H), 7.57 (d, J = 8.1, 0.5 Hz, 1H), 7.35 (ddd, J = 8.2, 6.8, 1.3 Hz, 1H), 7.28 (ddd, J = 8.1, 6.8, 1.3 Hz, 1H), 7.08 (dd, J = 8.9, 2.5 Hz, 1H), 7.00 (d, J = 2.4 Hz, 1H), 3.78 (s, 4H), 2.41 (s, 4H), 2.29 (s, 3H), 1.70 (s, 6H). ^{13}C NMR (126 MHz, DMSO-d_6) δ 134.12, 129.61, 127.99, 127.69, 126.89, 125.24, 125.23, 125.21, 122.92, 80.69, 42.43.MS (ESI) m/z calculated for $C_{21}H_{26}N_6O$ $[M]^+$ 378.22, found 379.37 $[M+1]^+$

The affinity of compound **15O** for the 5-HT$_6$ serotonin receptor was determined in a radioligand binding assay using the procedure described in the experimental section of the main article.

HPLC-traces, Mass-spectra and ^{1}H, ^{13}C and ^{77}Se NMR spectra of final compounds 3, 6-15, 15-O and 17

No	Code	Structure	MW	Purity
**3**	**WA-Se-5**	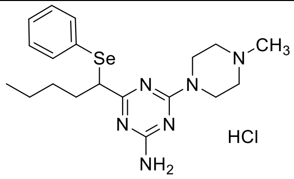	455.89	97.46 %

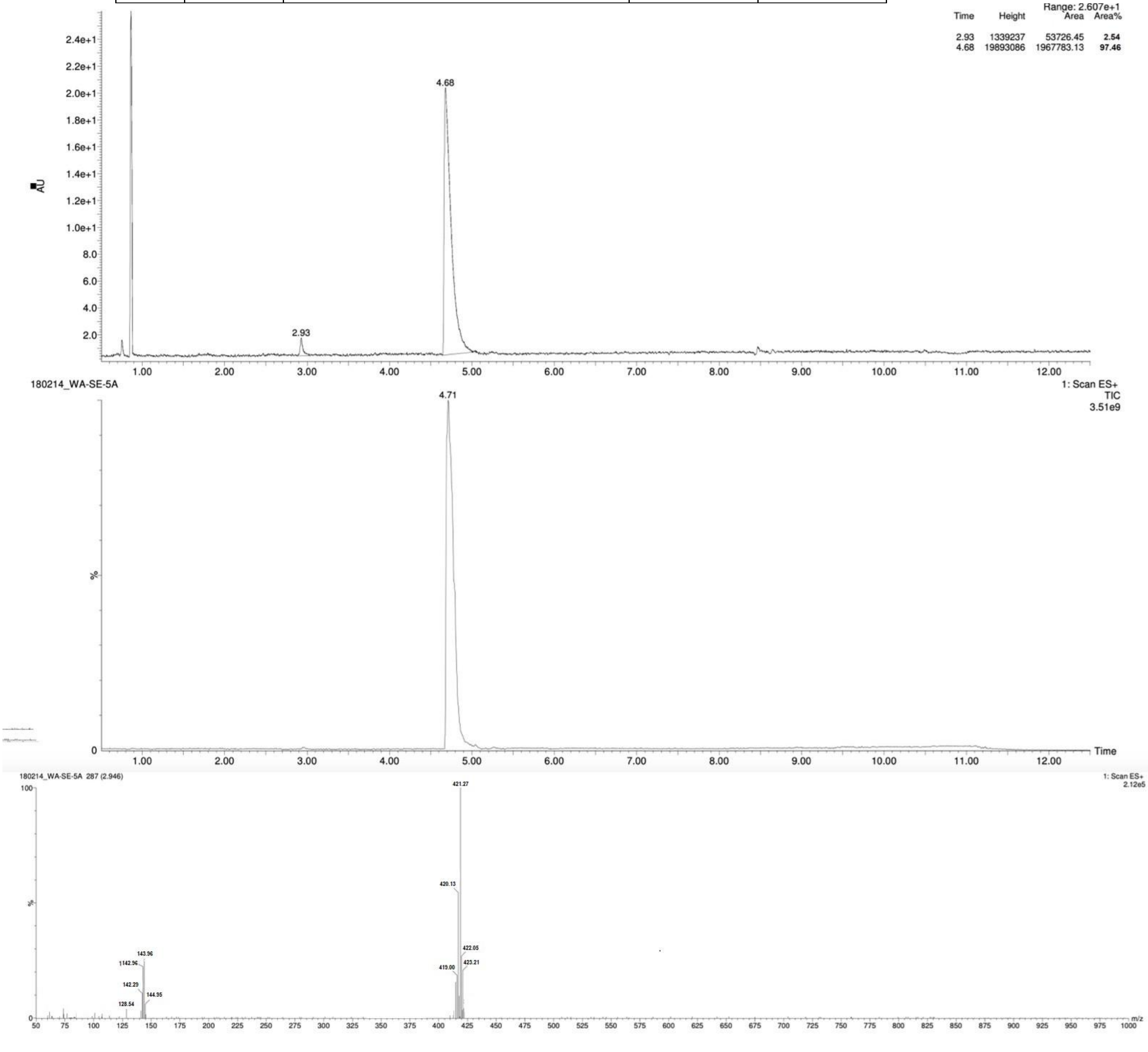

Mass and HPLC trace of compound **3**

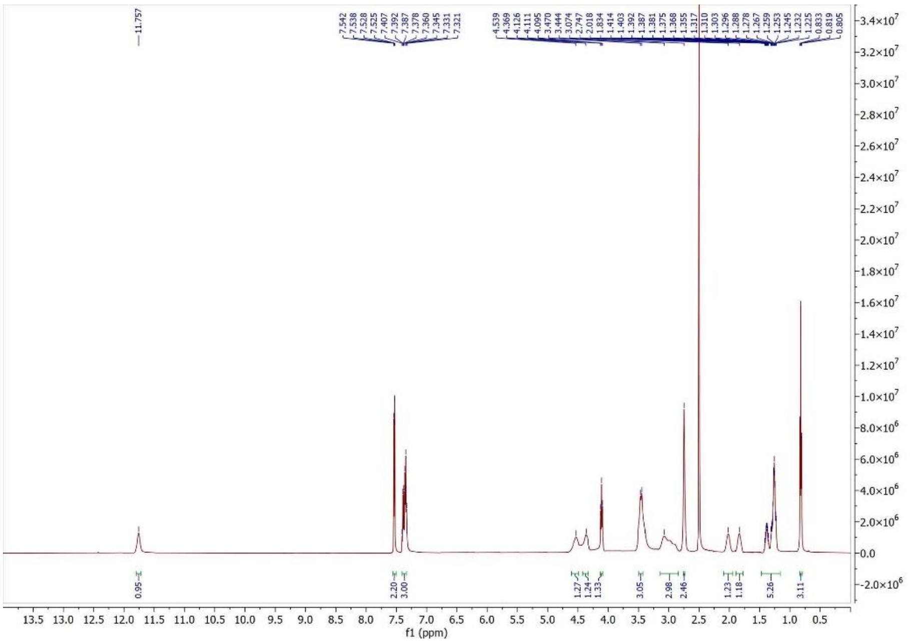

^{1}H NMR of compound **3**

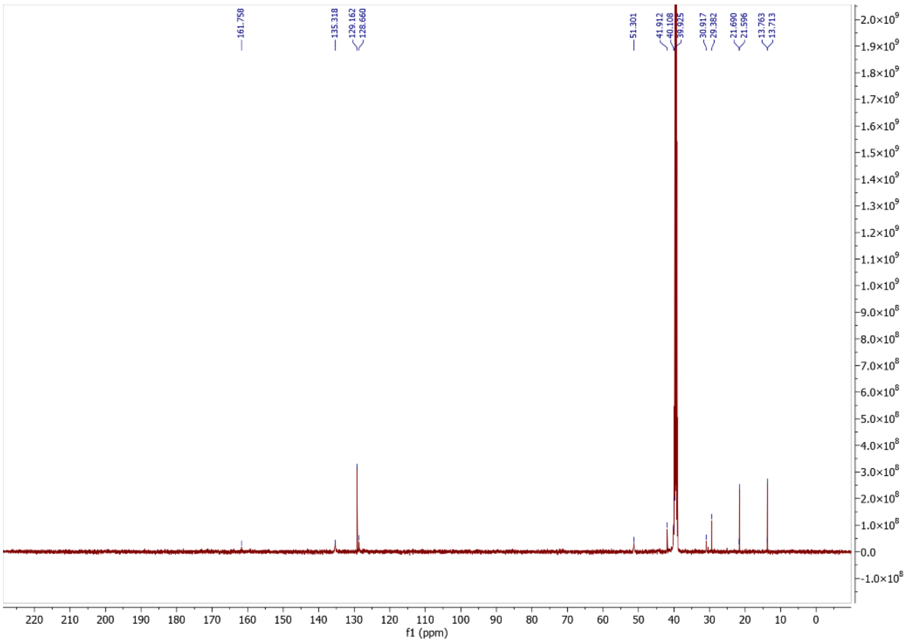

^{13}C NMR of compound **3**

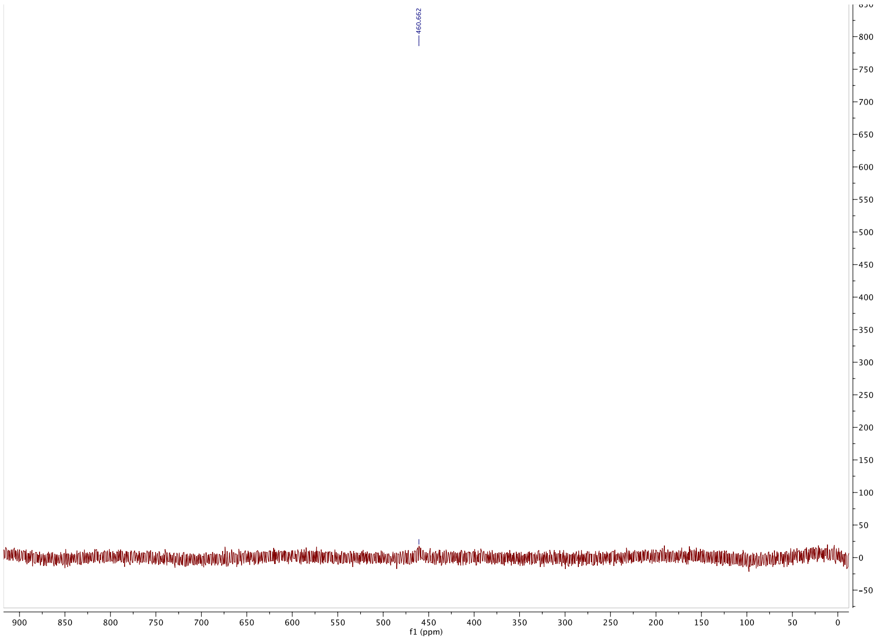

^{77}Se NMR of compound **3**

No	Code	Structure	MW	Purity
**6**	**MR-1b**	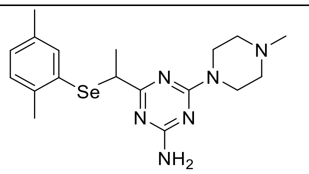	405.42	100%

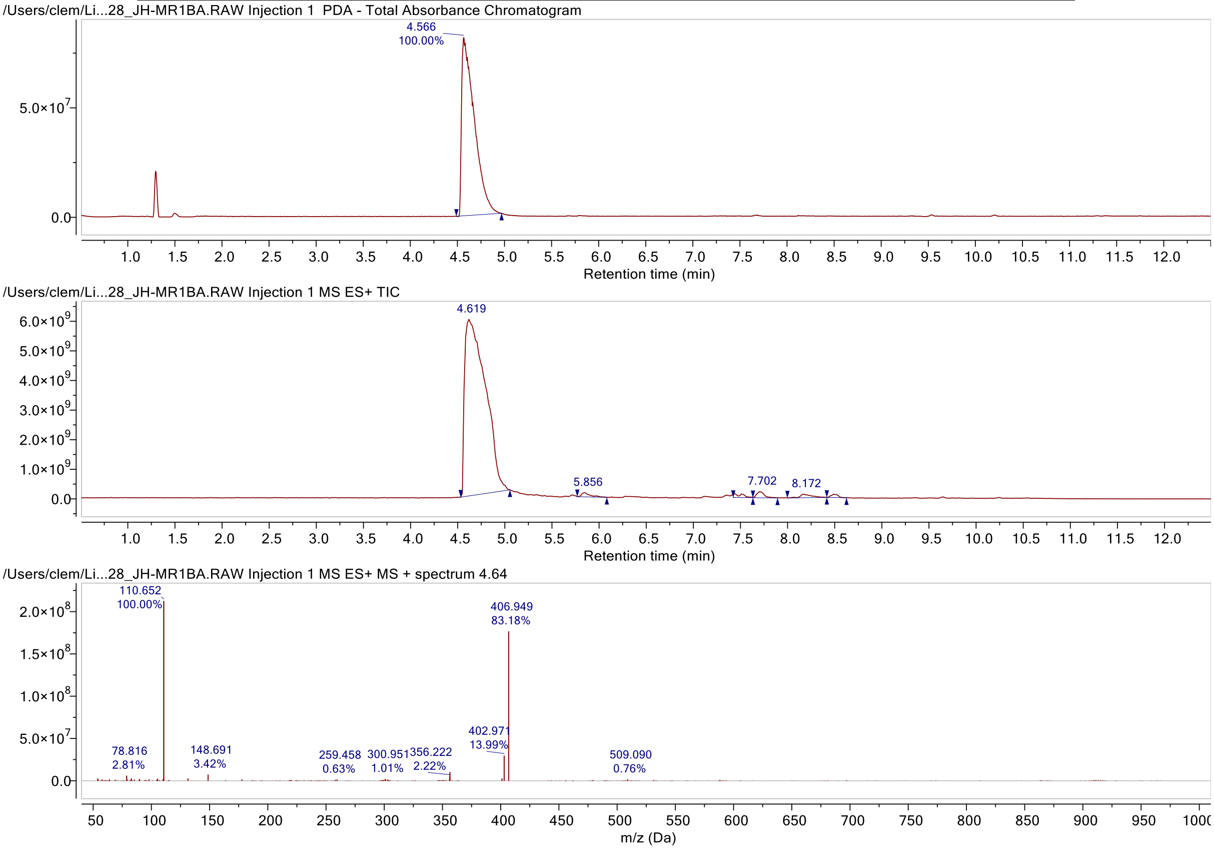

HPLC trace and mass spectrometry of compound **6**

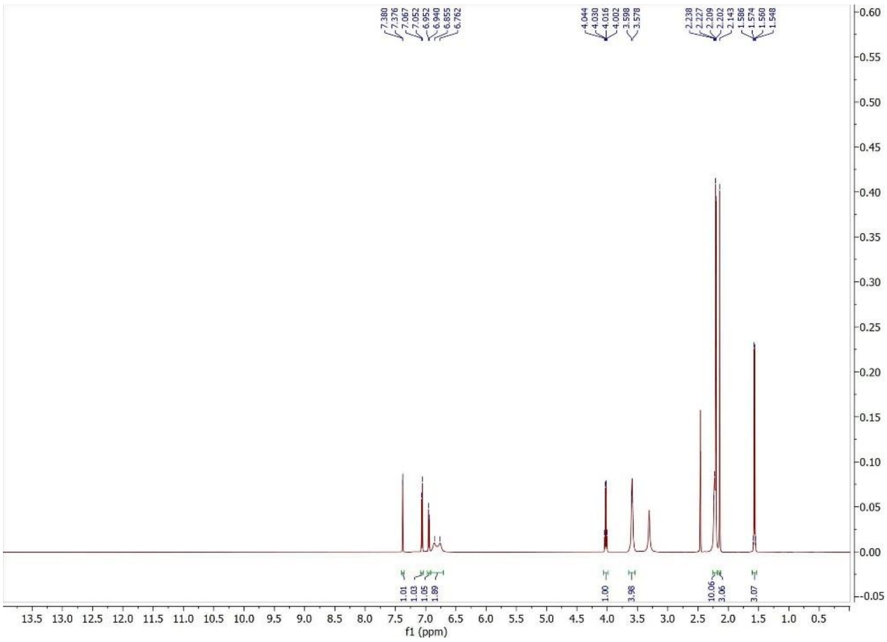

^{1}H NMR of compound **6**

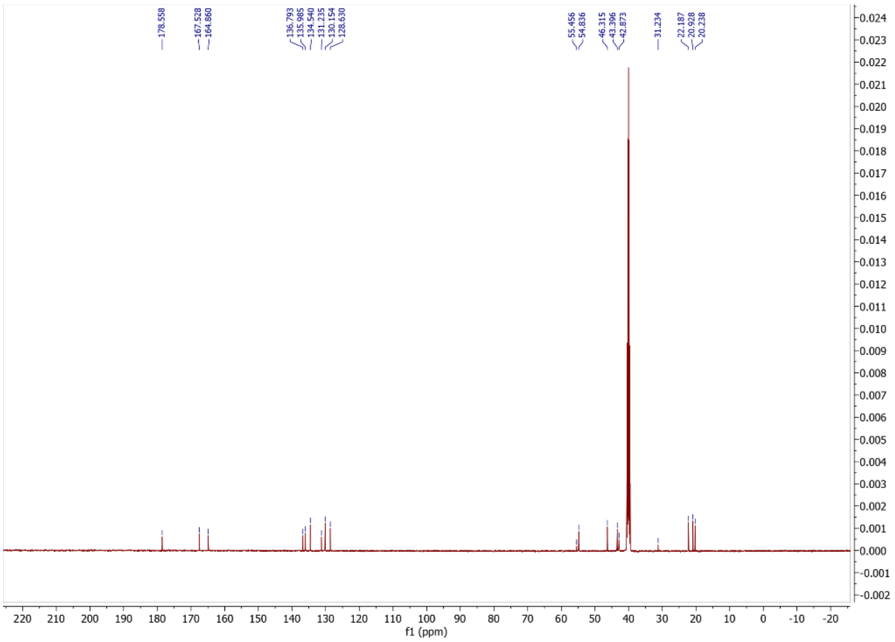

^{13}C NMR of compound **6**

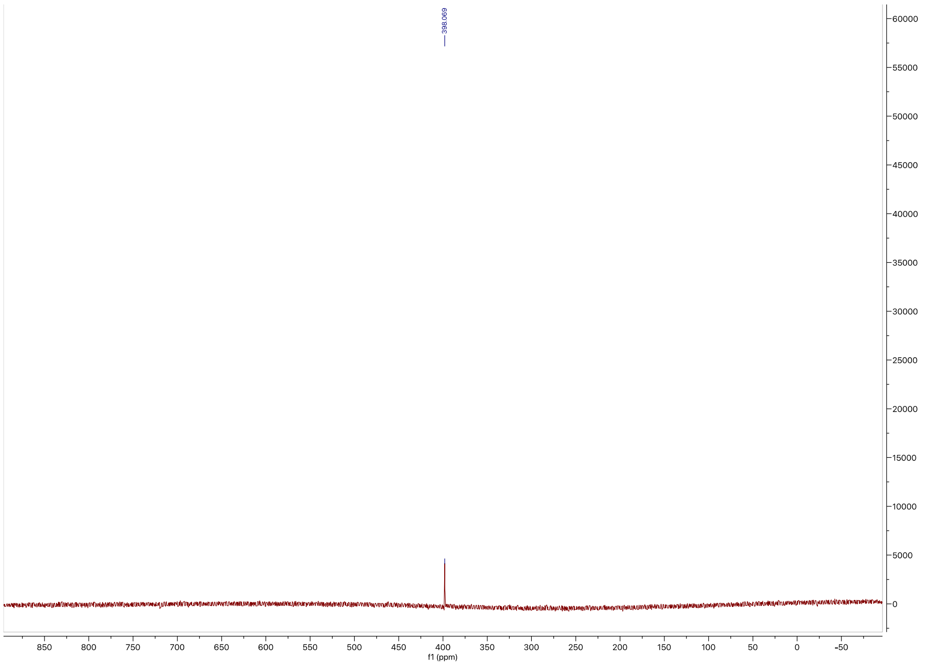

^{77}Se NMR of compound **6**

No	Code	Structure	MW	Purity
**7**	**MR-2b**	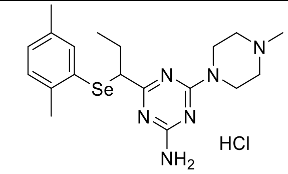	455.90	95.15%

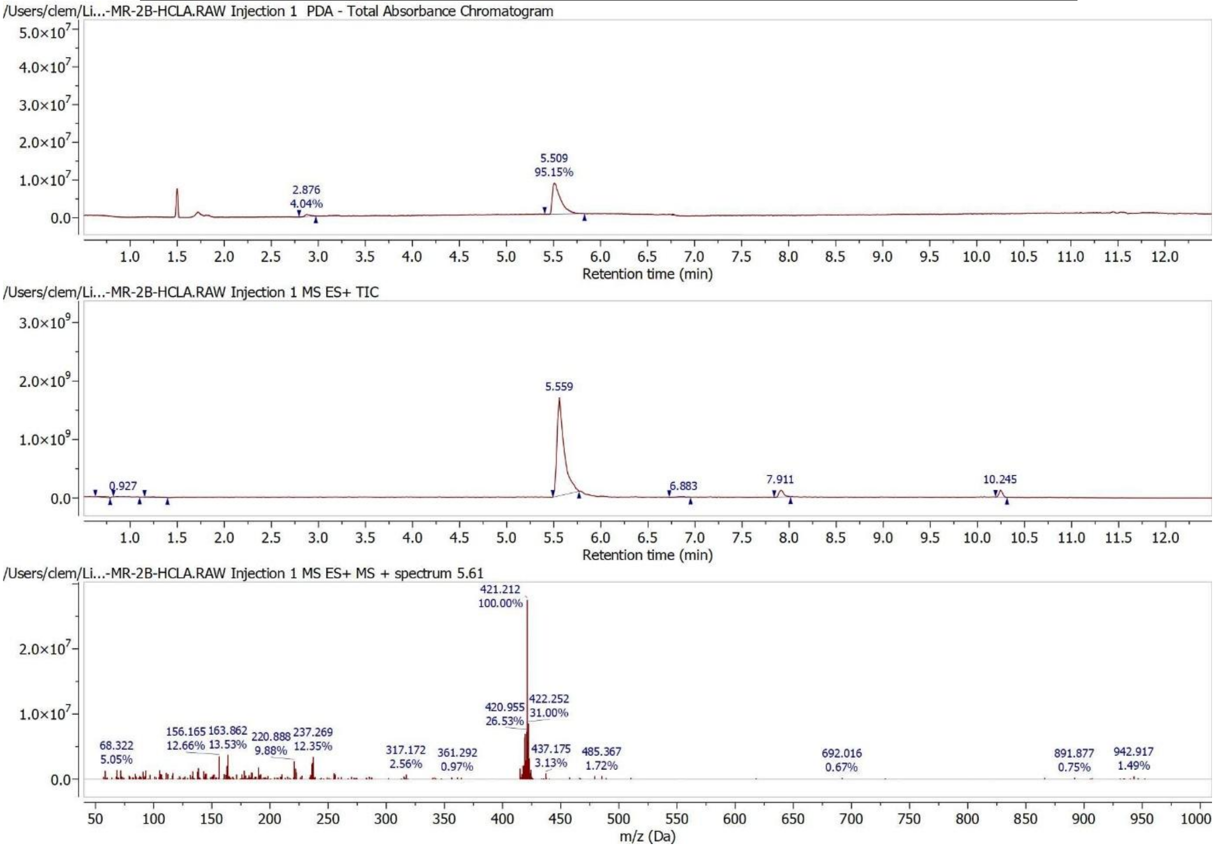

HPLC trace and mass spectrometry of compound **7**

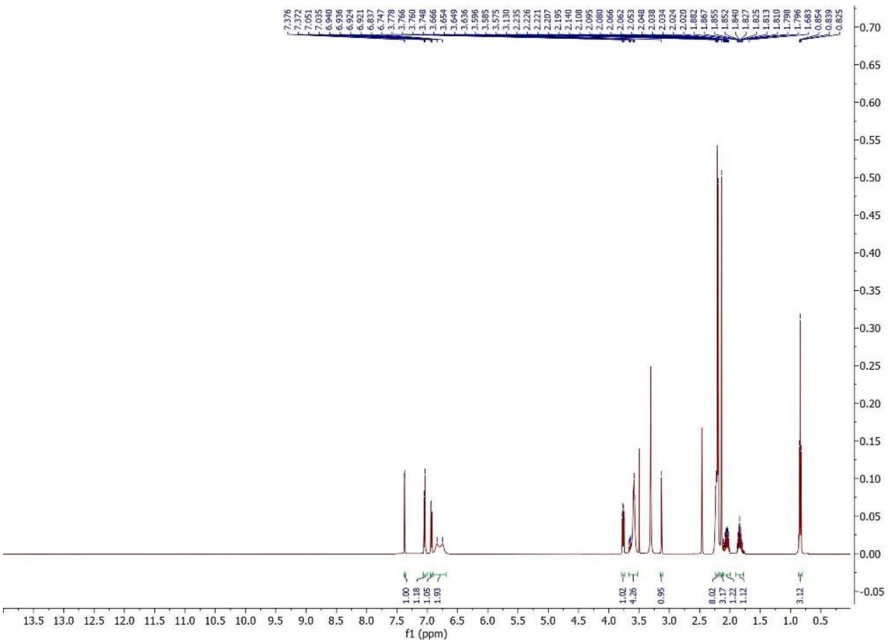

^{1}H NMR of compound **7**

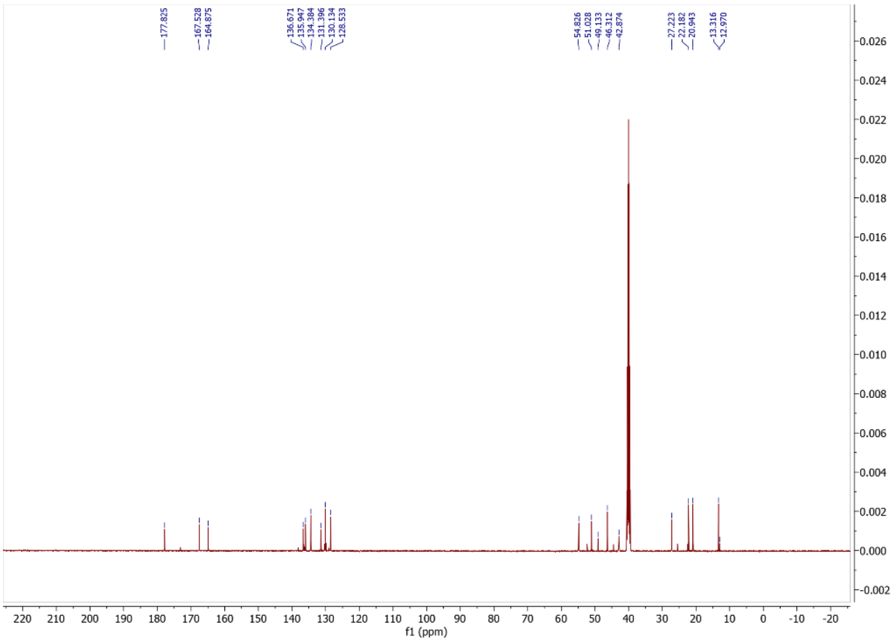

^{13}C NMR of compound **7**

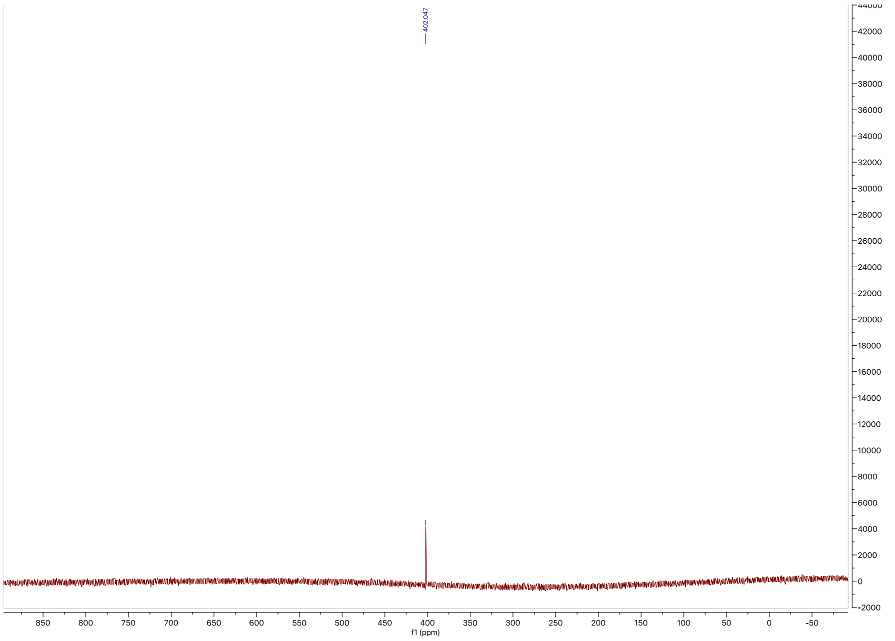

^{77}Se NMR of compound **7**

No	Code	Structure	MW	Purity
**8**	**WD-2**	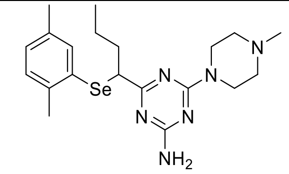	469.91	99.51%

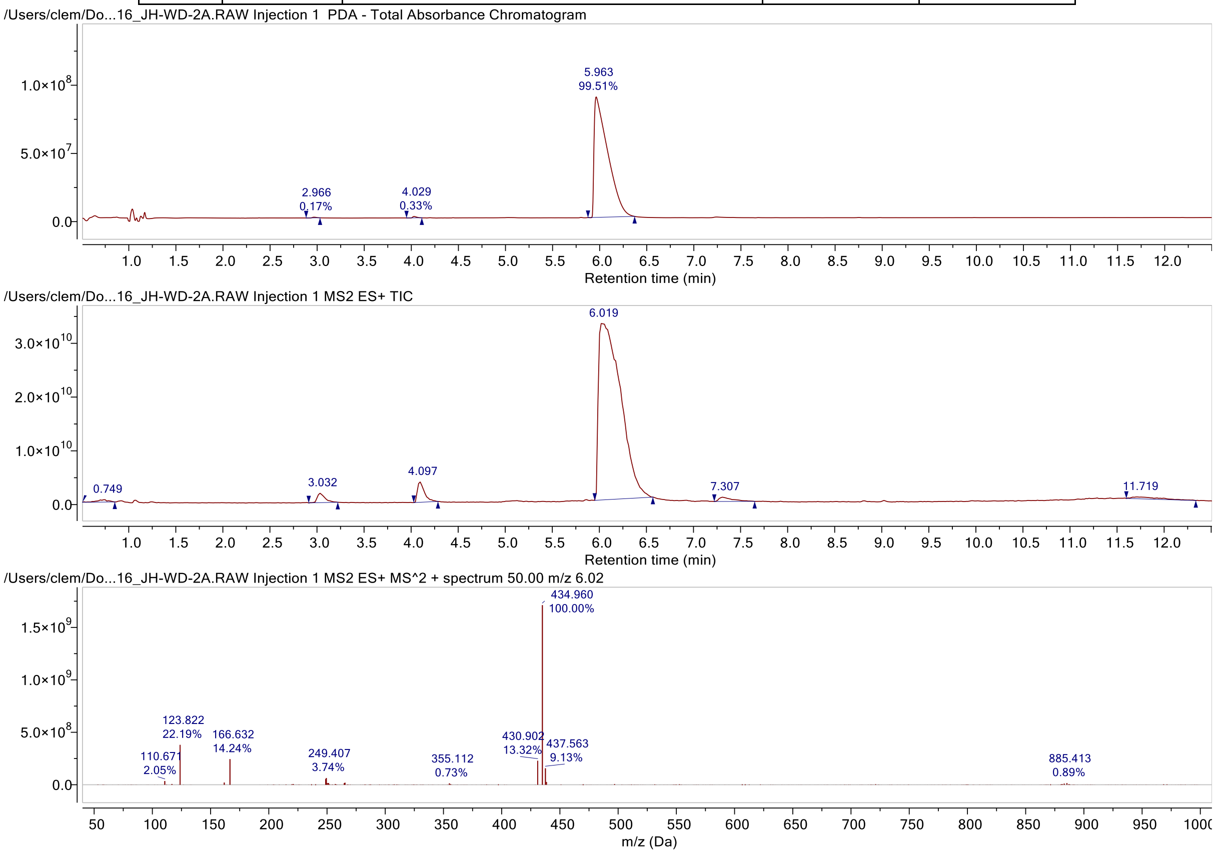

HPLC trace and mass spectrometry of compound **8**

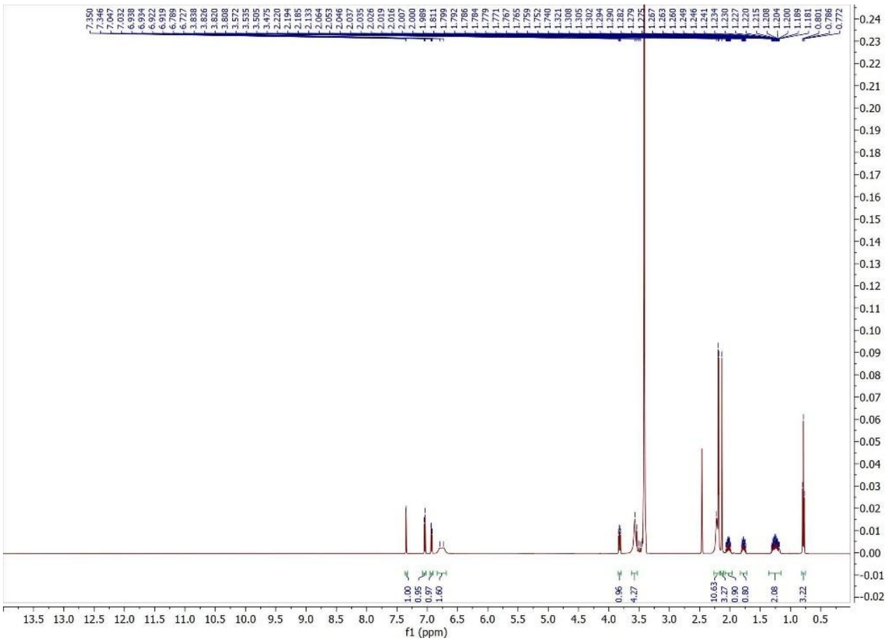

^{1}H NMR of compound **8**

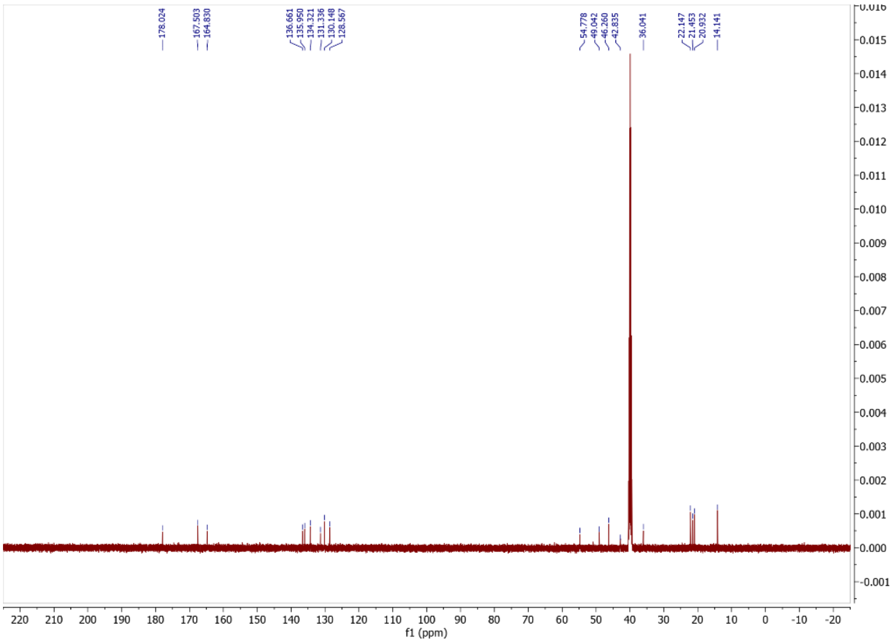

^{13}C NMR of compound **8**

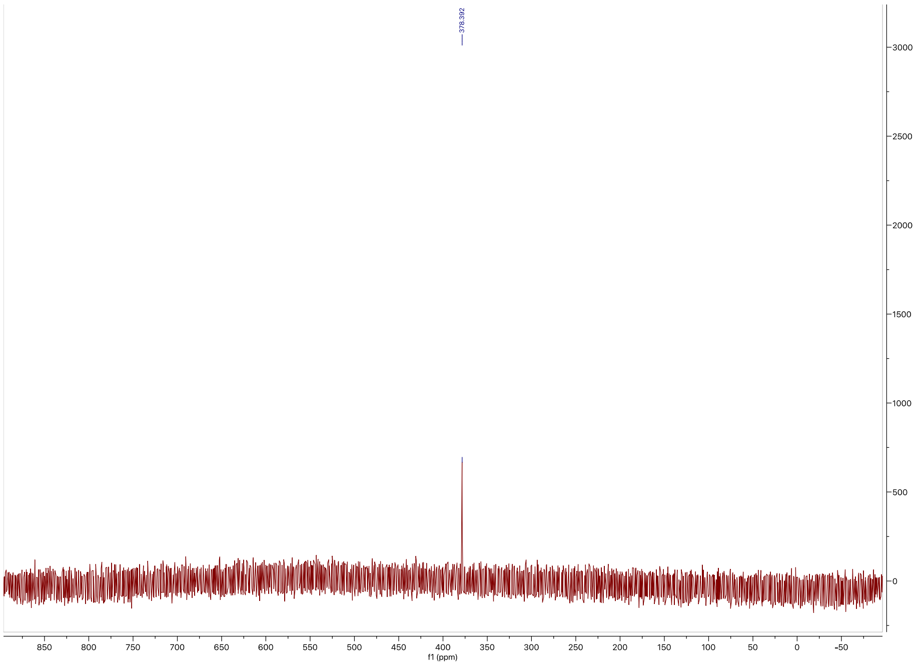

^{77}Se NMR of compound **8**

No	Code	Structure	MW	Purity
**9**	**WD-1**	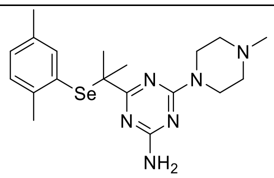	419.435	97.16%

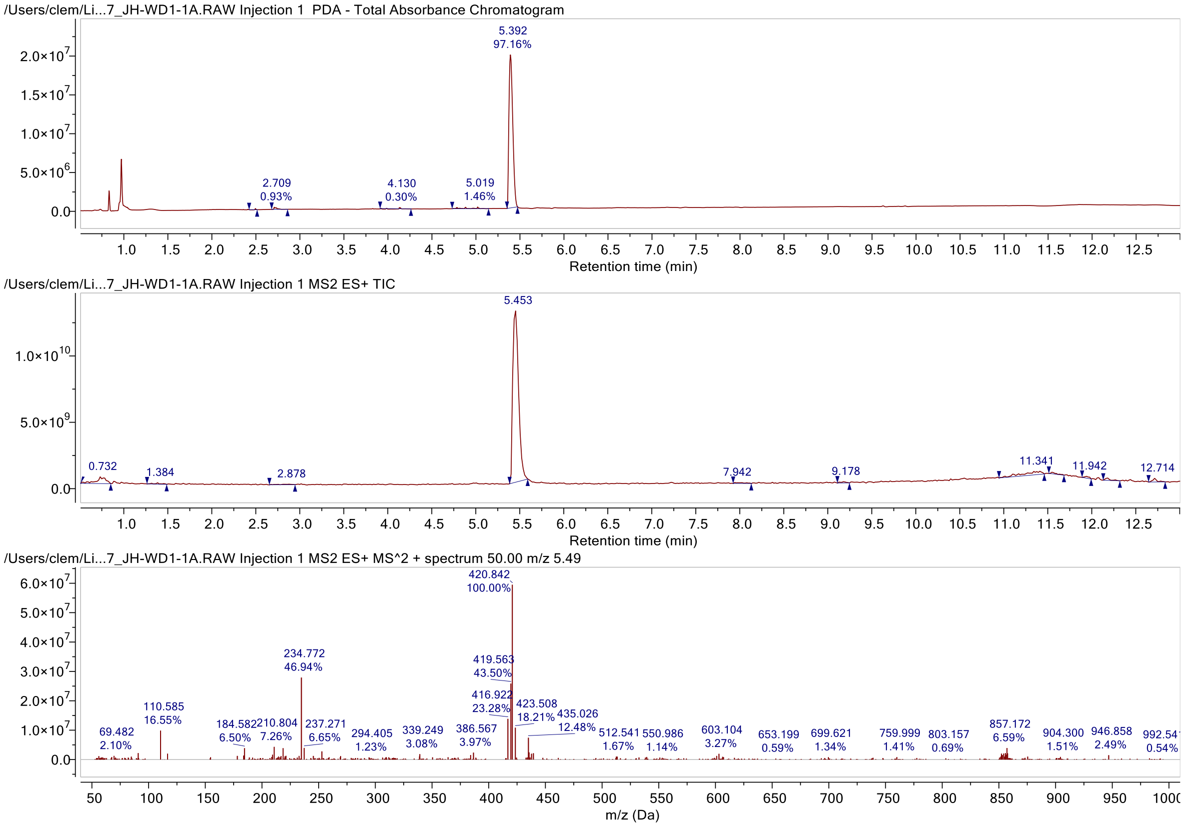

HPLC trace and mass spectrometry of compound **9**

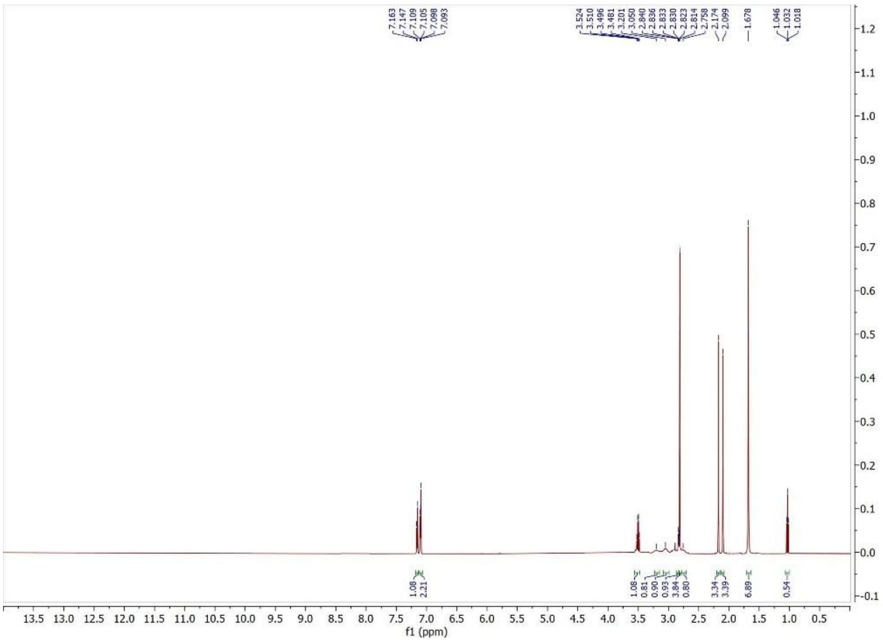

^{1}H NMR of compound **9**

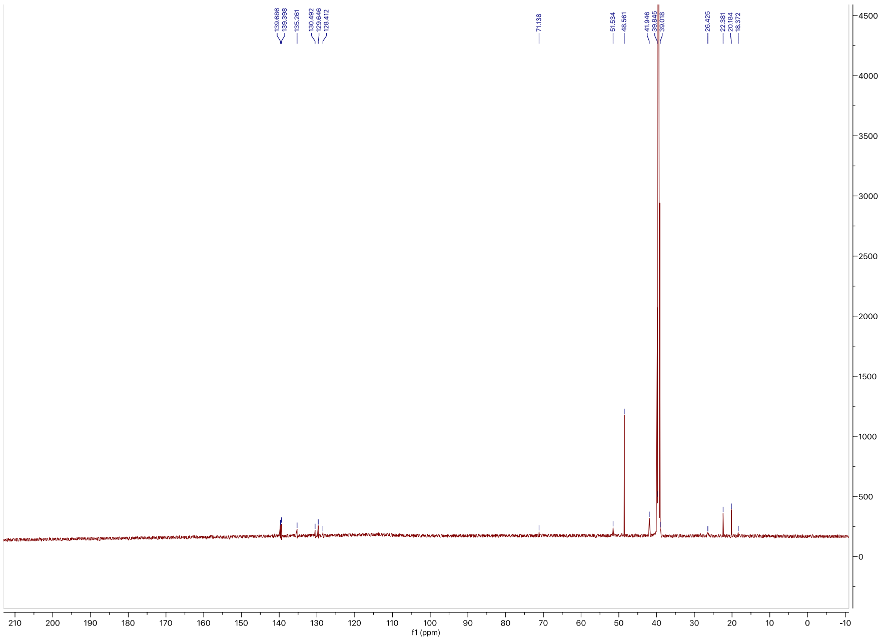

^{13}C NMR of compound **9**

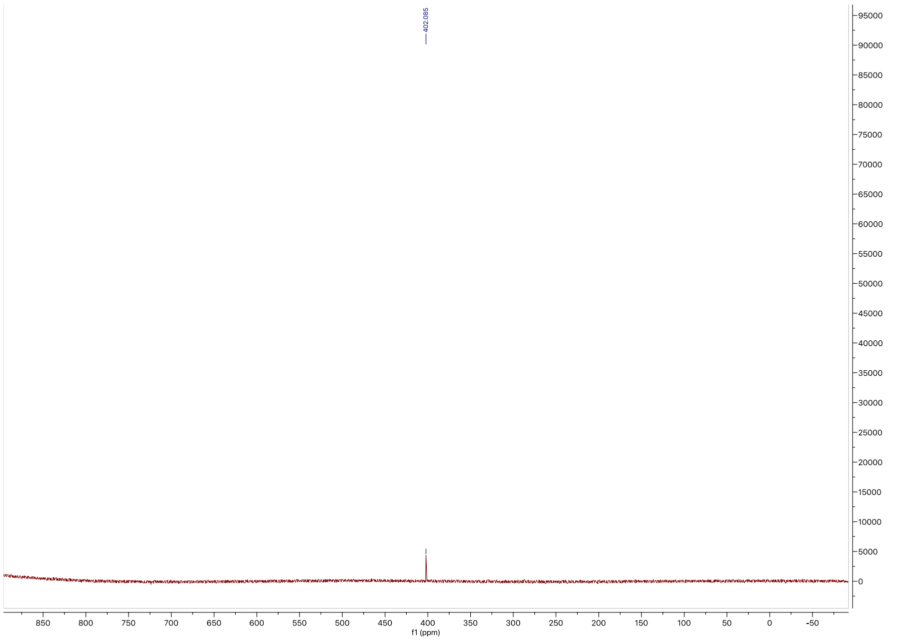

^{77}Se NMR of compound **9**

No	Code	Structure	MW	Purity
**10**	**PPK-33**	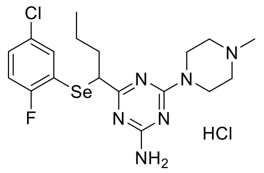	494.31	97.25%

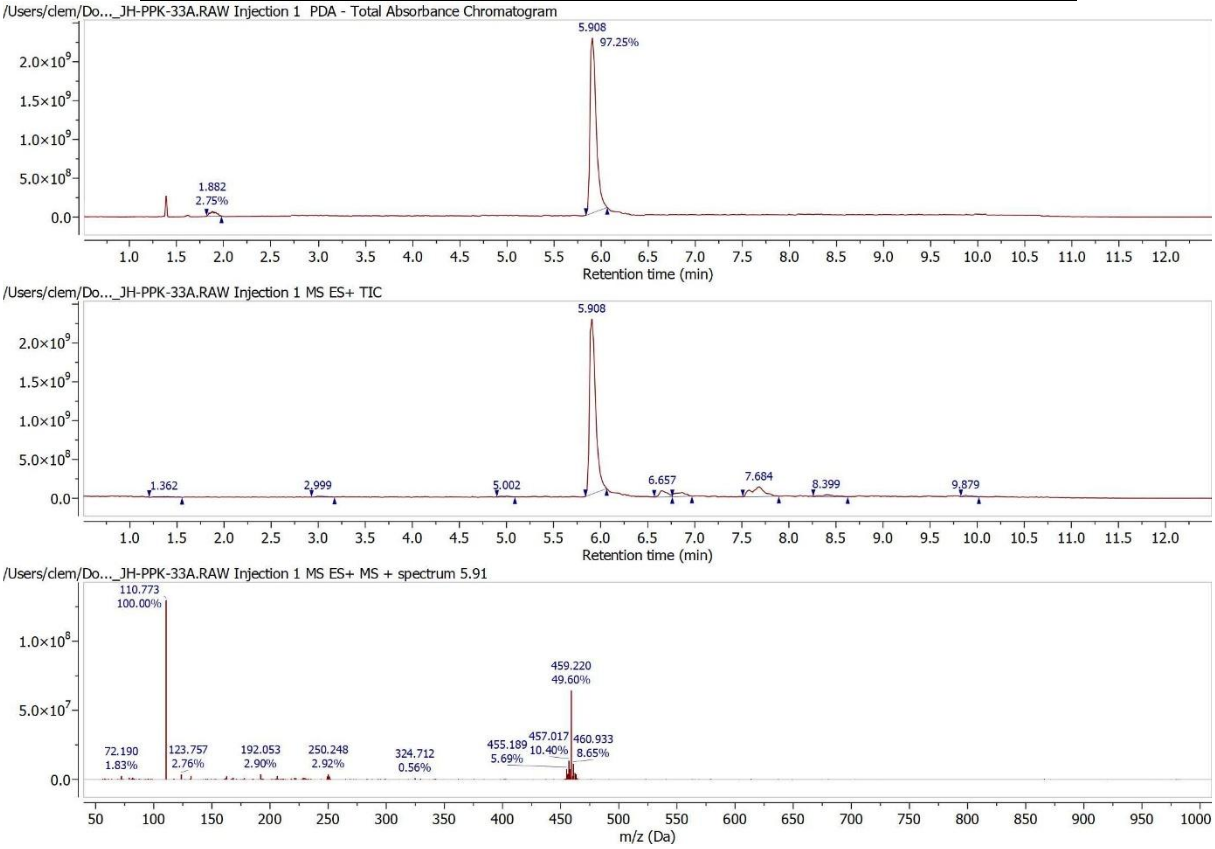

HPLC trace and mass spectrometry of compound **10**

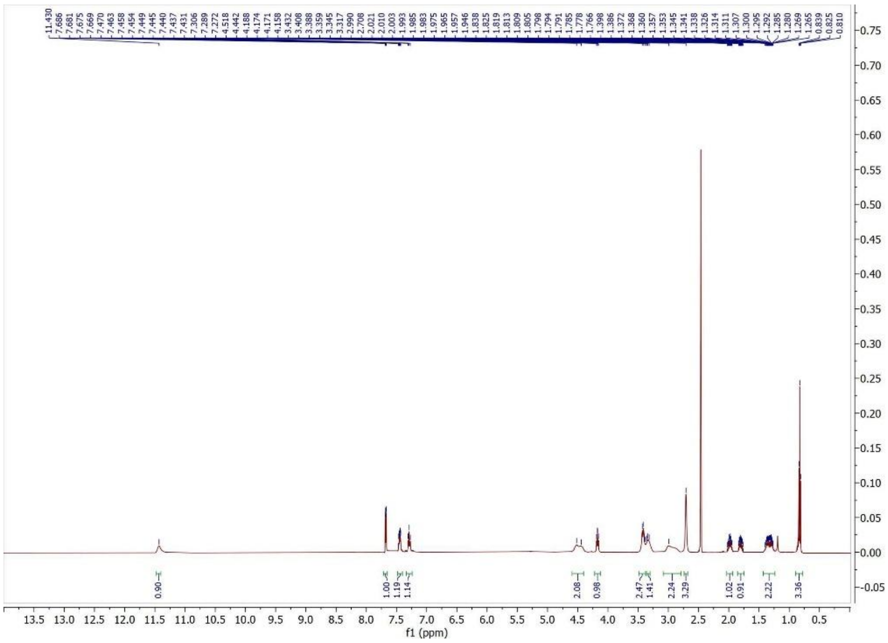

^{1}H NMR of compound **10**

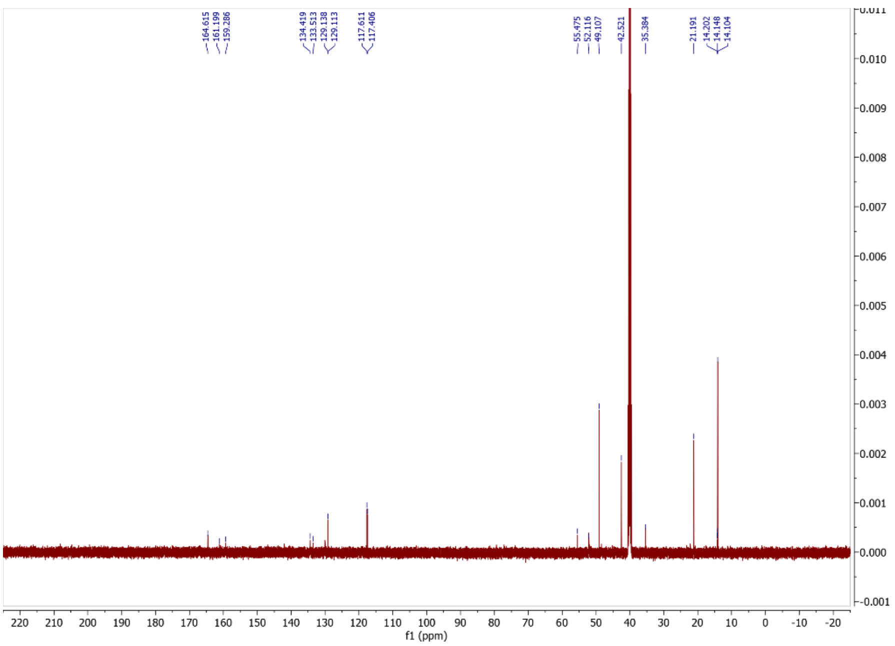

^{13}C NMR of compound **10**

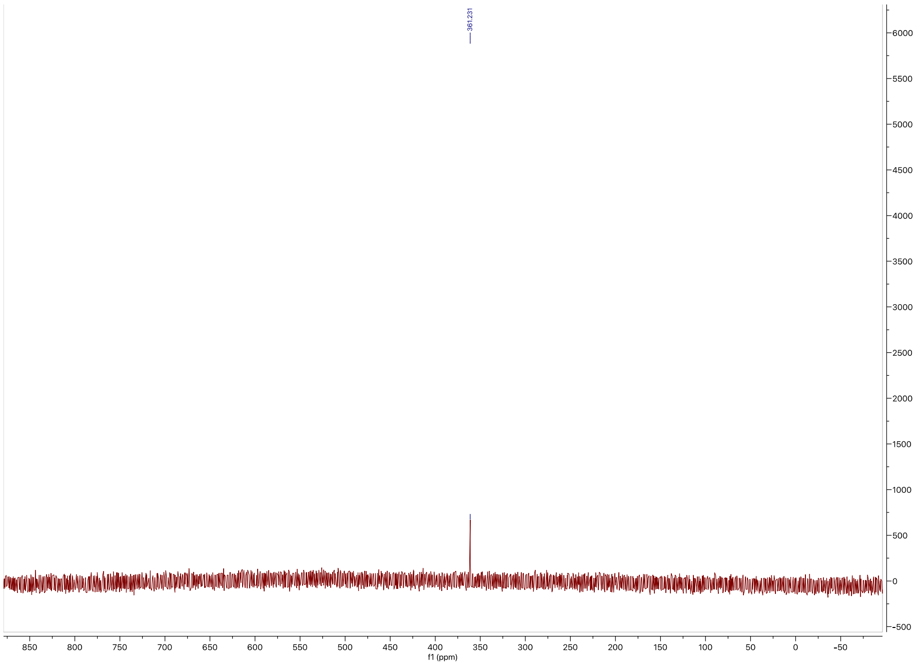

^{77}Se NMR of compound **10**

No	Code	Structure	MW	Purity
**11**	**JK-7**	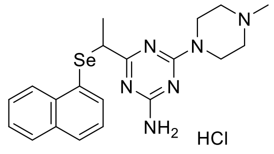	427.43	100%

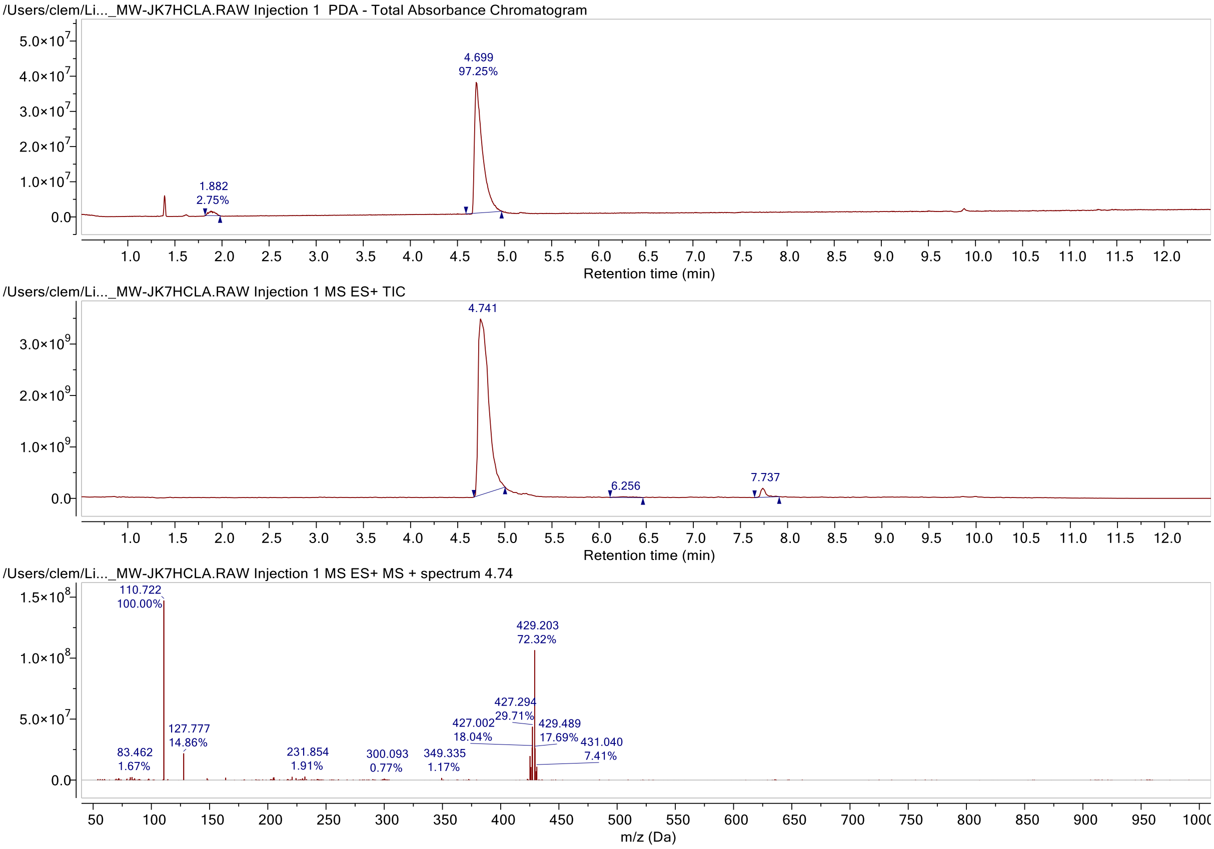

HPLC trace and mass spectrometry of compound **11**

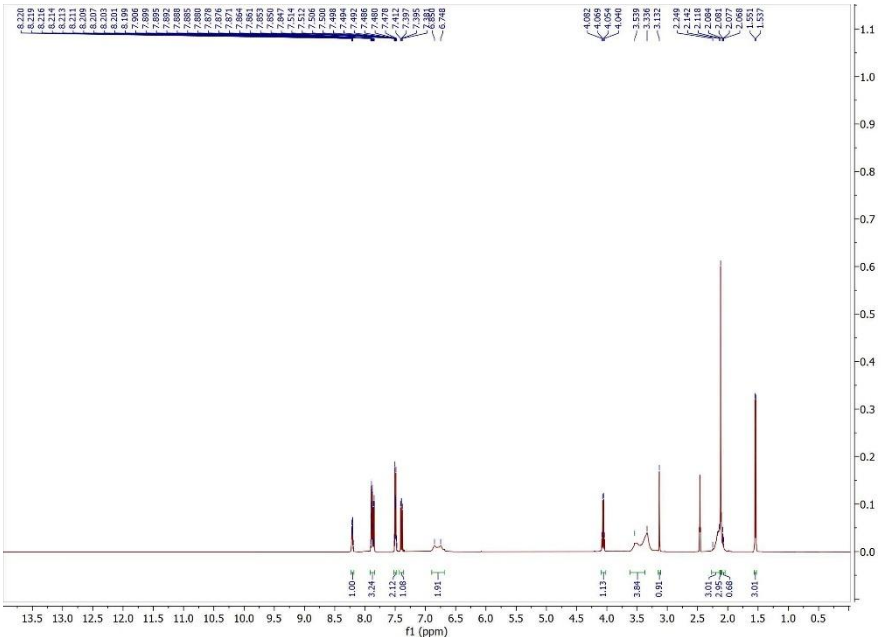

^{1}H NMR of compound **11**

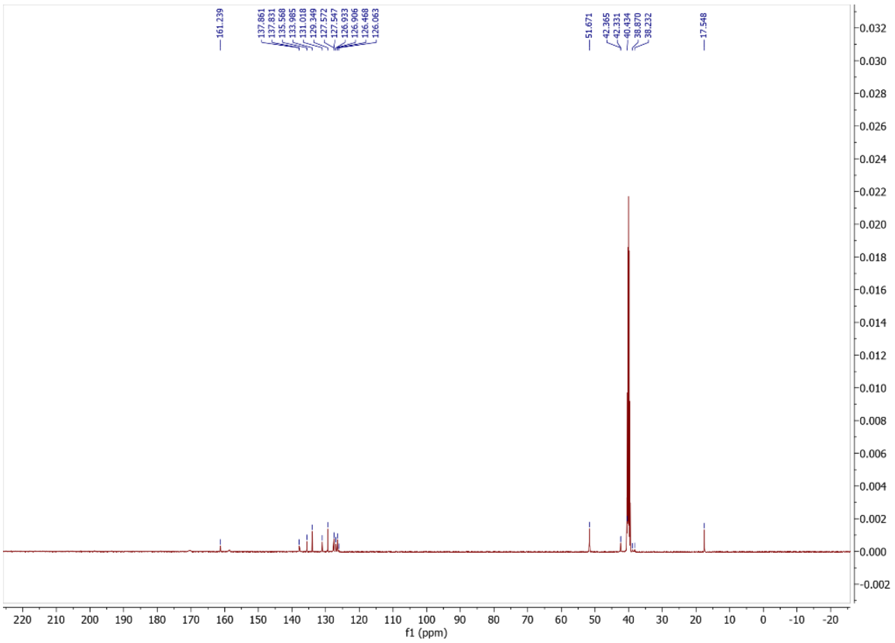

^{13}C NMR of compound **11**

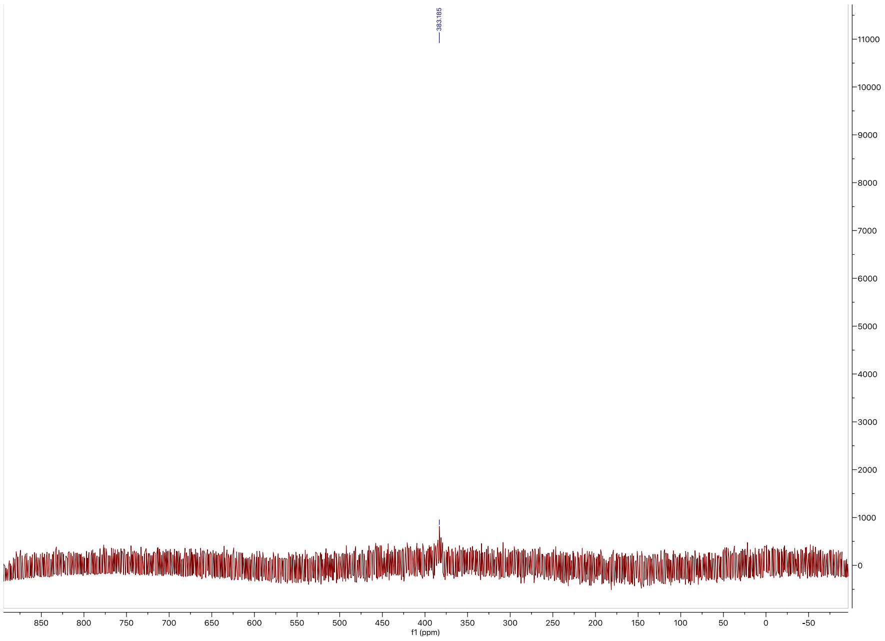

^{77}Se NMR of compound **11**

No	Cpd	Structure	MW	Purity
**12**	**JK-8**	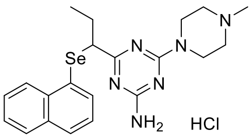	441.43	98.28%

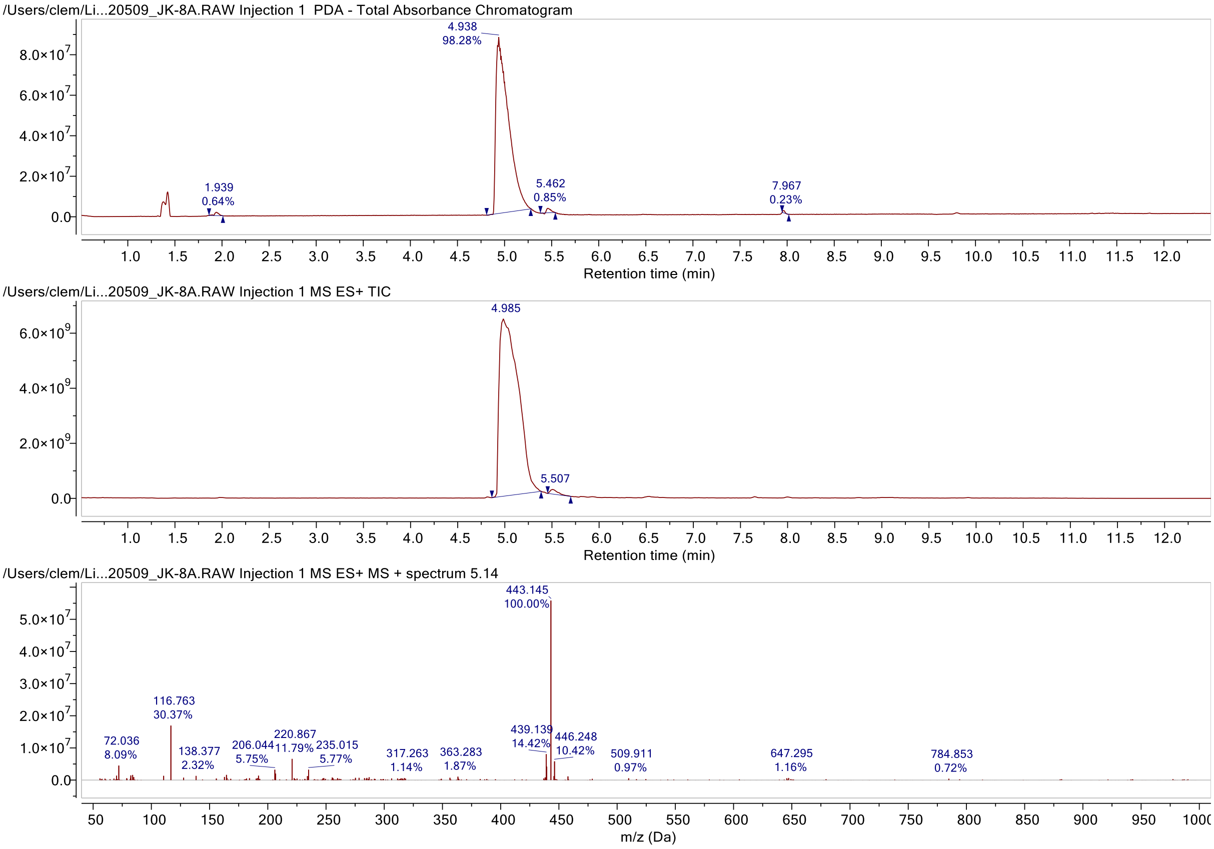

HPLC trace and mass spectrometry of compound **12**

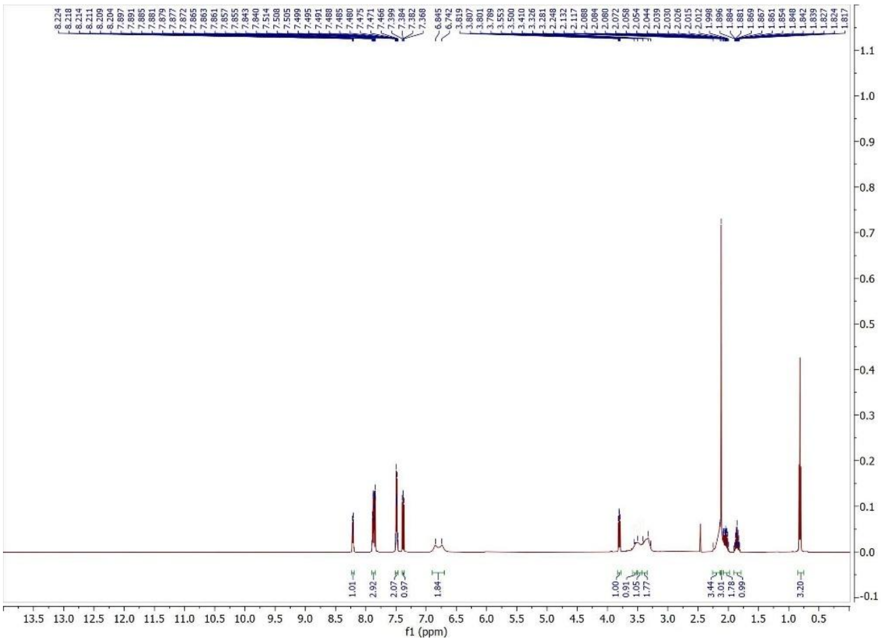

^{1}H NMR of compound **12**

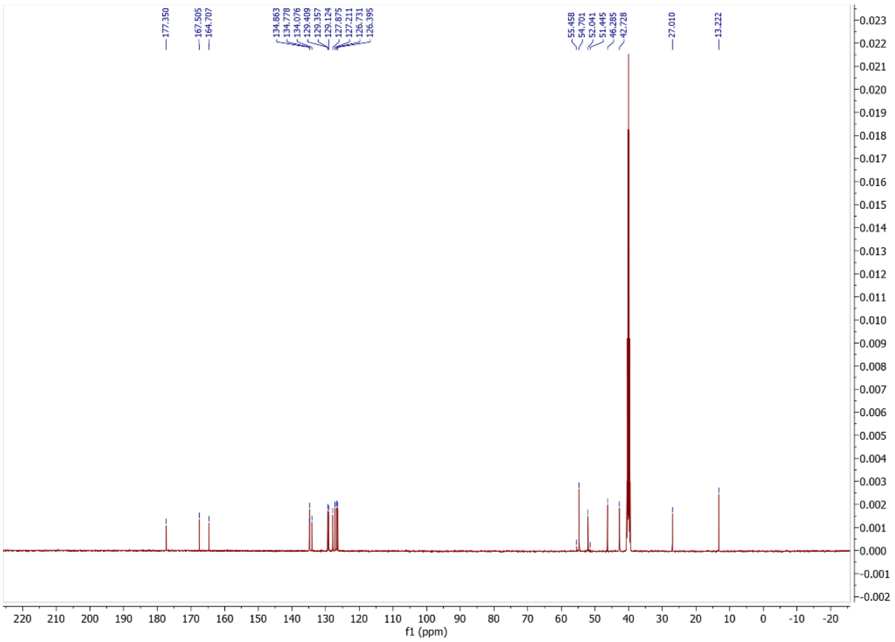

^{13}C NMR of compound **12**

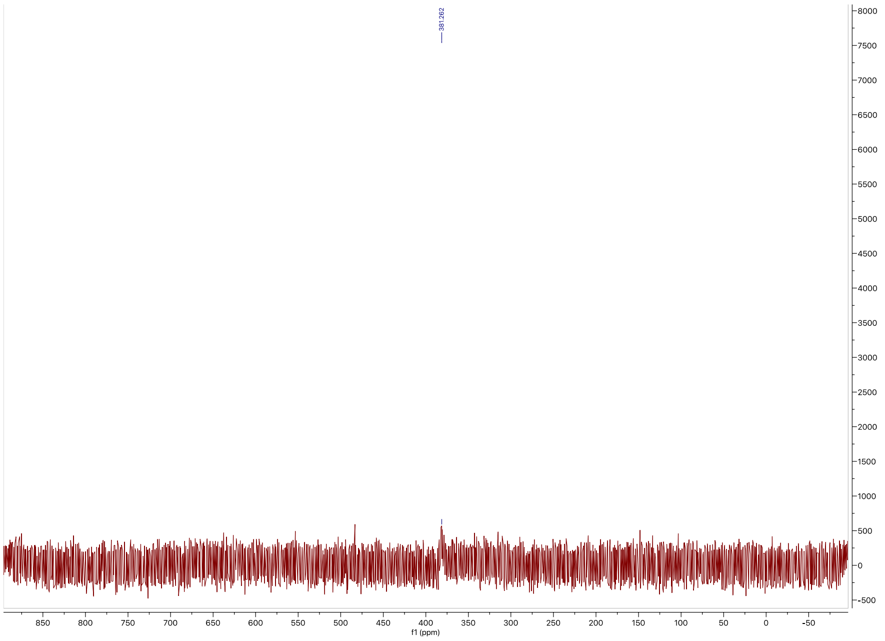

^{77}Se NMR of compound **12**

No	Code	Structure	MW	Purity
**13**	**JK-5**	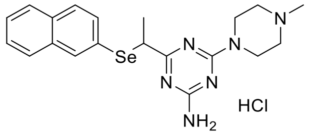	463.81	99.33%

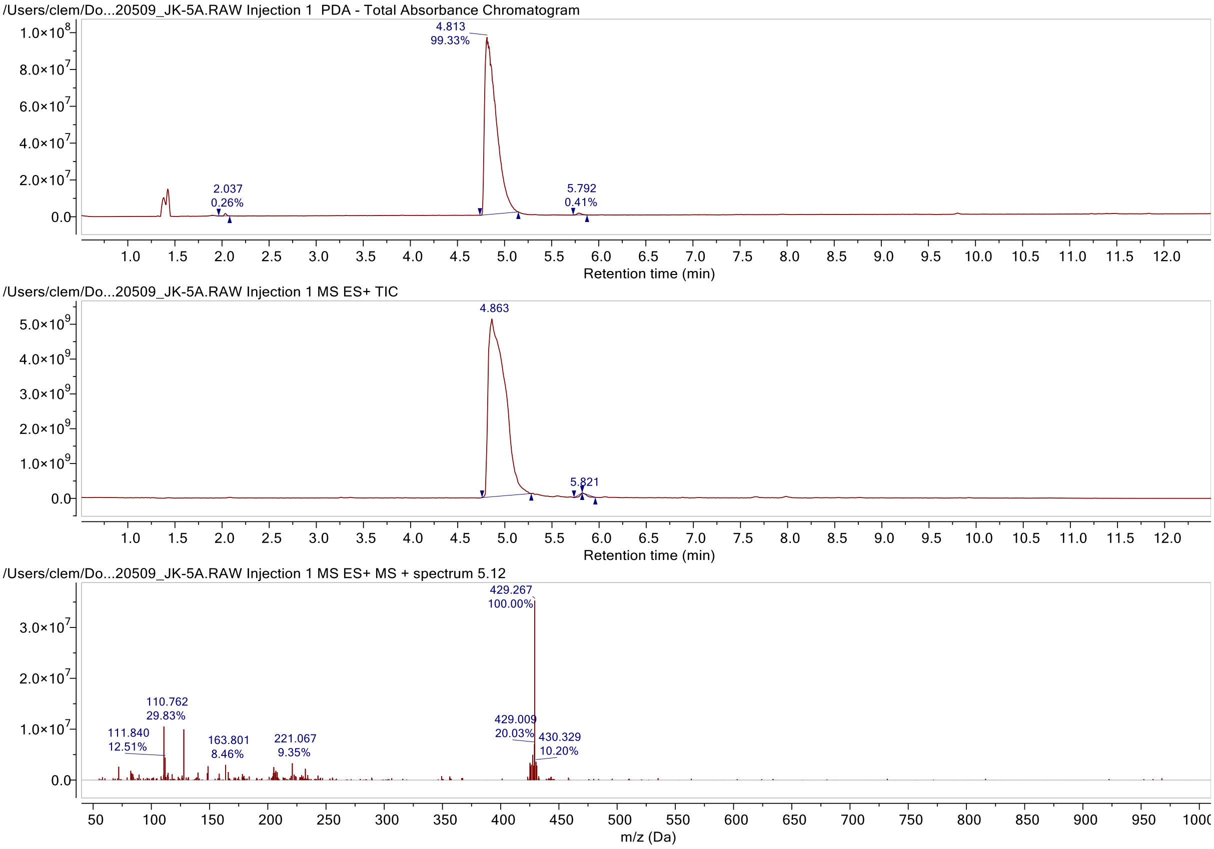

HPLC trace and mass spectrometry of compound **13**

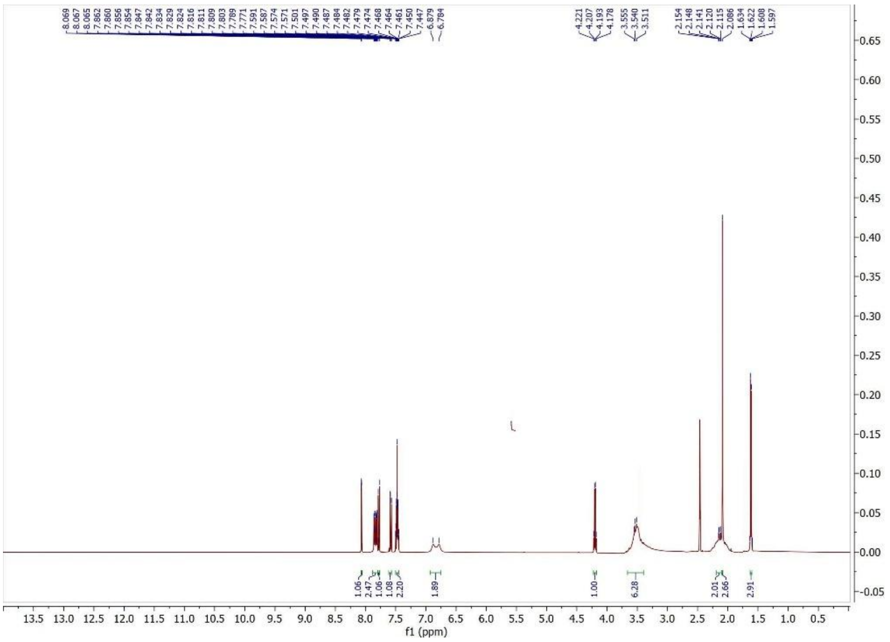

^{1}H NMR of compound **13**

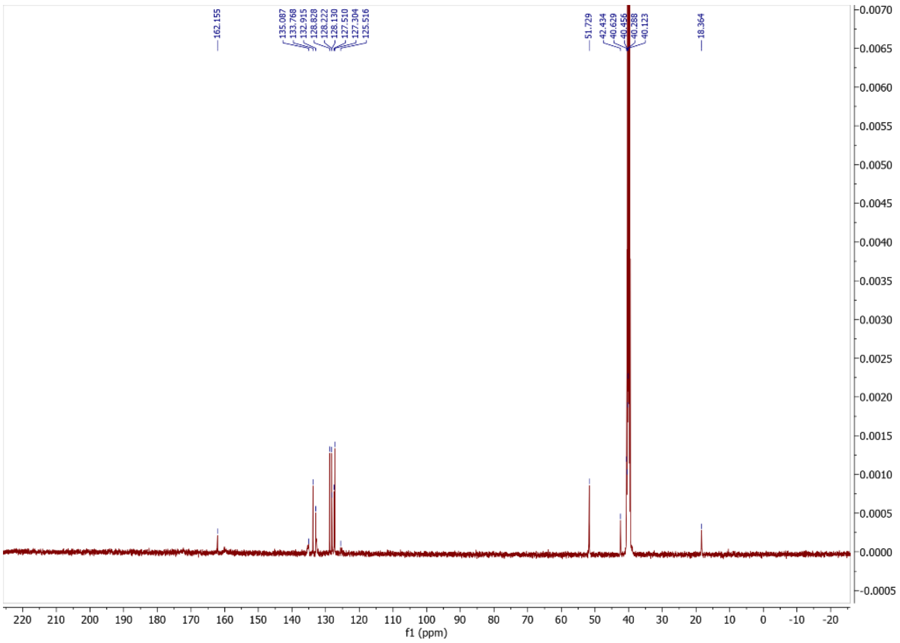

^{13}C NMR of compound **13**

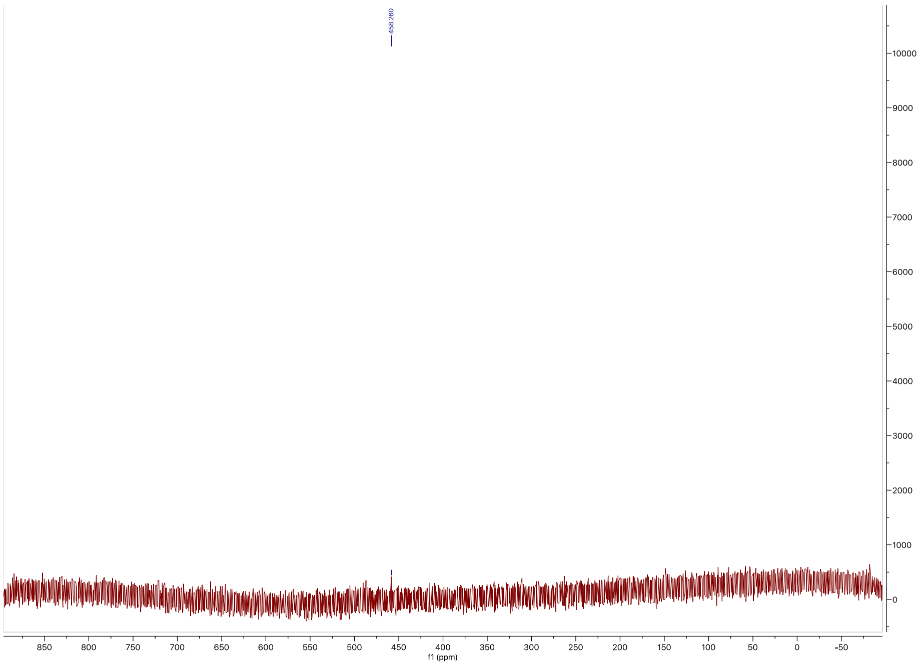

^{77}Se NMR of compound **13**

No	Code	Structure	MW	Purity
**14**	**JK-6**	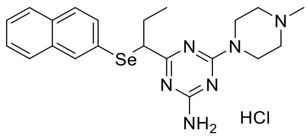	441.43	97.78%

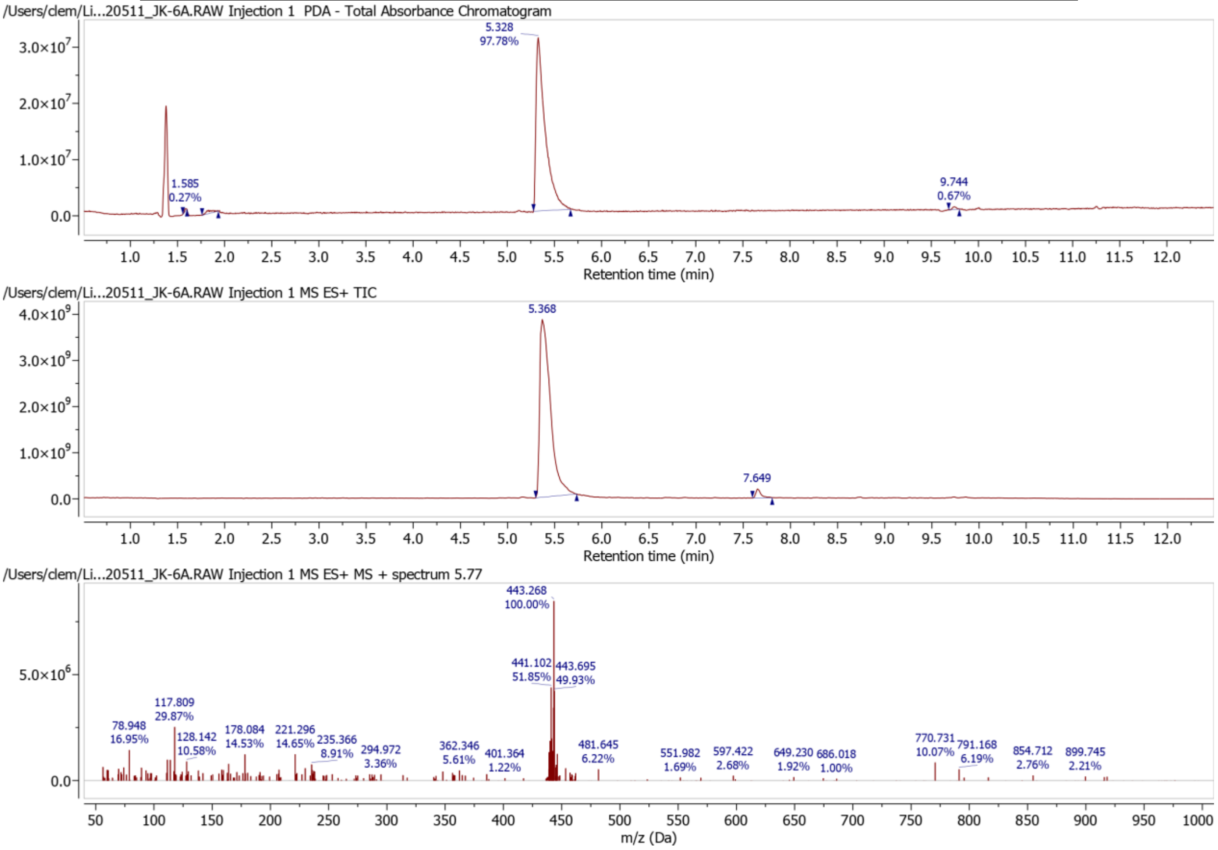

HPLC trace and mass spectrometry of compound **14**

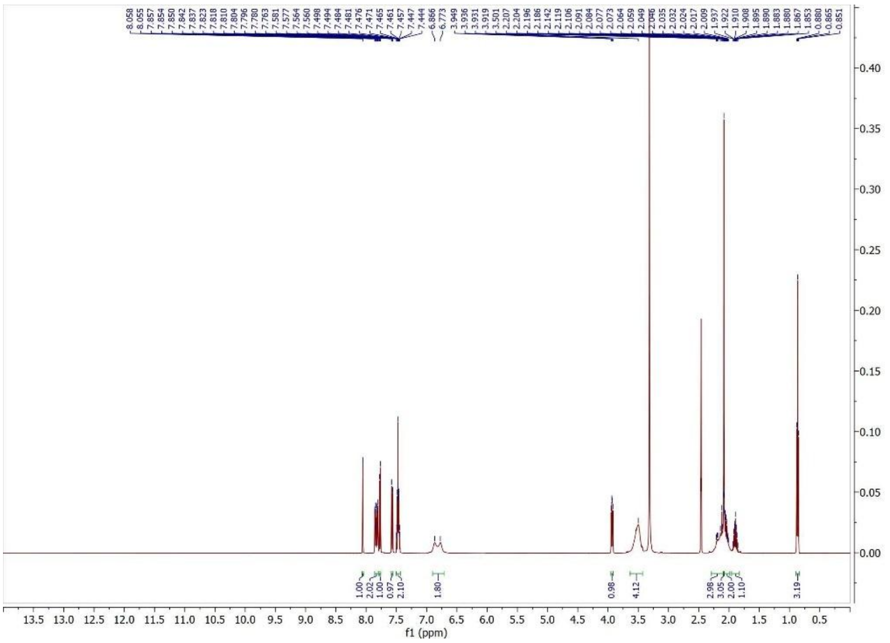

^{1}H NMR of compound **14**

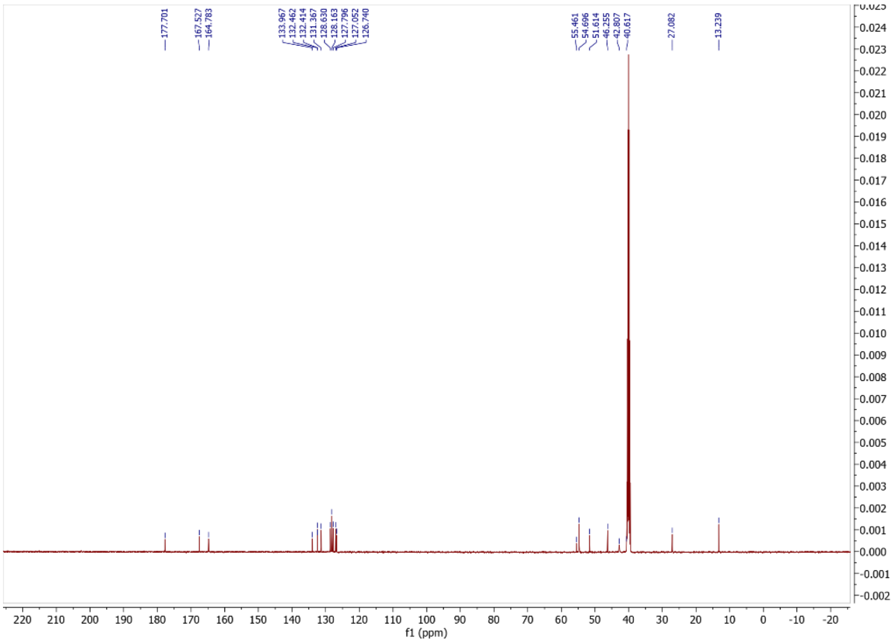

^{13}C NMR of compound **14**

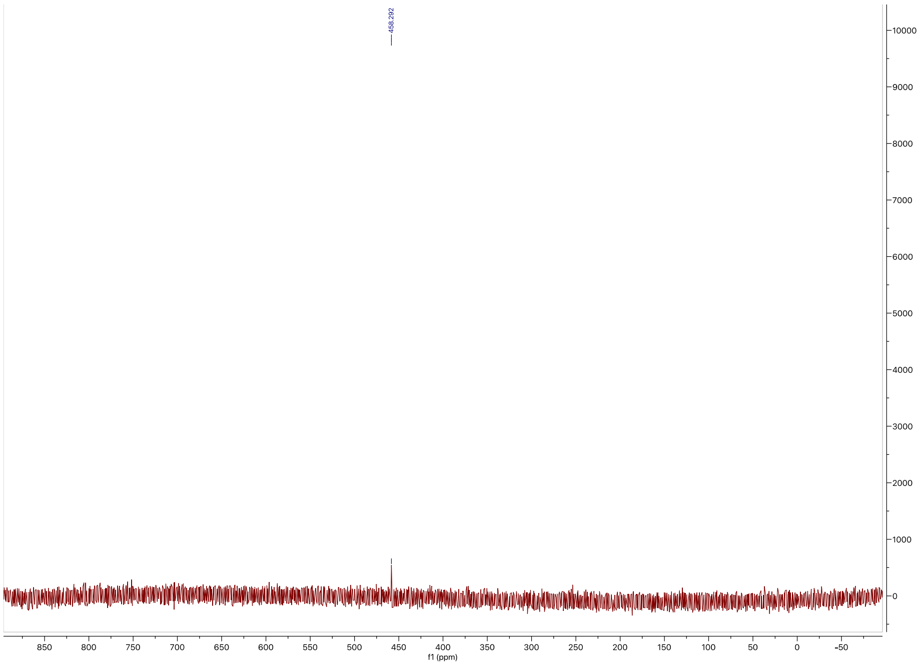

^{77}Se NMR of compound **14**

No	Code	Structure	MW	Purity
**15**	**PPK-32**	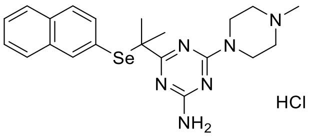	477.91	100%

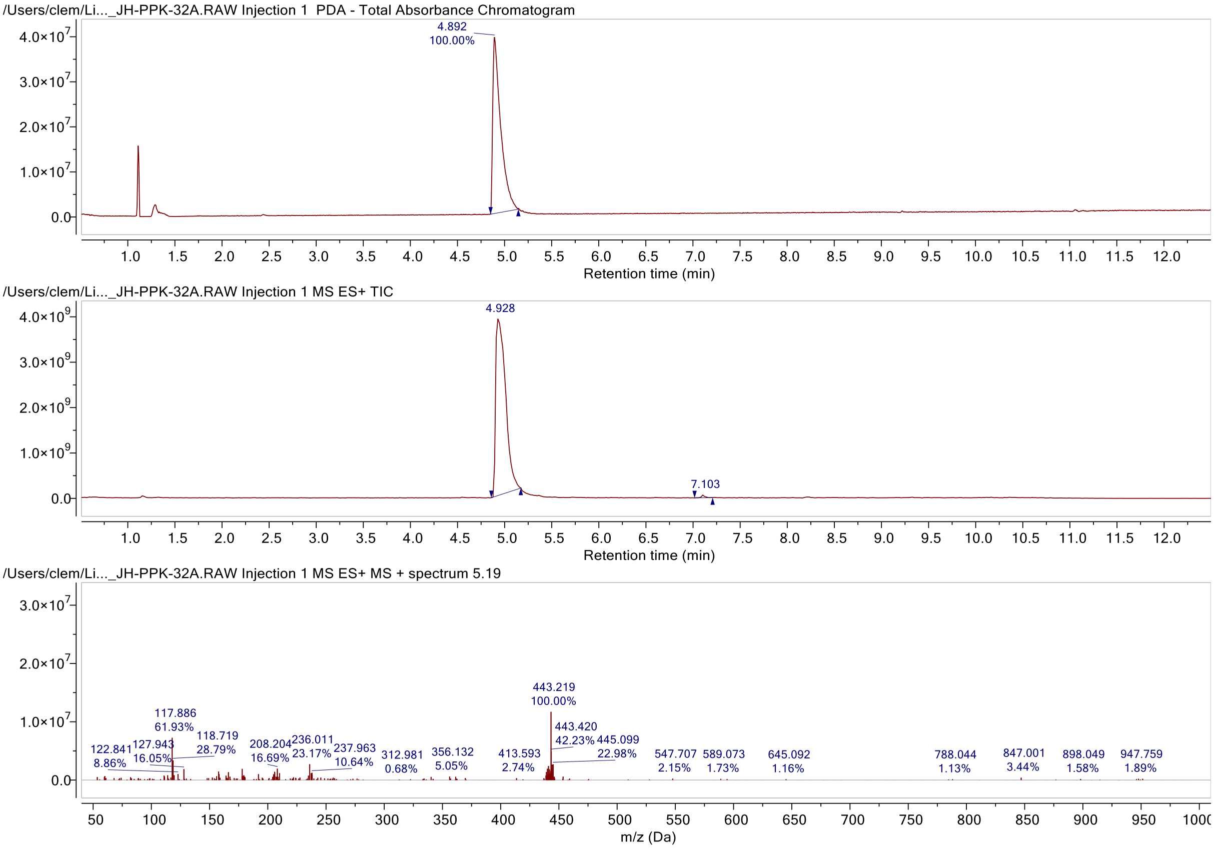

HPLC trace and mass spectrometry of compound **15**

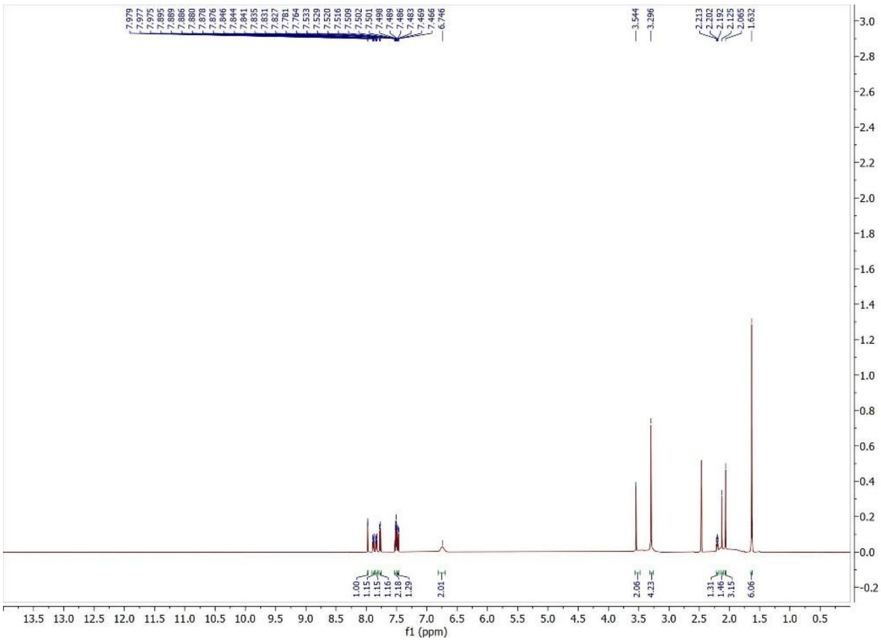

^{1}H NMR of compound **15**

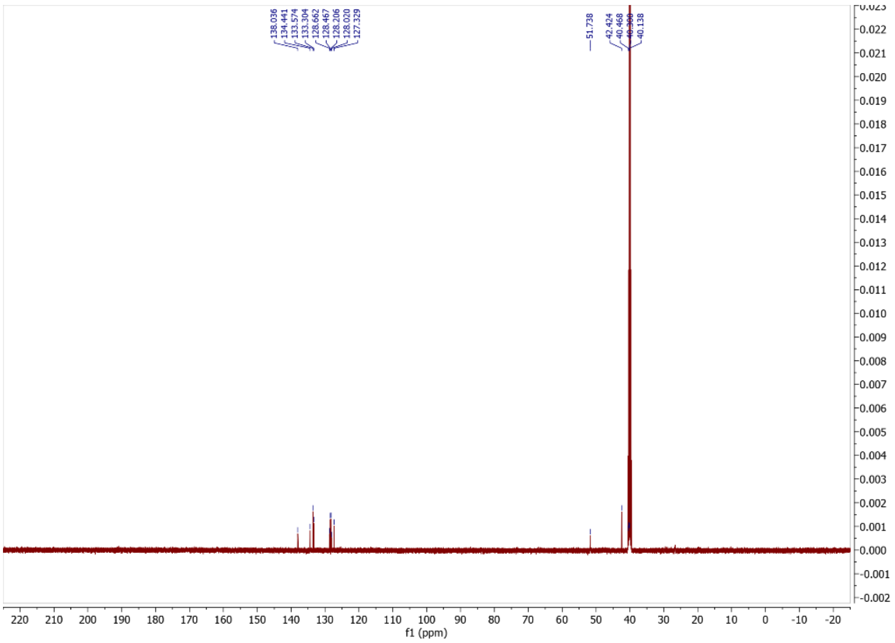

^{13}C NMR of compound **15**

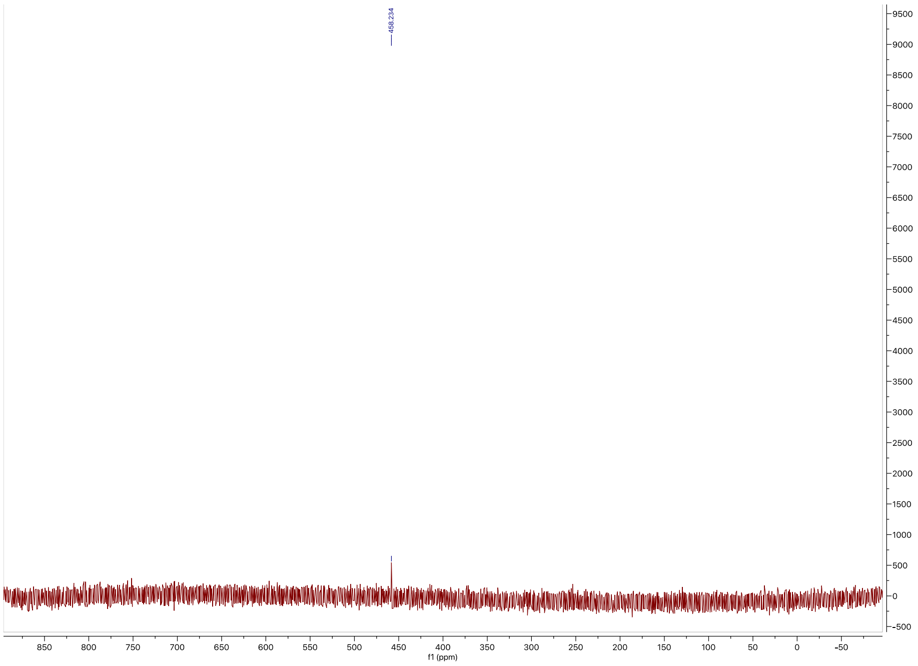

^{77}Se NMR of compound **15**

No	Code	Structure	MW	Purity
**17**	**WA-Se-10**	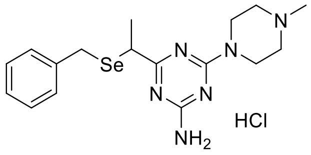	427.84	95.42%

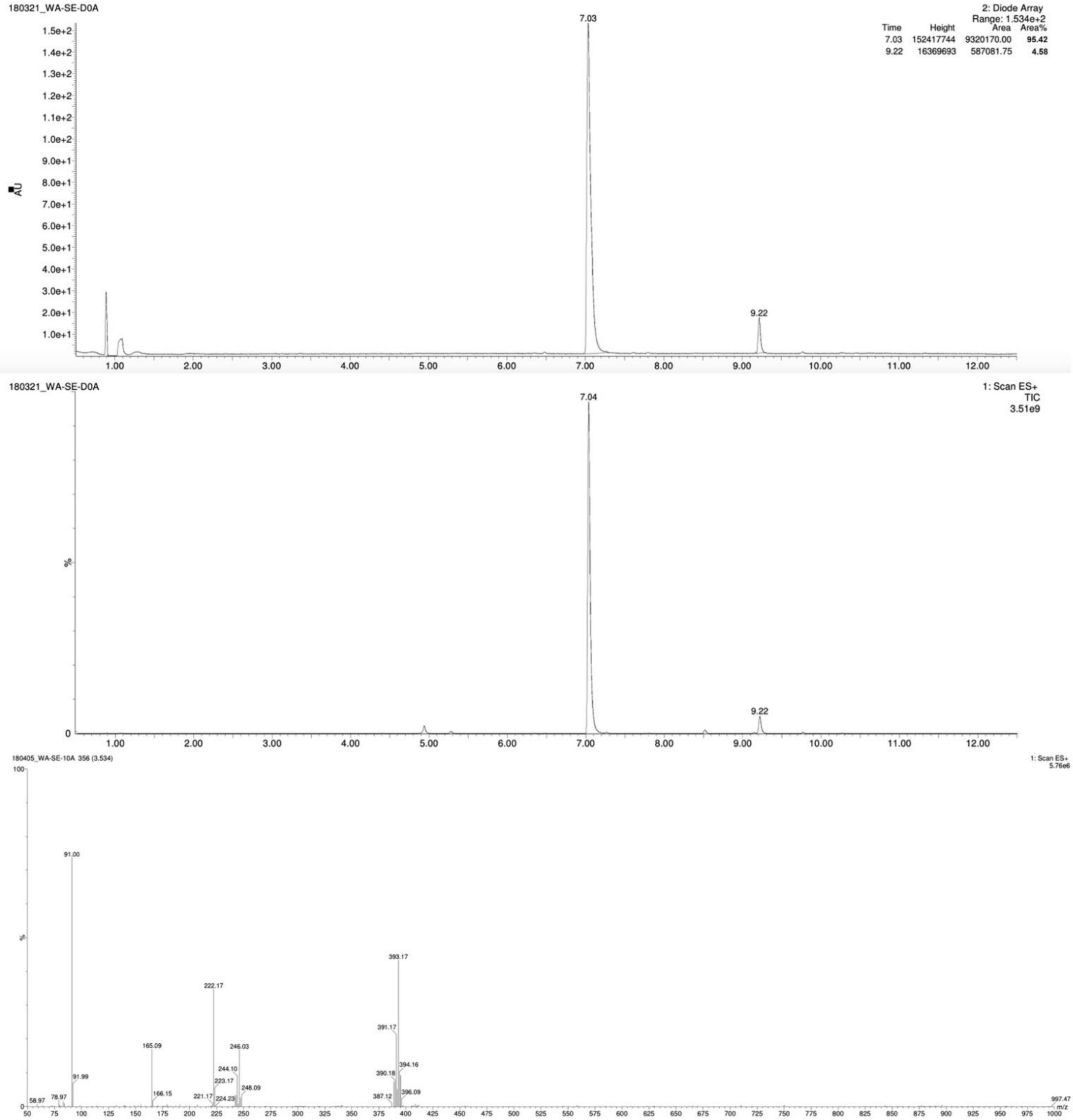

HPLC trace and mass spectrometry of compound **17**

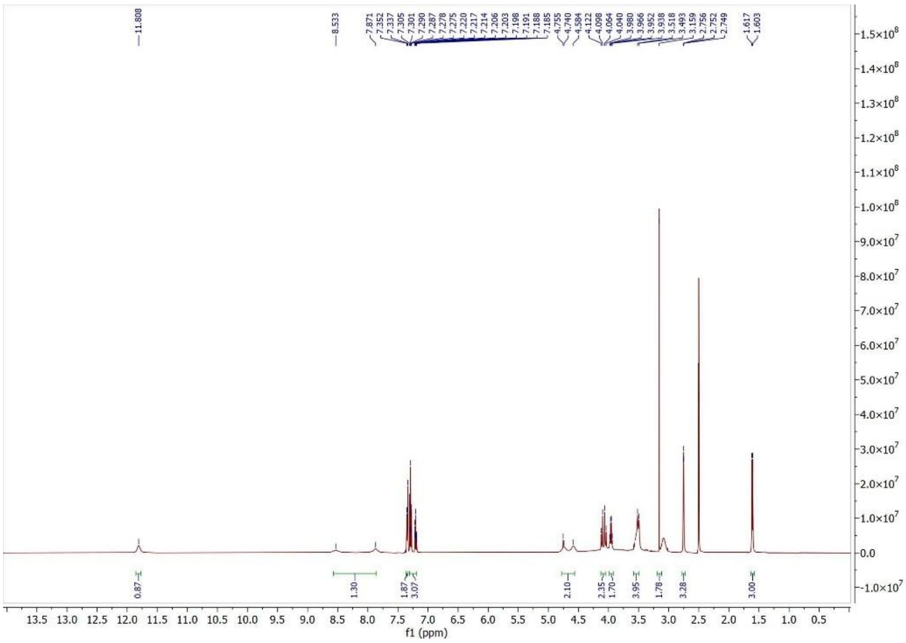

^{1}H NMR of compound **17**

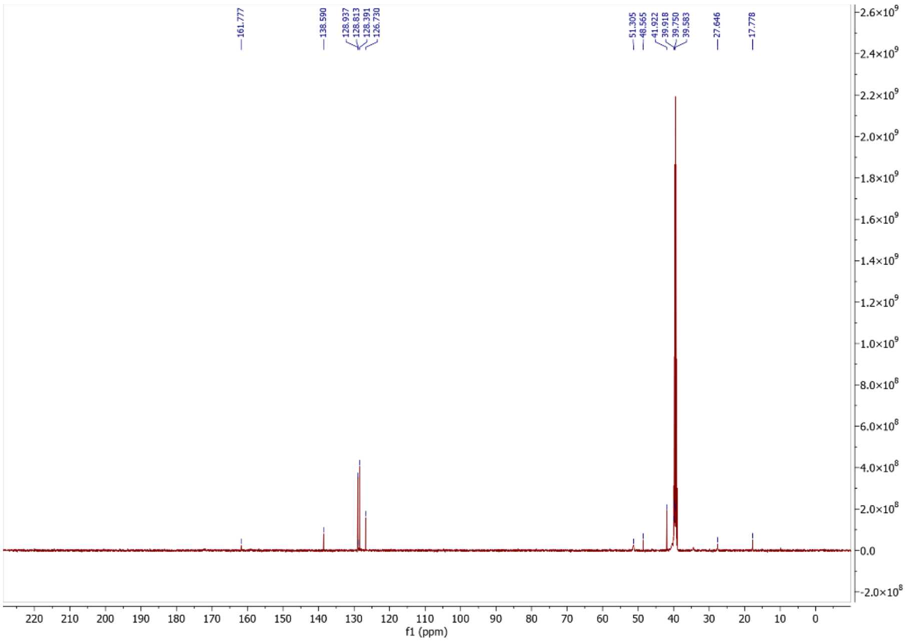

^{13}C NMR of compound **17**

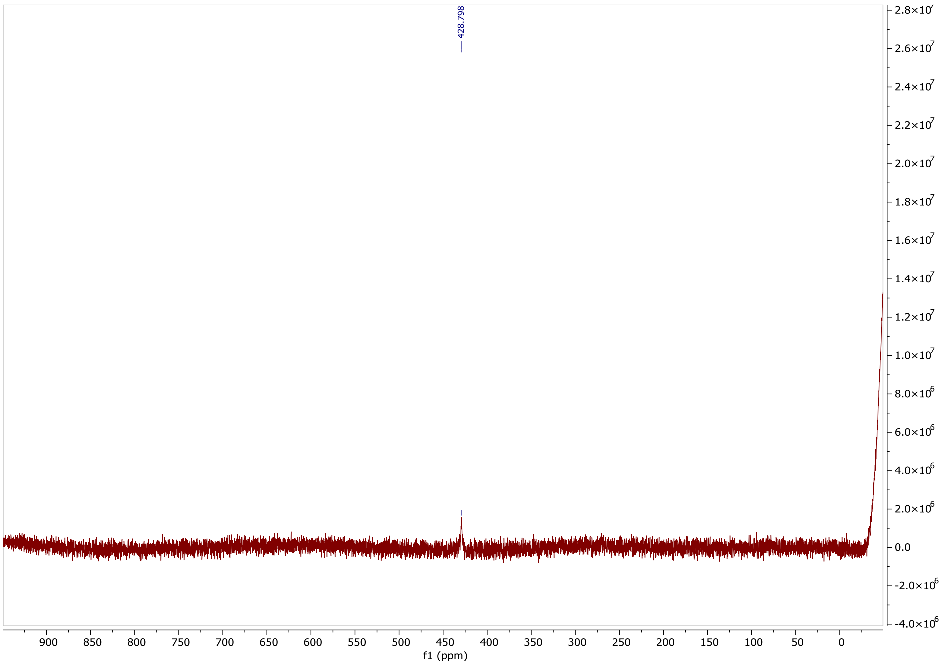

^{77}Se NMR of compound **17**

No	Code	Structure	MW	Purity
**15O**	**KC-9**	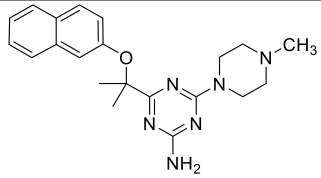	378,48	>99%

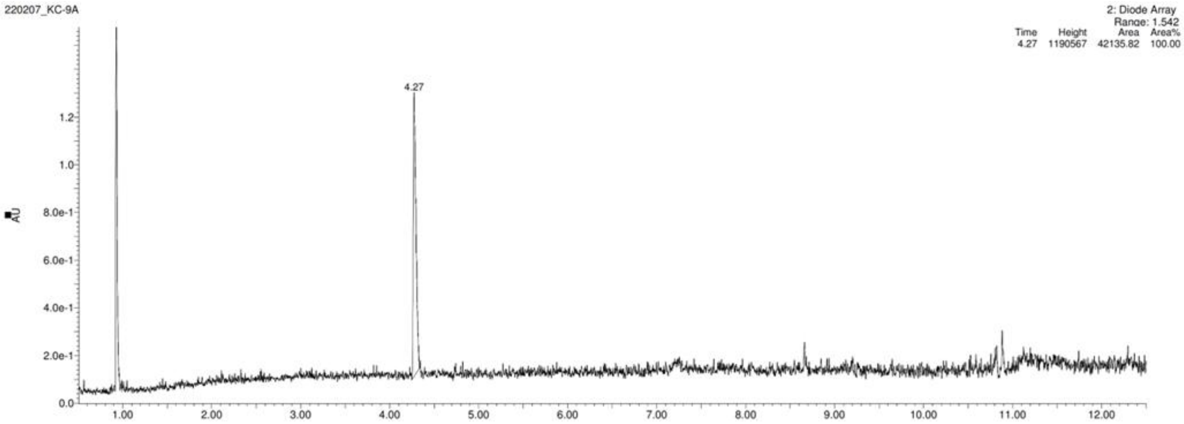

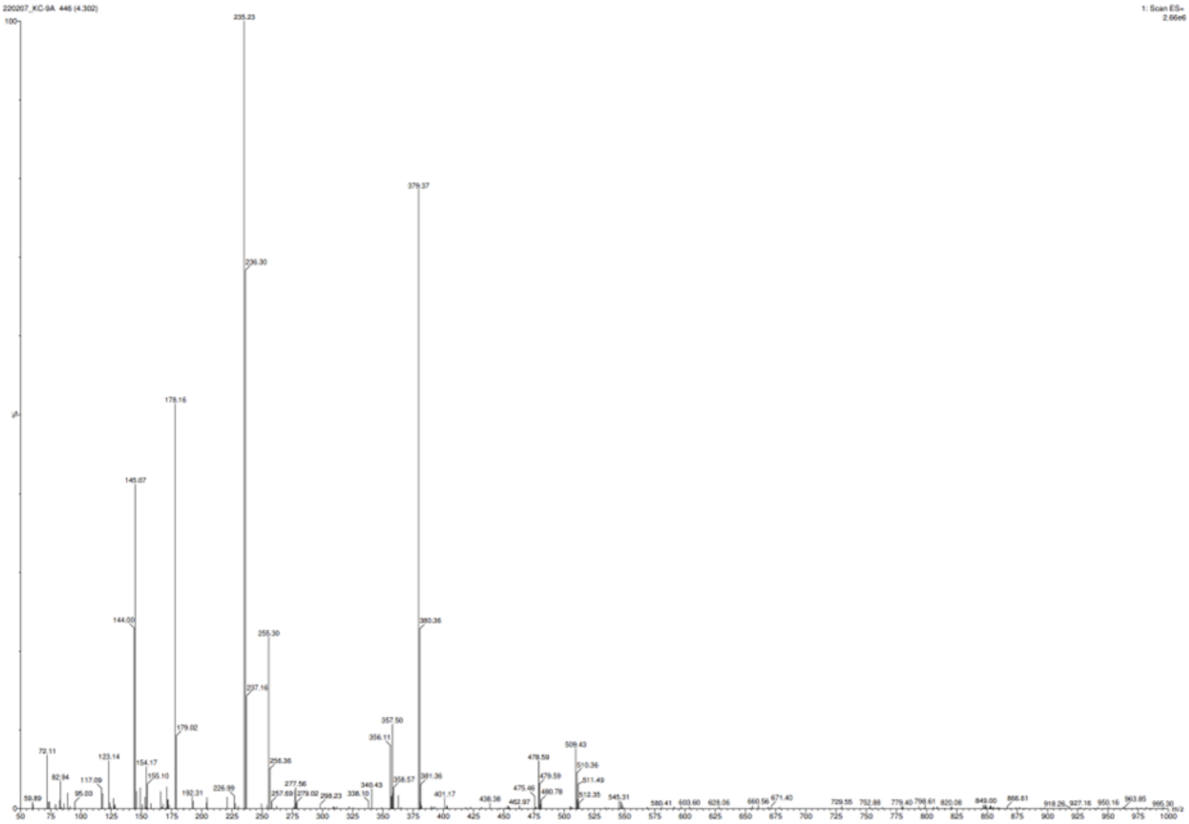

HPLC trace and mass spectrometry of compound **15O**

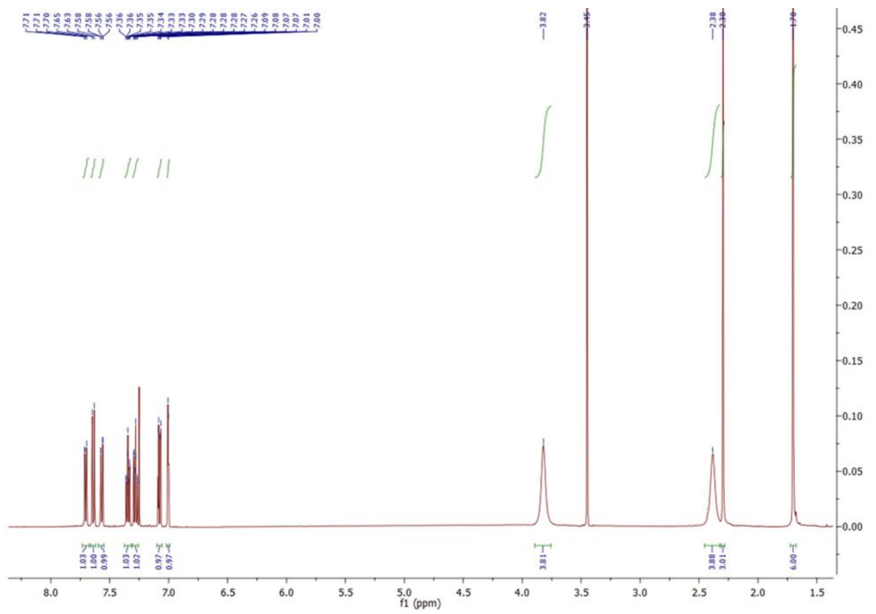

^{1}H NMR of compound **15O**

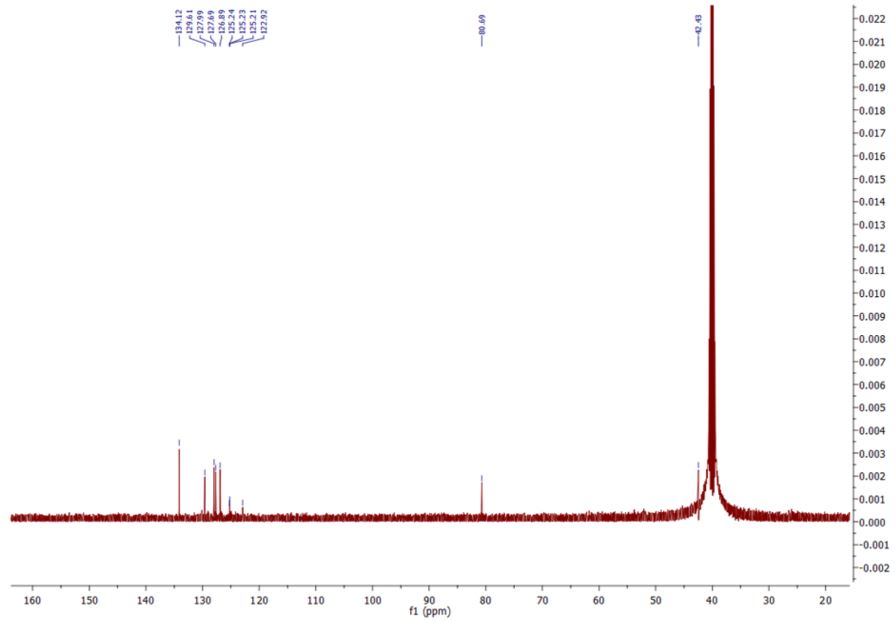

^{13}C NMR of compound **15O**

Table S1. Elemental analysis of final compounds 3, 6-15 and 17

Cmp	Calculated			Found		
	**C**	**H**	**N**	**C**	**H**	**N**
**3**	54.41	6.73	20.04	54.35	6.79	20.08
**6**	53.33	6.46	20.73	53.37	6.51	20.68
**7**	54.41	6.73	20.04	54.39	6.75	20.01
**8**	55.42	6.98	19.39	55.45	6.94	19.33
**9**	54.41	6.73	20.04	54.40	6.71	20.08
**10**	47.22	5.28	18.36	47.25	5.29	18.32
**11**	56.20	5.66	19.66	56.23	5.61	19.70
**12**	57.14	5.94	19.04	57.15	5.98	19.01
**13**	56.20	5.66	19.66	56.21	5.65	19.61
**14**	57.14	5.94	19.04	57.17	5.91	19.08
**15**	57.14	5.94	19.04	57.15	5.97	19.07
**17**	52.17	6.18	21.47	52.15	6.21	21.43

Table S2. Structures of intermediate compounds 31-42

No	Code	Structure	Aspect	Yield	For Final
**31**	**MR-01B/PP5**	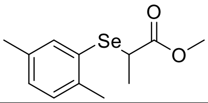	Yellowish oil	55.3%	**6**
**32**	**MR-02B/PP6**	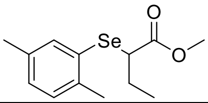	Yellowish oil	49.7%	**7**
**33**	**WD2E**	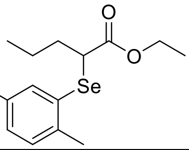	Yellowish oil	44.3%	**8**
**34**	**WD1E**	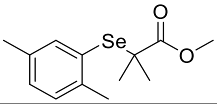	Yellowish oil	61.2%	**9**
**35**	**PP-11**	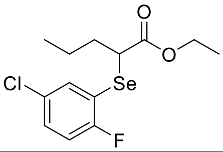	Yellowish oil	55.6 %	**10**
**36**	**JKE-7/PP-8**	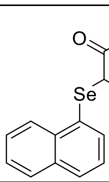	Yellowish oil	48.3%	**11**
**37**	**JKE-8/PP-9**	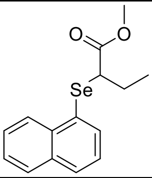	Yellowish oil	37.7%	**12**
**38**	**JKE-5/PP-7**	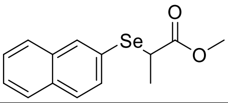	Yellowish oil	22.0%	**13**
**39**	**JKE-6/PP10**	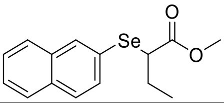	Yellowish oil	43.9%	**14**
**40**	**PP-3**	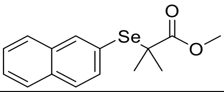	Yellowish oil	53.2%	**15**
**42**	**WA-Se10E**	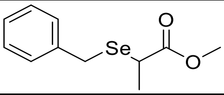	Yellowish oil	85.0%	**17**

Figures S1-S3 additional data of Molecular Modelling

I) II) III)

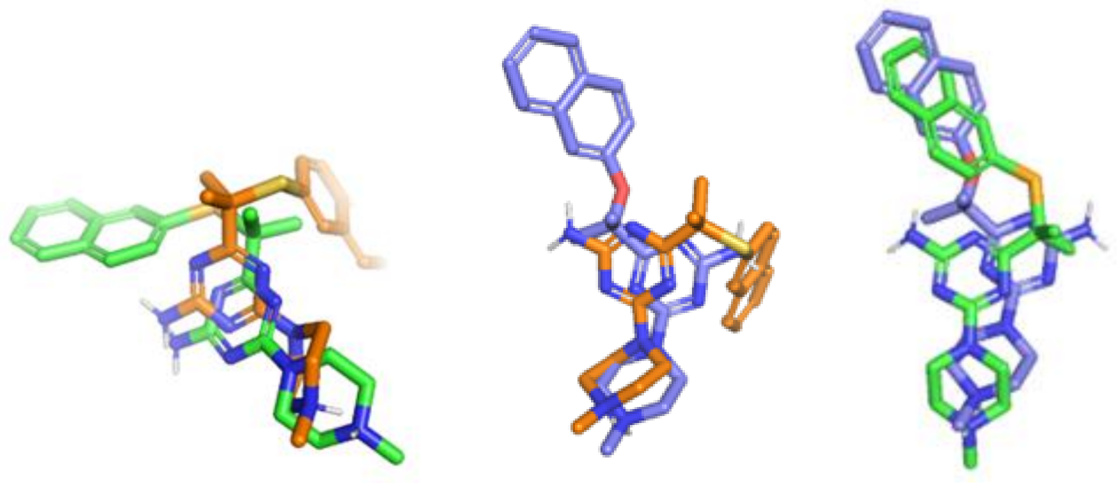

Figure S1 The pair overlapping for active conformations of compounds **15O**, **15S** and **15**: I) **15S** and **15**; II) **15O** and **15S**; III) **15O** and **15**

I)

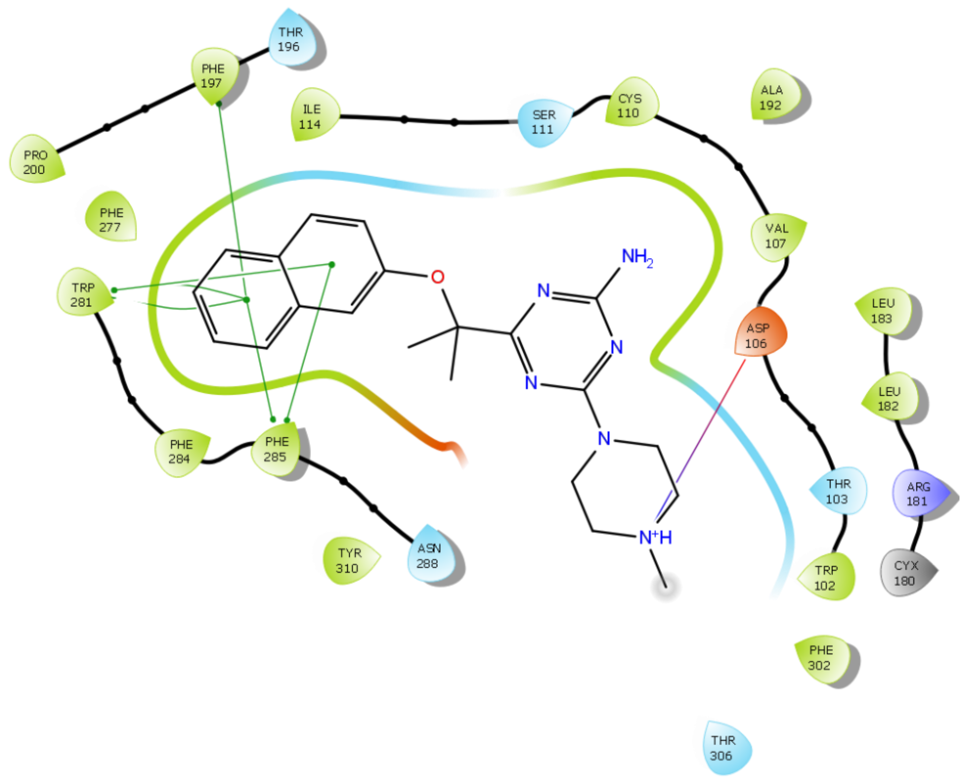

II)

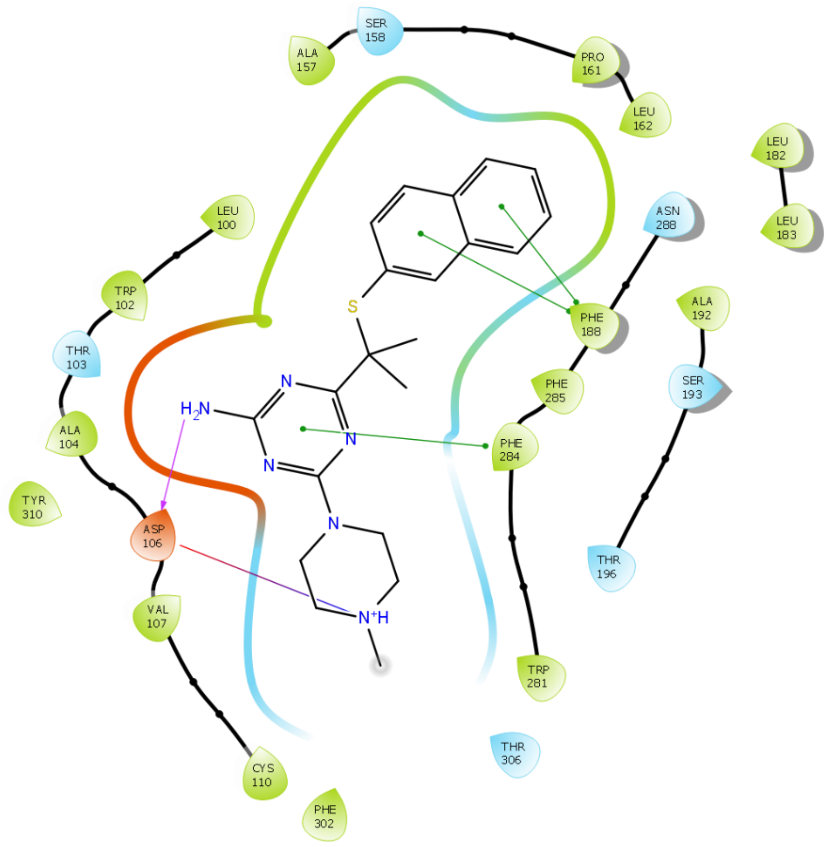

III)

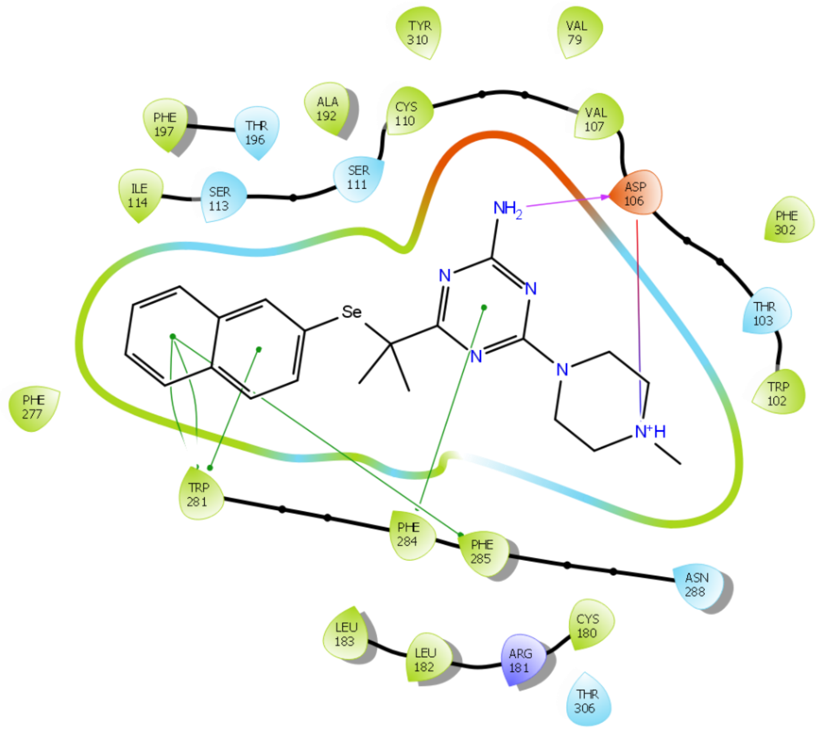

Figure S2. Ligand-protein interaction diagrams obtained for compounds: I) **15O,** II)**15S**, and III) **15** in docking to 5-HT$_6$R.

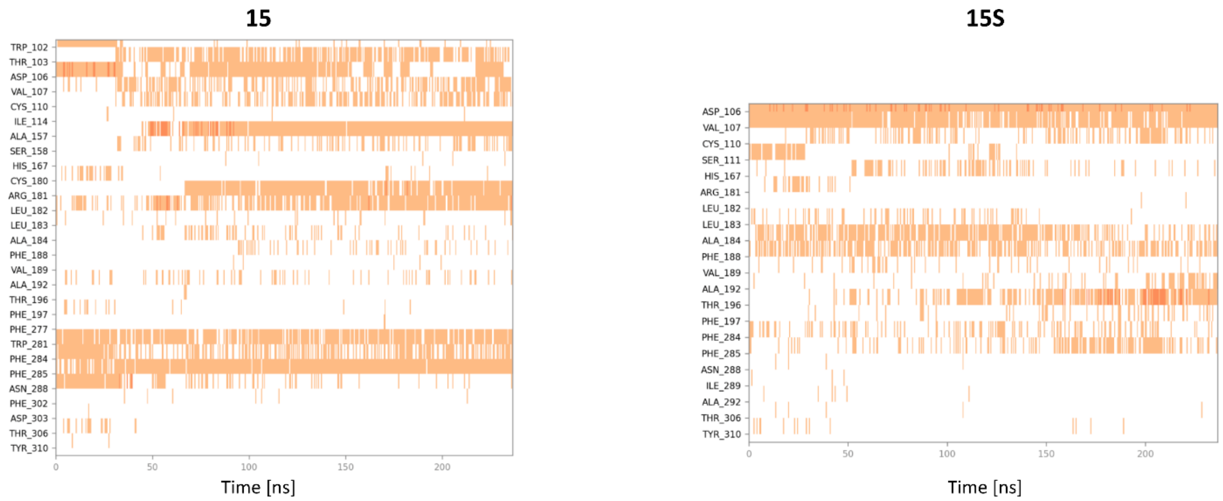

Figure S3. Ligand-protein contacts observed during MD simulations for compounds **15** and **15S**.

Functional bioassays for 5-HT$_6$ receptor

Five compounds were selected for the functional bioassays that were conducted in accordance with the following procedures. Compounds (**11-14**) were examined on 5-HT$_6$R using their ability to inhibit cAMP production induced by 1 µM (EC80) 5-carboxamidotryptamine (5-CT). The cAMP level was measured in 1321N1 cells expressing the h5-HT$_6$R (PerkinElmer, #ES-316-CF). According to the manufacturer's instructions, total cAMP was measured using the LANCE cAMP detection kit (PerkinElmer, #TRF0263). Cells were incubated with a mixture of compounds for 30 min at room temperature (RT) in a white polystyrene OptiPlate-384 (PerkinElmer, #6007299) microplate. After incubation, the reaction cells were lysed by adding 10 µL of cAMP detection buffer, including Eu-cAMP tracer and ULight-anti-cAMP working solution. The plate was incubated at RT for 1 h before measuring the signal with a Tecan multimode plate reader (Infinite M1000 Pro). Compounds were tested in triplicate at eight concentrations in the range from 10−11 to 10−4 M. K_b constants were calculated from Cheng−Prusoff equation adapted to functional assays. Compound **15** was dissolved in dimethyl sulfoxide (DMSO) at a concentration of 10 mM. Serial dilutions were prepared in a 96-well microplate in assay buffer, and 8 concentrations were tested. For the 5-HT$_6$, adenylyl cyclase activity was monitored using cryopreserved 1321N1 cells expressing the human serotonin 5-HT$_6$ receptor (Perkin Elmer, USA). Thawed cells were resuspended in stimulation buffer (HBSS, 5 mM HEPES, 0.5 IBMX, and 0.1% BSA at pH 7.4) at $2x10^5$ cells/ml. The same cell suspension volume (10 µl) was added to the tested compound. Samples were loaded onto a white opaque half area 96-well microplate. The antagonist response experiment was performed with 22 nM serotonin as the reference agonist for the 5-HT$_6$ receptor. The agonist and antagonist were added simultaneously. Cell stimulation was performed for 30 minutes at room temperature. After incubation, cAMP measurements were performed with homogeneous TR-FRET immunoassay using the LANCE Ultra cAMP kit (PerkinElmer, USA). 10 µl of EucAMP Tracer Working Solution and 10 µl of ULight-anti-cAMP Tracer Working Solution were added, mixed, and incubated for 1 h. The TR-FRET signal was read on an EnVision microplate reader (PerkinElmer, USA). IC_{50} and EC_{50} were determined by nonlinear regression analysis using GraphPad Prism 7.0 software.

Figure S4 Dose−response curves from functional *in vitro* assay for cAMP response of compounds 11-15 and references (Olanzapine, SB752457)

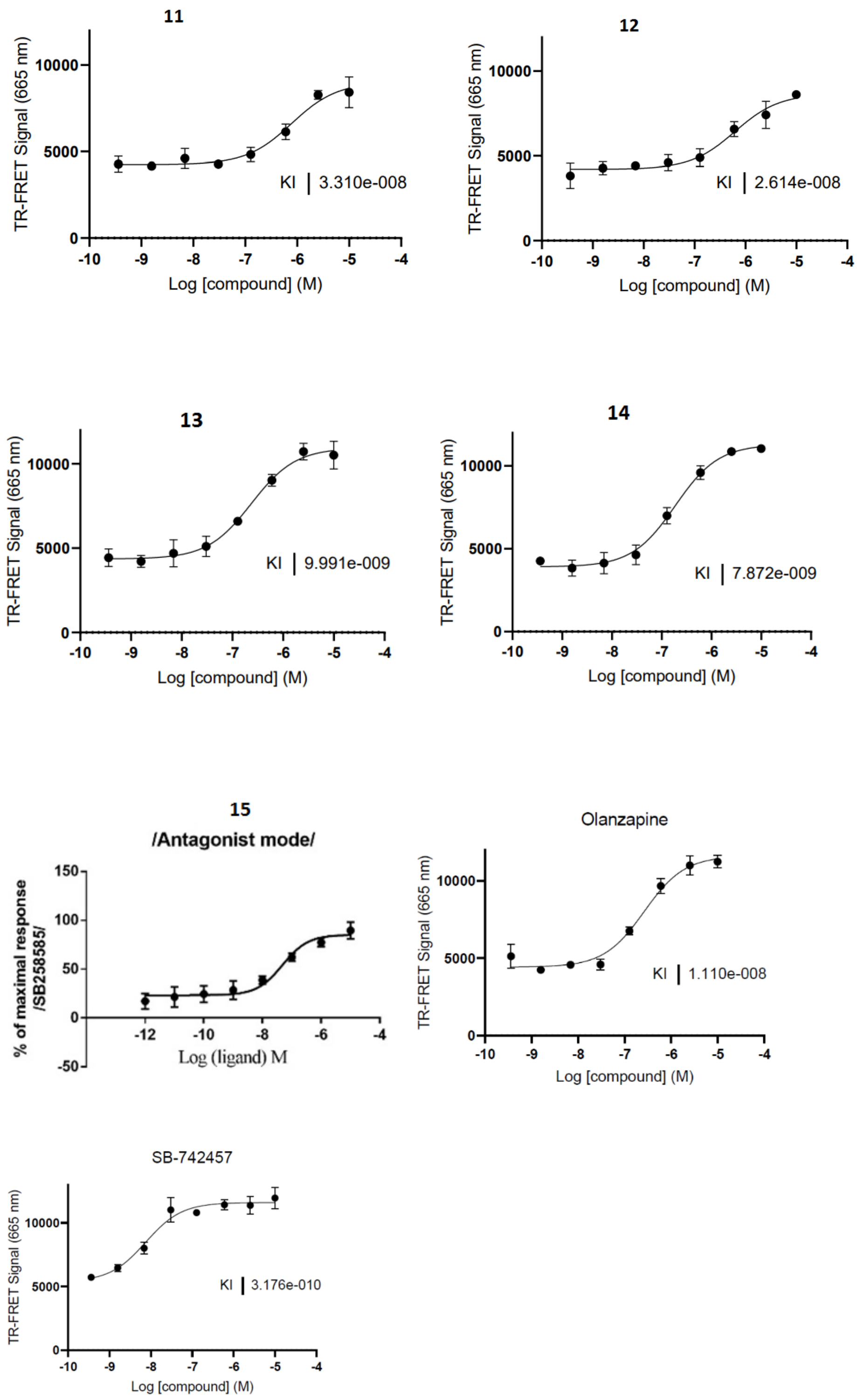

Table S3. cAMP 5-HT6 functional assay for compound 15

Agonist mode*					Antagonist mode**								
	E_{max} % [10^{-5} M]	EC_{50} M	pEC_{50} ± SEM	R2		E max %	IC_{50} M	IC_{50} nM	pIC_{50}	K_b M	pK_b ± SEM	R2 K_b
SEROTONIN	100	2.56E-09	8.60 ± 0.12	0.872	SB258585	100	7.62E-09	7.6	8.12	2.38E-09	8.62 ± 0.02	0.979
**15**	3	n.c.	n.c.	n.c.	**15**	90	4.81E-08	48	7.32	1.50E-08	7.82 ± 0.26	0.913

*Results were normalized as percentage of maximal agonist response (Serotonin 10^{-5} M)

**Results were normalized as percentage of reference antagonist (SB258585 10^{-5} M)

E_{max} is the maximum possible effect

n.c. - not calculable

Table S4. The results of absorbance recorded for the analyzed compounds for all tested concentrations, compared to those for ascorbic acid (AA)

c [μg/mL]	**13**	**14**	**15**	**AA**
10	0.0075	0.0137	0.0310	0.0174
20	0.0365	0.0249	0.0542	0.0445
40	0.0396	0.0491	0.0785	0.0809
80	0.0853	0.0780	0.1153	0.2069
120	0.1141	0.0964	0.1215	0.3376
140	0.1261	0.1222	0.1280	0.4109
180	0.1589	0.1414	0.1739	0.5865
200	0.1618	0.1785	0.2212	0.6803
230	0.2075	0.2549	0.2466	0.7720

Table S5. The ascorbic acid equivalents (%AAE) for compounds 13, 14, and 15 at different concentrations.

Concentration [μg/mL]	AAE [%]		
	**13**	**14**	**15**
10	56.90	21.26	-78.16
20	17.98	44.04	-21.80
40	51.05	39.31	2.97
80	58.77	62.30	44.27
120	66.20	71.45	64.01
140	69.31	70.26	68.85
180	72.91	75.90	72.05
200	76.22	73.76	67.48
230	73.12	66.98	68.06

Table S6. Effect of compound 15 in the EPM test

Treatment	Dose (mg/kg)	Open arms				
		**Time (s)**	**% of time**	**Entries**	**% of entries**	**Distance (cm)**
**vehicle**	0	4.20 ± 0.80	1.49 ± 0.30	1.40 ± 0.20	22.86 ± 8.20	223.20 ± 7.40
**15**	0.3	30.79 ± 7.30	11.83 ± 2.60	4.13 ± 0.90	22.51 ± 4.80	278.75 ± 80.30
	1	25.58 ± 3.00	10.38 ± 1.30	6.17 ± 1.10	27.21 ± 6.00	349.83 ± 60.30
	3	40.83 ± 9.90; $p<0.05$ $F(3,21)=4.3870$; $p<0.05$	16.99 ± 4.20; $p<0.01$ $F(3,21)=4.9048$; $p<0.01$	7.33 ± 1.90; $p<0.05$ $F(3,21)=4.1764$; $p<0.05$	32.11 ± 6.50 $F(3,21)=0.5342$; NS	565.17 ±47.40; $p<0.01$ $F(3,21)=5.1059$; $p<0.01$

Increased open-arm exploration denotes reduced anxiety.

Figures S5-S23 Metabolic stability results.

Spectral analysis for **13**, **14,** and **15** and the predicted I phase metabolites

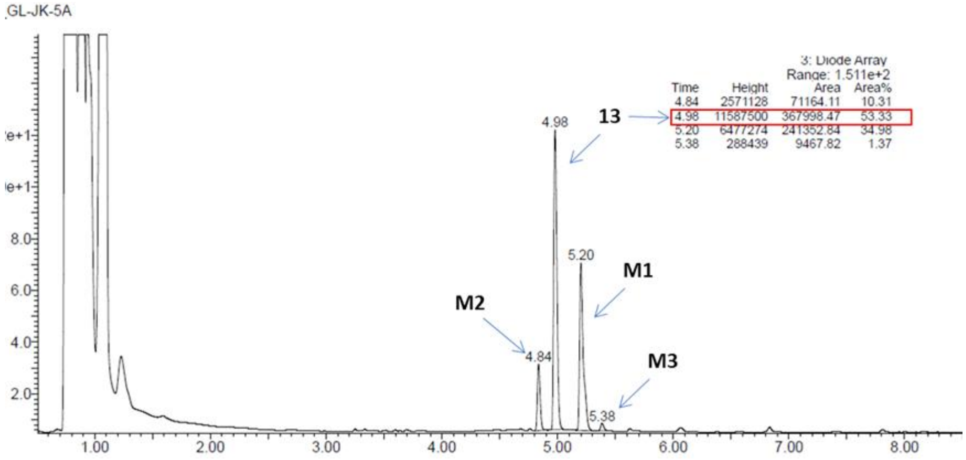

Figure S5. The UPLC of **13** and metabolites (M1 – M3) obtained after 120 min reaction with RLMs.

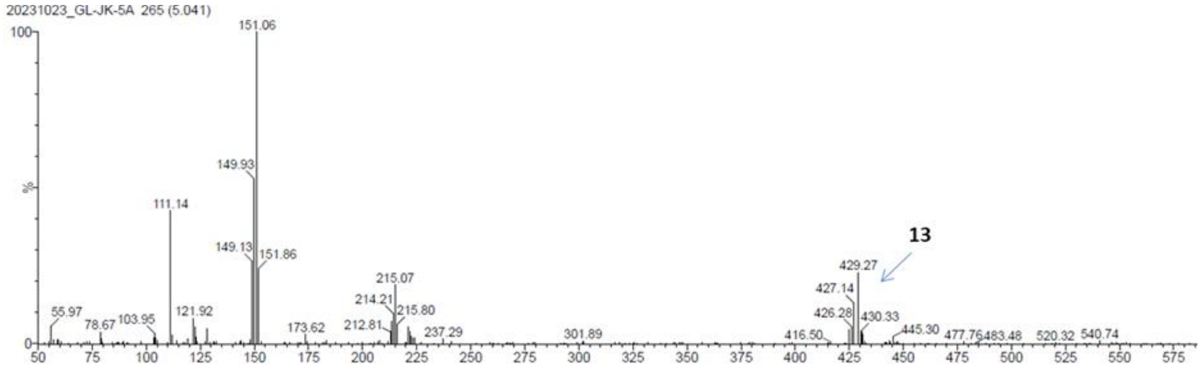

Figure S6. MS spectra of compound **13** obtained after 120 min incubation with RLMs.

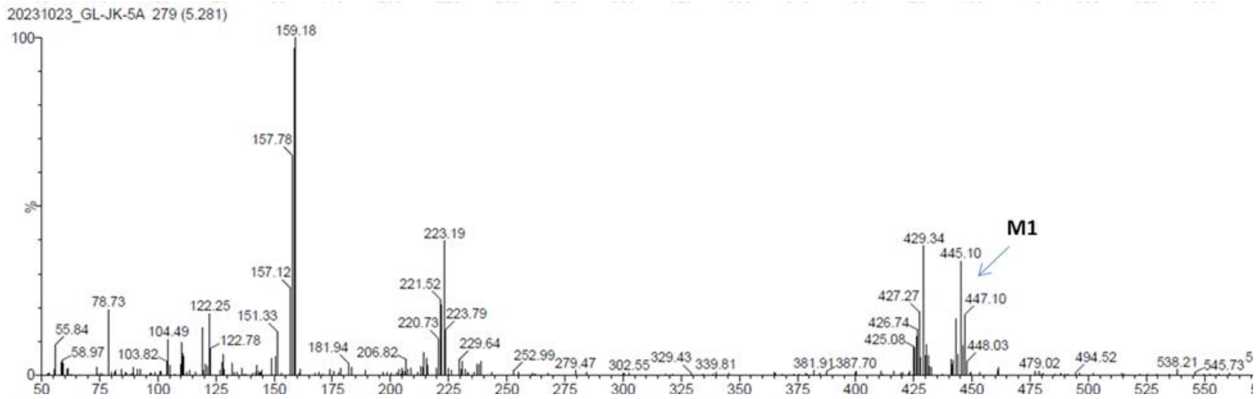

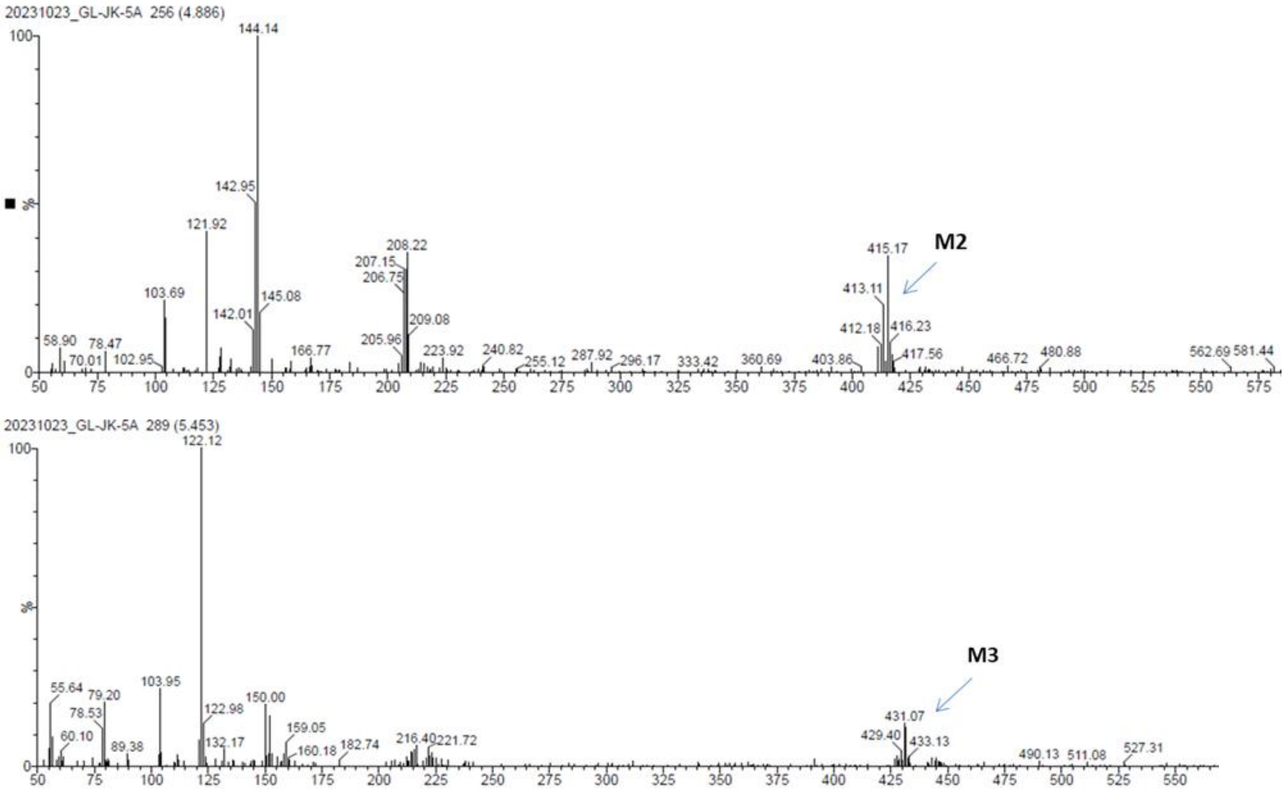

Figure S7. MS spectra and the most probable mass of compound's **13** metabolites M1 – M3 obtained after 120 min incubation with RLMs.

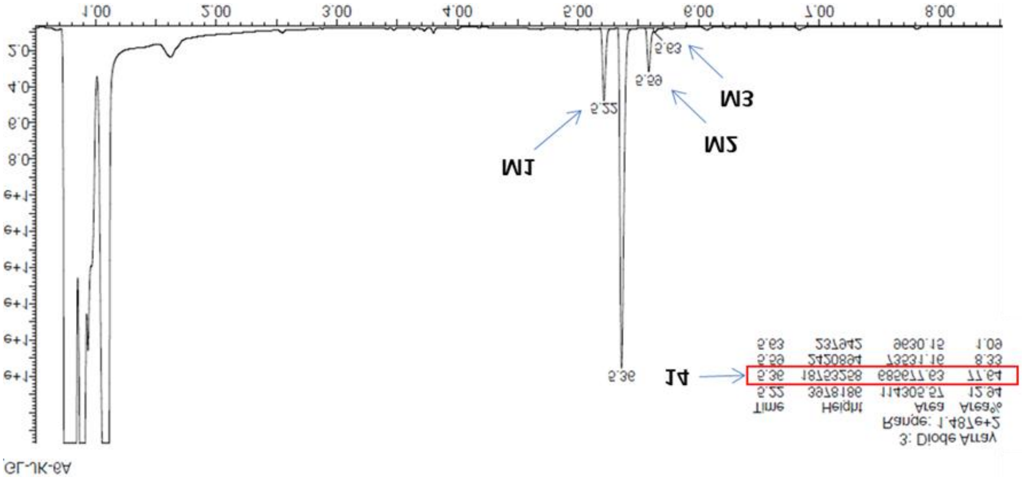

Figure S8. The UPLC of **14** and metabolites (M1 – M3) obtained after 120 min reaction with RLMs.

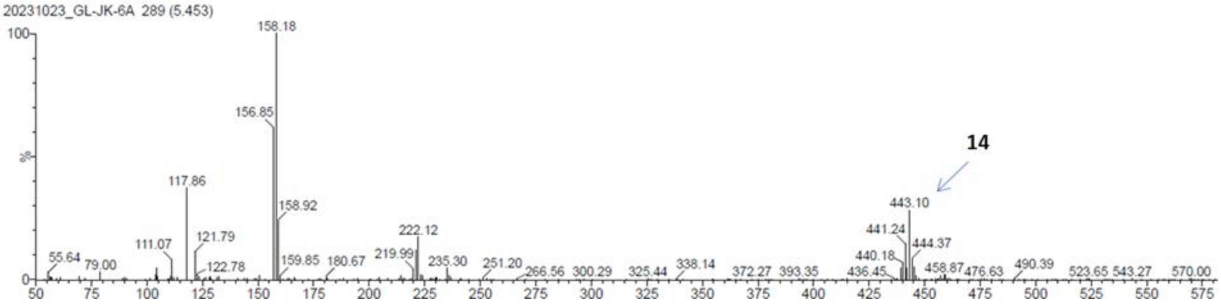

Figure S9. MS spectra of compound **14** obtained after 120 min incubation with RLMs.

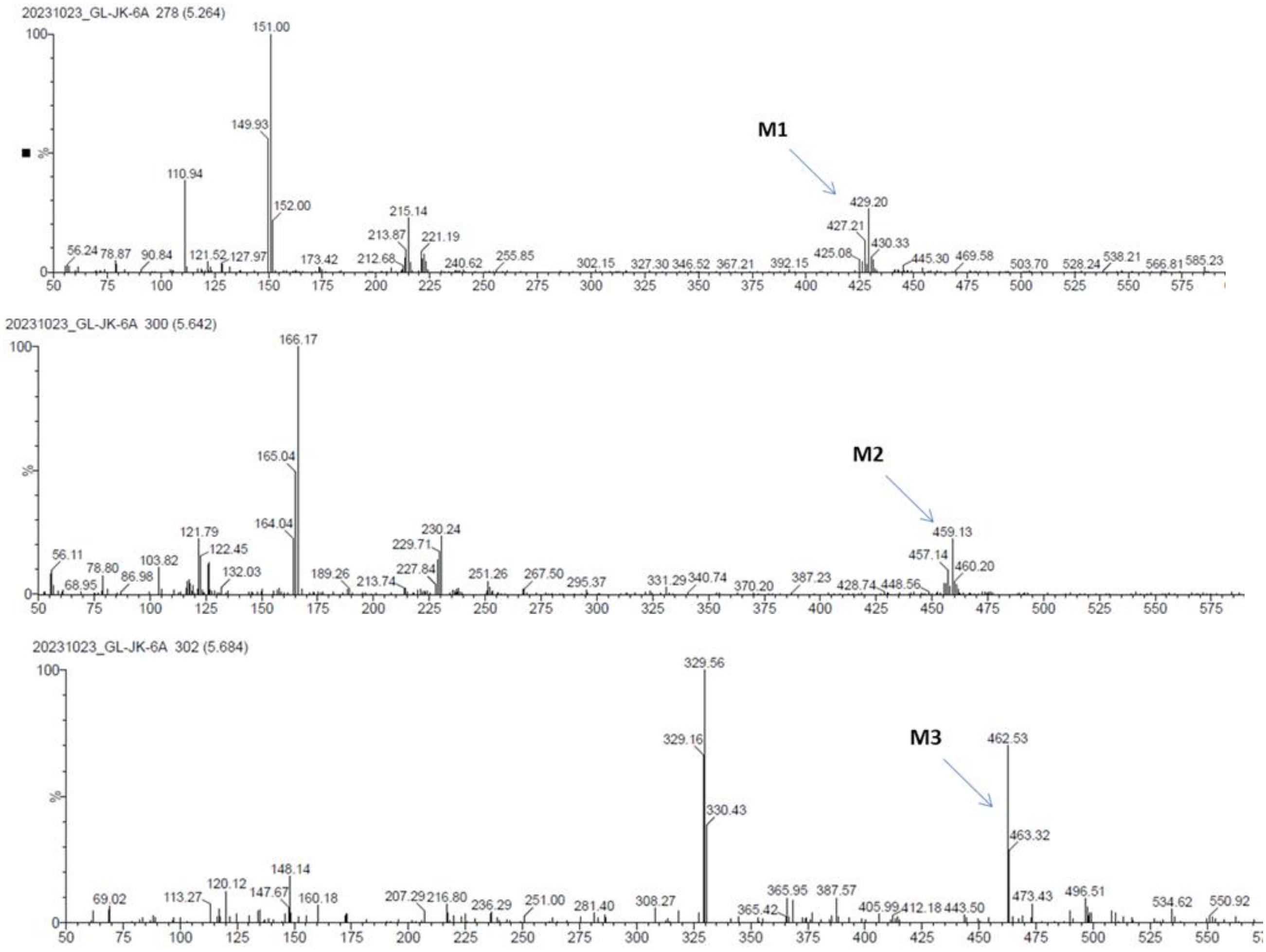

Figure S10. MS spectra and the most probable mass of compound's **14** metabolites M1 – M3 obtained after 120 min incubation with RLMs.

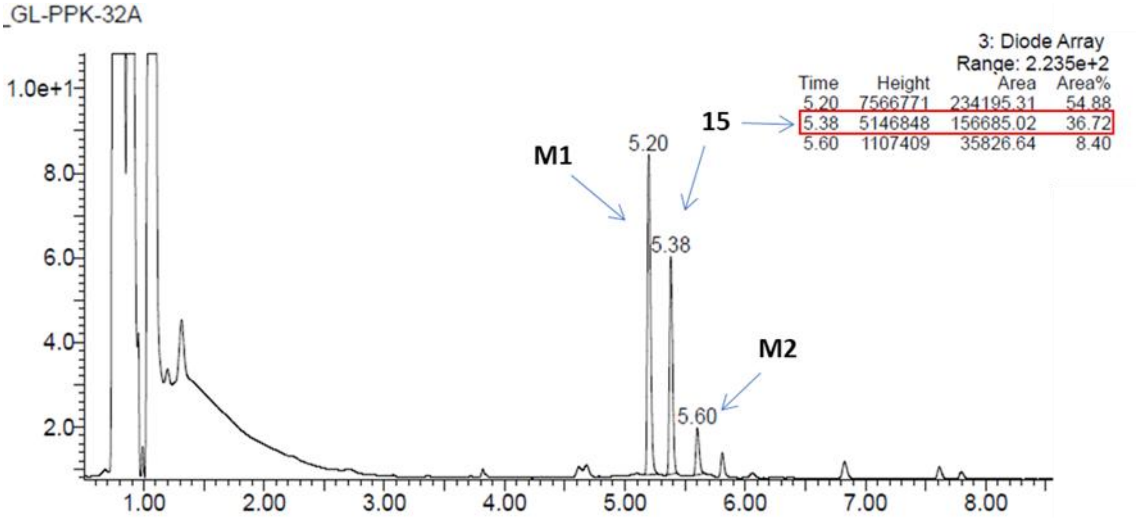

Figure S11. The UPLC of **15** and metabolites (M1 and M2) obtained after 120 min reaction with RLMs.

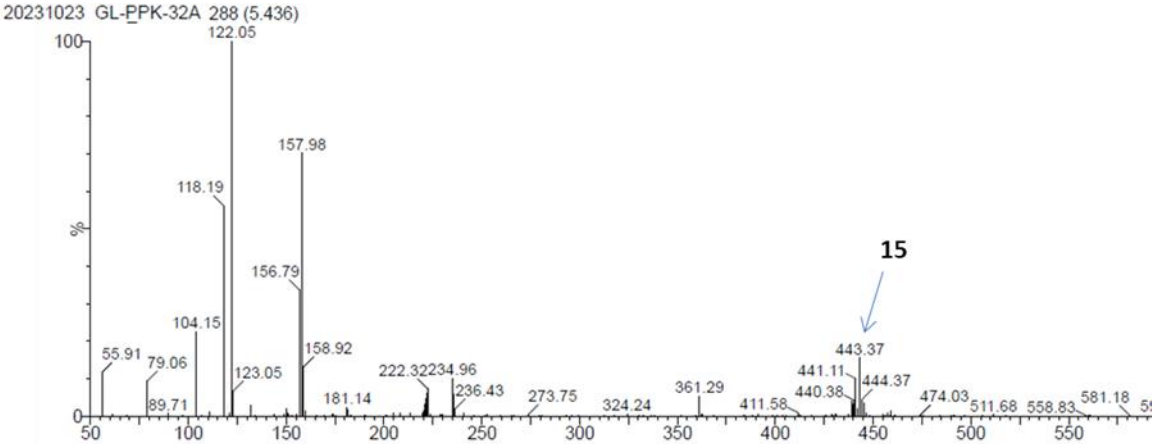

Figure S12. MS spectra of compound **15** obtained after 120 min incubation with RLMs.

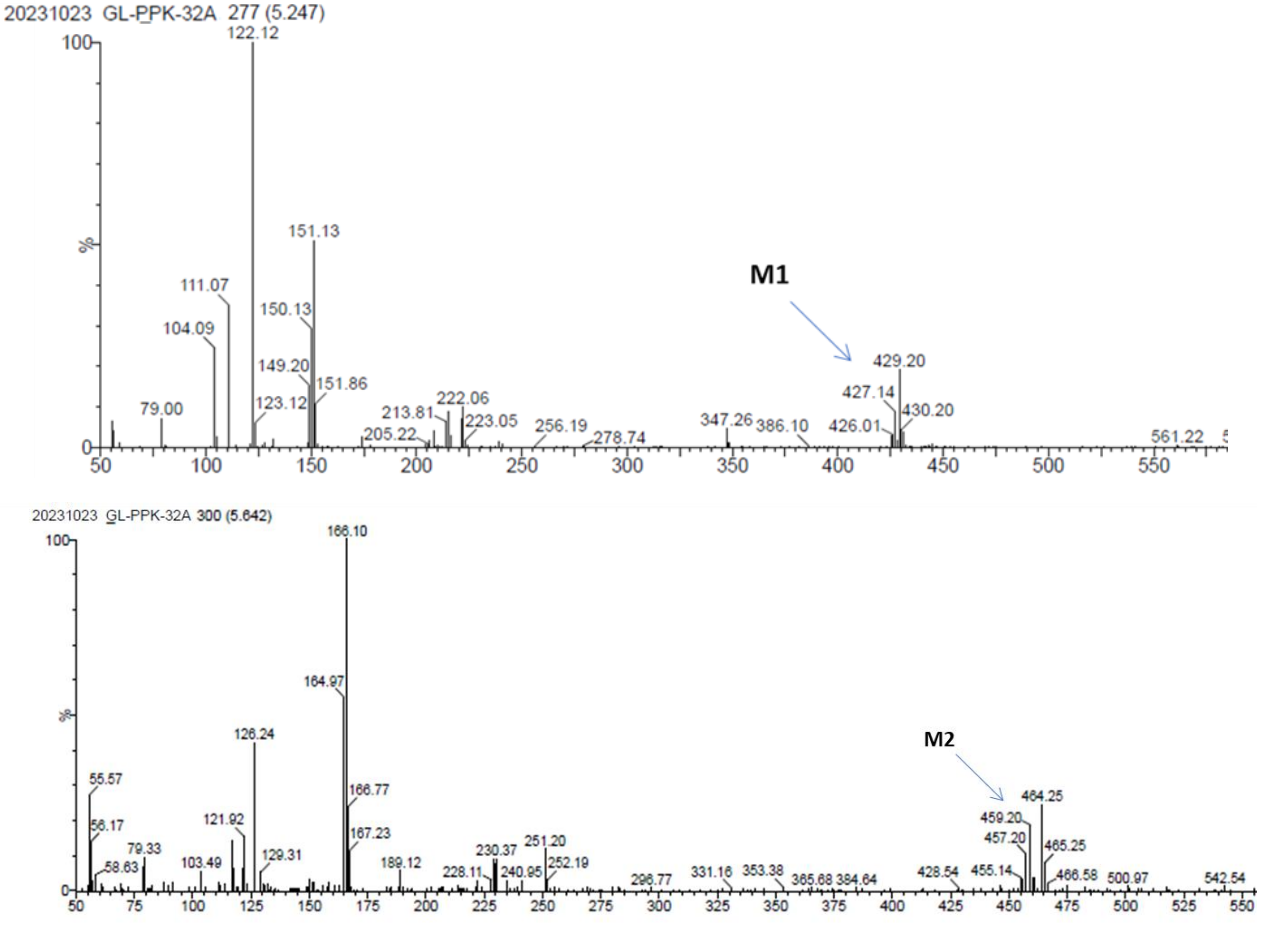

Figure S13. MS spectra and the most probable mass of compound’s **15** metabolites, M1 and M2, obtained after 120 min incubation with RLMs.

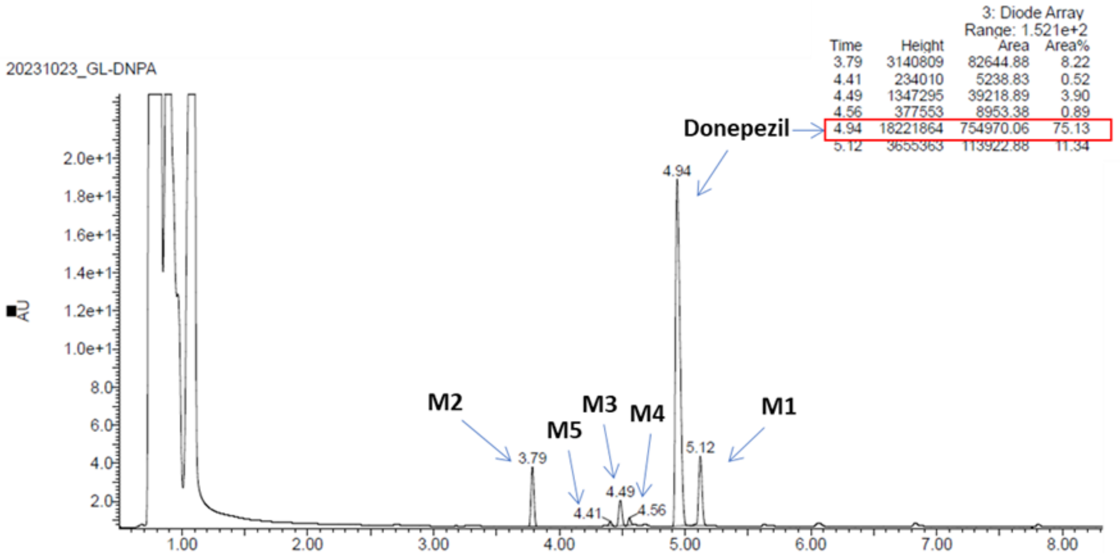

Figure S14. The UPLC of **donepezil** and metabolites (M1 – M5) obtained after 120 min reaction with RLMs.

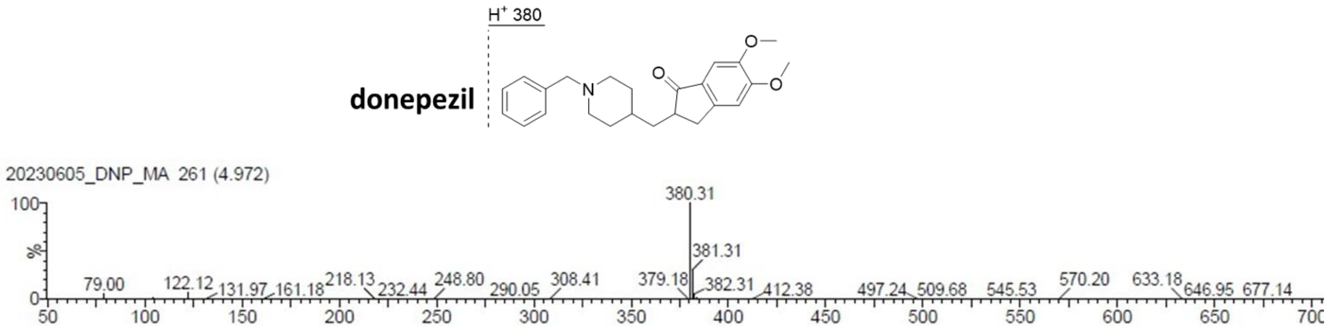

Figure S15. MS spectra of **donepezil** obtained after 120 min incubation with RLMs.

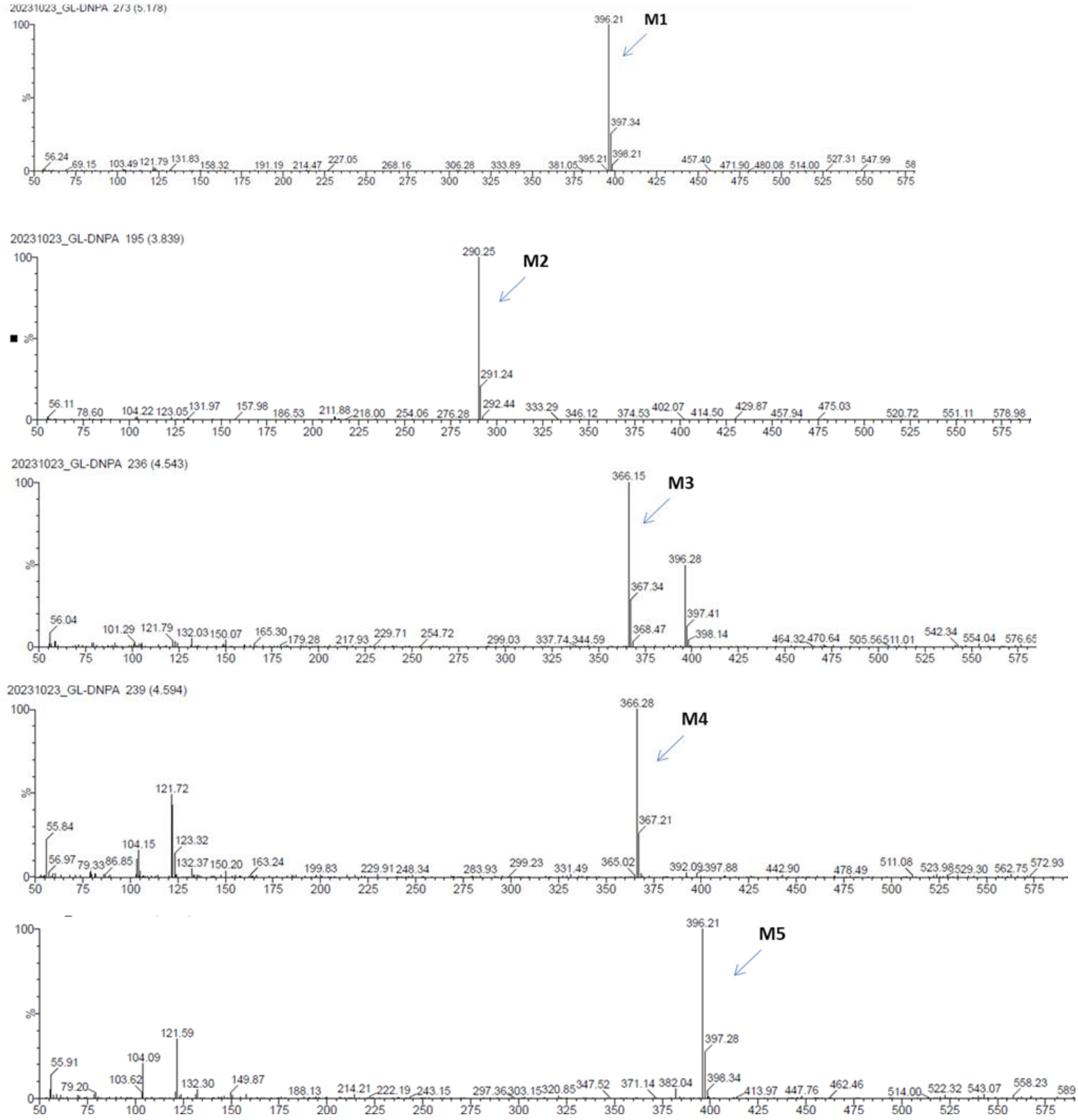

Figure S16. MS spectra and the most probable mass of **donepezil's** metabolites M1 – M5 obtained after 120 min incubation with RLMs.

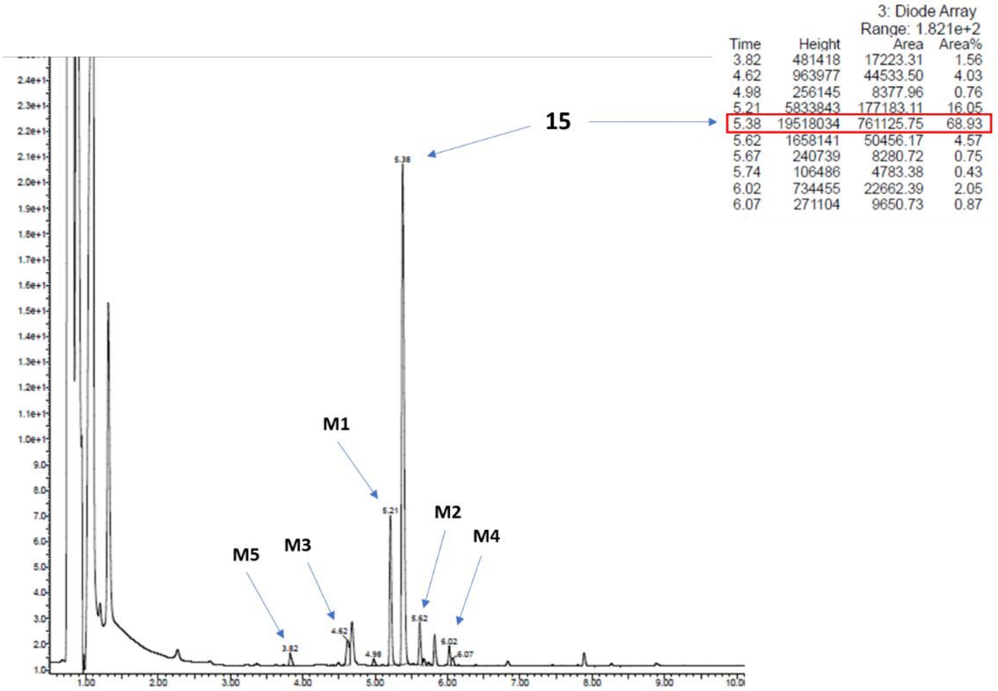

Figure S17. The UPLC of **15** and metabolites (M1- M9) obtained after 120 min reaction with HLMs.

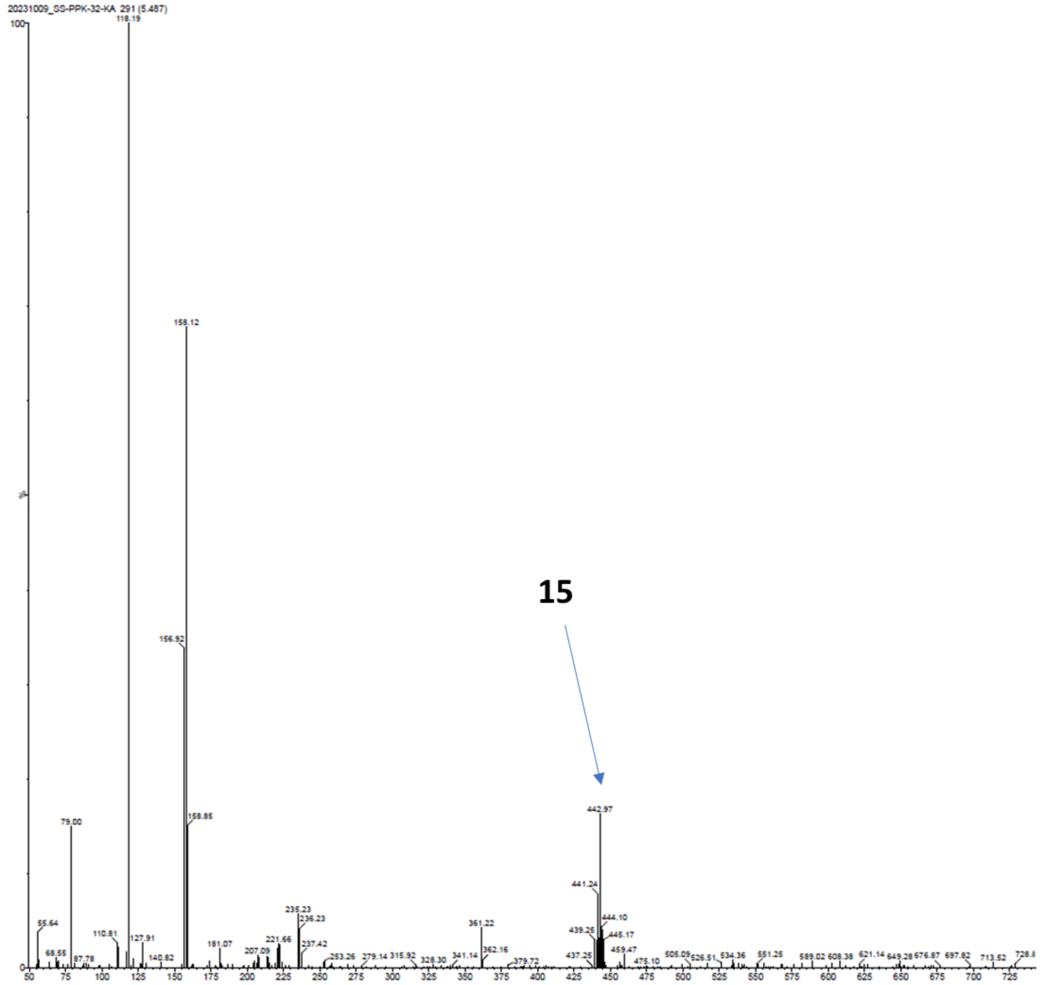

Figure S18. MS spectra of compound **15** obtained after 120 min incubation with HLMs.

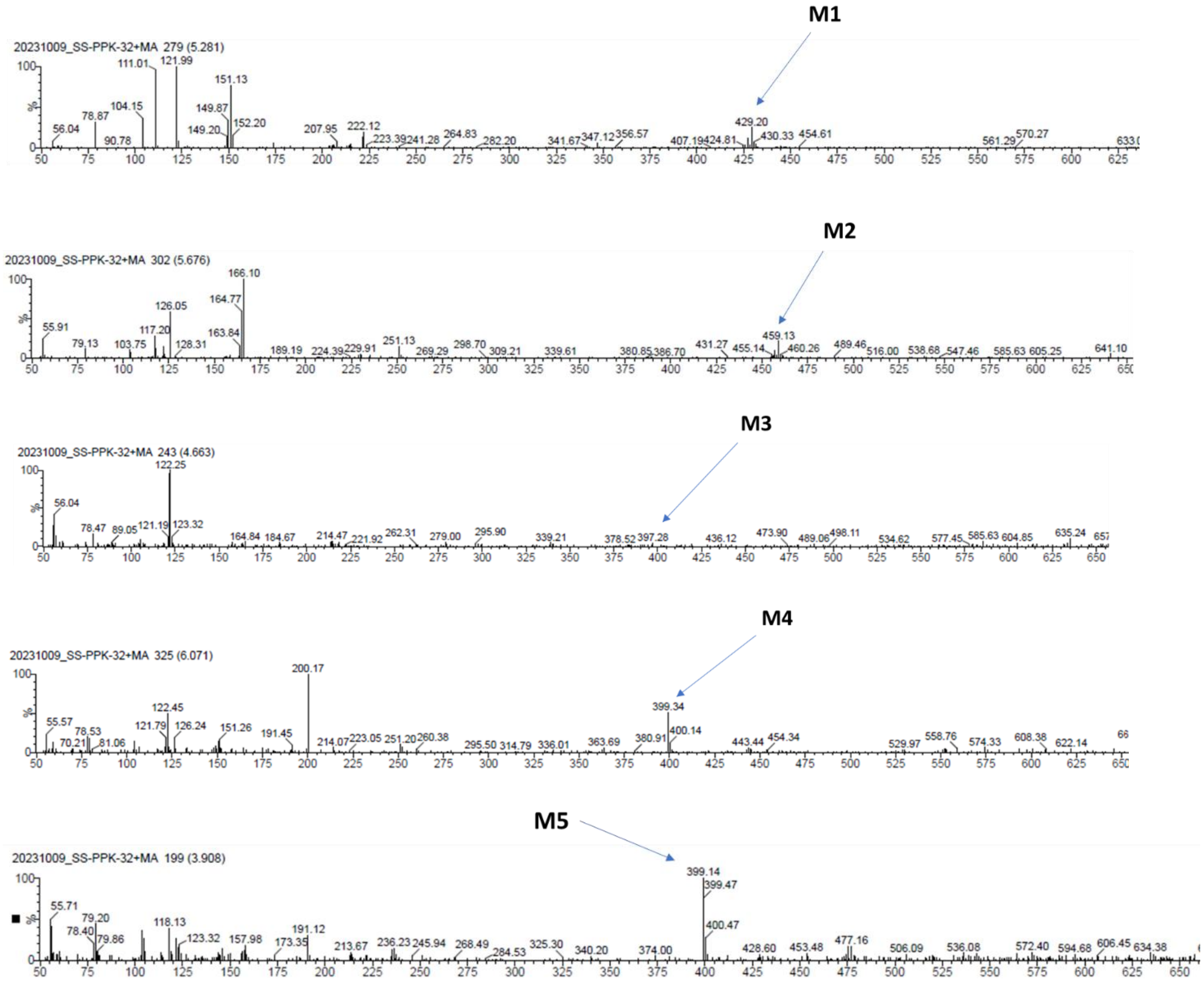

Figure S19. MS spectra and the most probable mass of compound's **15** metabolites, M1 and M2, obtained after 120 min incubation with HLMs.

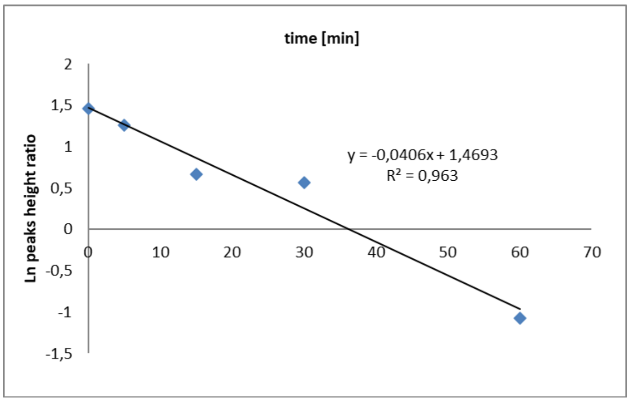

Figure S20. The disappearance in time of compound **15** in the presence of human liver microsomes.

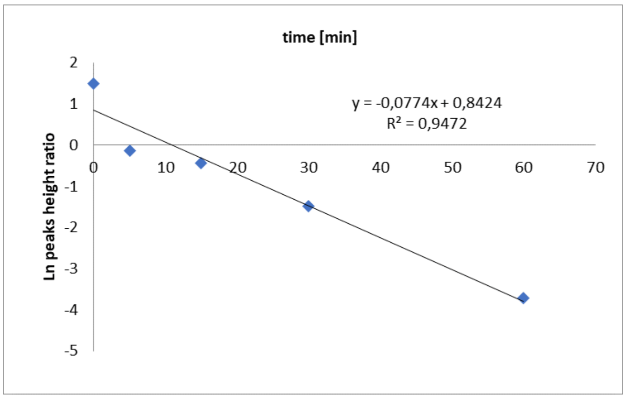

Figure S21. The disappearance in time of compound **15** in the presence of rat liver microsomes.

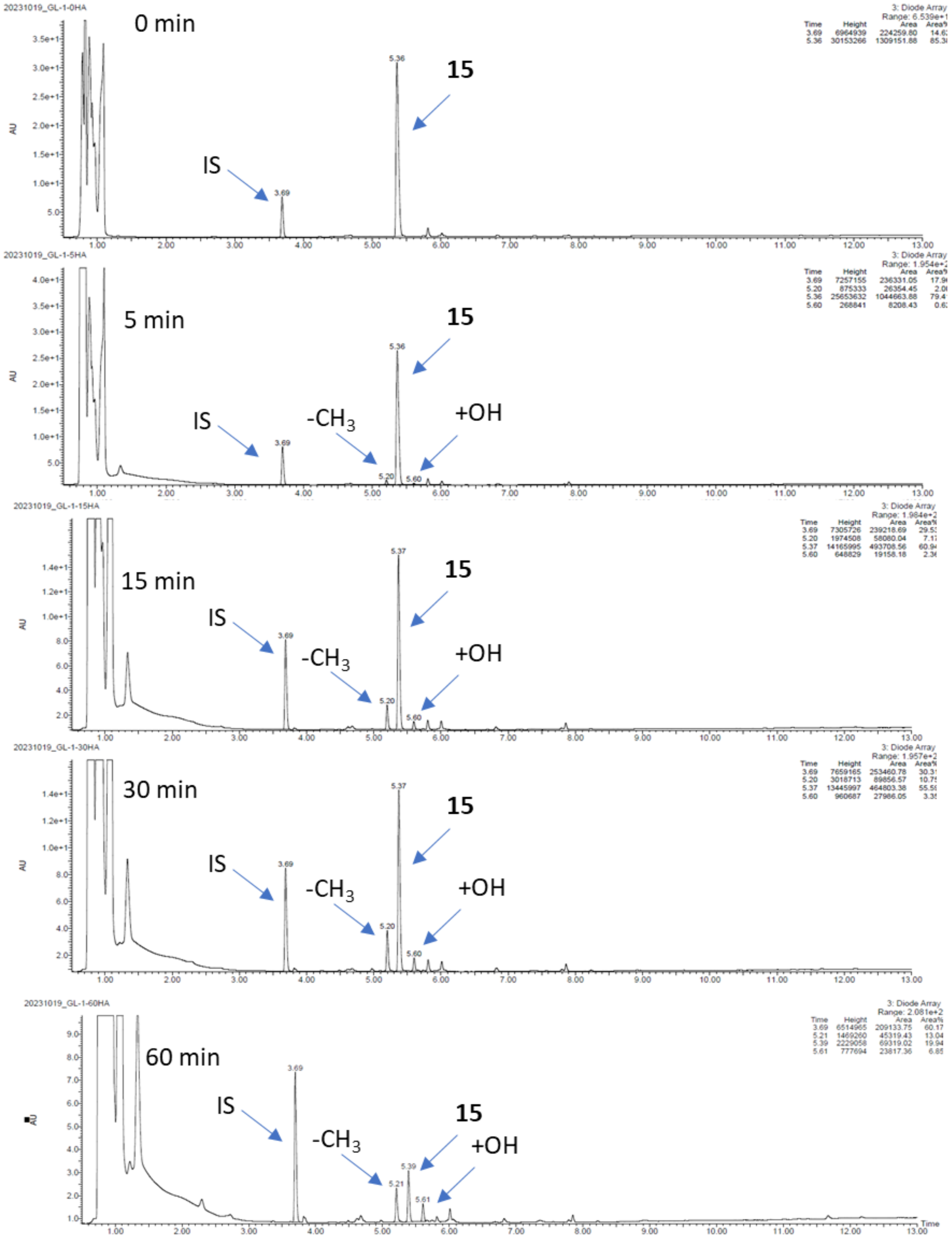

Figure S22. The UPLC of **15** and its disappearance obtained after 60 min reaction with human liver microsomes.

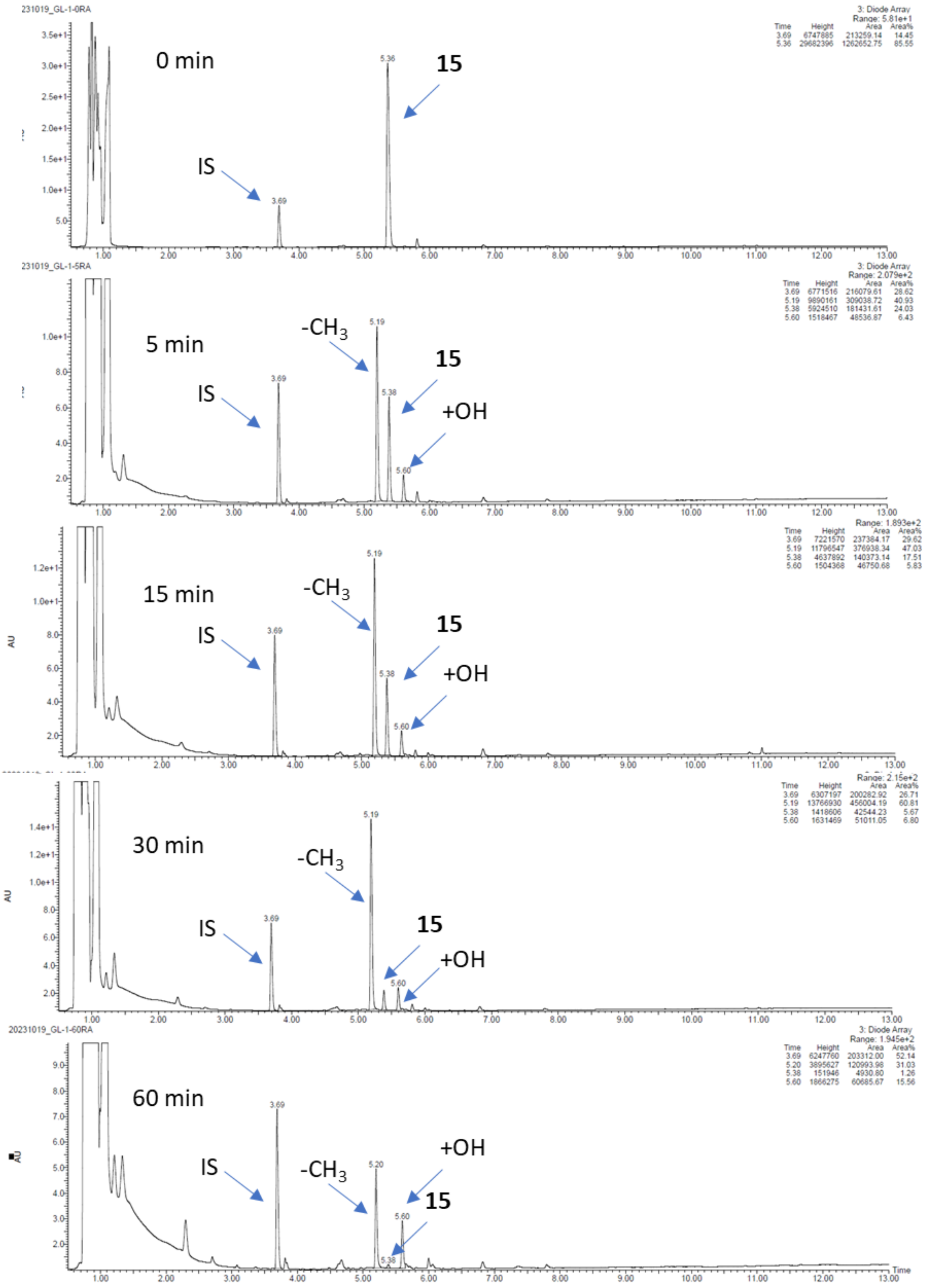

Figure S23. The UPLC of **15** and its disappearance obtained after 60 min reaction with rat liver microsomes.

Table S7. Analysis of a possible cleavage of **13-15** along C(sp3)-Se bounds in the microsome assays, based on LC/MS results based on **Figures S5-S23**.

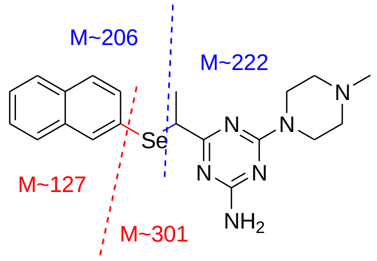

13

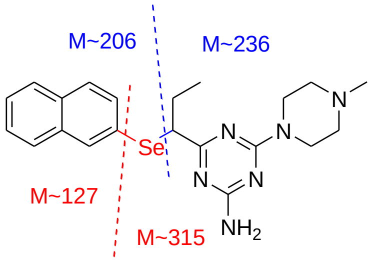

14

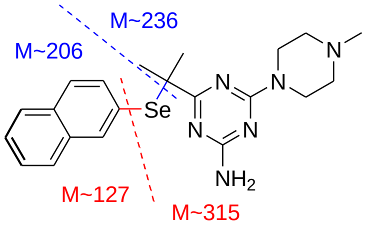

15

**Microsomes**	**Cpd**	**Product**	**M (mass m/z)**					
			**~127**	**206**	**222**	**236**	**301**	**315**
**Rat Liver Microsomes (rLMs)**	**13**	**13**	**A**	**A**	**A**	**A**	**A**	**A**
		**M1**	**A**	**T**	**P**	**A**	**A**	**A**
		**M2**	**A**	**P**	**T**	**A**	**A**	**A**
		**M3**	**A**	**A**	**A**	**A**	**A**	**A**
	**14**	**14**	**A**	**A**	**A**	**A**	**A**	**A**
		**M1**	**A**	**A**	**A**	**A**	**A**	**A**
		**M2**	**A**	**A**	**A**	**A**	**A**	**A**
		**M3**	**A**	**V**	**A**	**V**	**A**	**A**
	**15**	**15**	**A**	**A**	**A**	**V**	**A**	**A**
		**M1**	**A**	**A**	**A**	**A**	**A**	**A**
		**M2**	**T**	**A**	**A**	**A**	**A**	**A**
**Human Liver Microsomes (hLMs)**	**15**	**15**	**V**	**T**	**A**	**T**	**A**	**V**
		**M1**	**A**	**A**	**A**	**A**	**A**	**A**
		**M2**	**A**	**A**	**A**	**A**	**A**	**A**
		**M3**	**A**	**A**	**A**	**A**	**A**	**A**
		**M4**	**A**	**A**	**A**	**A**	**A**	**V**
		**M5**	**A**	**A**	**A**	**T**	**A**	**A**

Occurrence in the MS spectrum: **A**-absent, **P**-present in comparable amount to main mass, **T**- trace amounts in the MS spectrum, **V**- very low trace amounts in the spectrum

An analysis (Table S7) of MS spectra for intact compounds **13-15** and their metabolites in the assays with rLMS (**13-15**) and hLMs (**15**) indicates an occurrence of fragments confirming probable decomposition along C(sp3)-Se bounds. This sensitivity seems to be either structure-dependent or species-dependent. In general, the C(sp3)-Se between Se and the alkyl C (blue line, Table S7) may be seen as more fragile than the Se-phenyl bound (red line). In particular, the mass of products of this decomposition in rLMs visible in MS spectra of metabolites (M1 and M2) of **13** (in the intensity comparable to the mass of the probable metabolites) demonstrates the susceptibility of this bound in **13**. However, the absence of the complementary fragments mass in the corresponding intensity, as well as the presence of the mass of predicted structure of the metabolites (M1 and M2) corresponding to UPLC retention time (and lack of retention time appropriate to Se-C decomposition fragments), indicate that the fragmentation along Se-C (sp3) observed is due to the MS spectrometry conditions rather than due to the oxidative conditions of microsomes.

Analogous products, but in very trace amount, also appears in the MS spectrum of metabolite (M3) of **14**. In the case of compound **15**, the spectra suggest the possibility of decomposition also along the bound with the aromatic moiety. This is particularly visible in the test with hLMs results, where the masses of decomposition products along both Se-C bounds appear in the MS spectrum in (very) low intensity, accompanied by intense signals from the mass of the intact product **15**. Thus, the MS results for **14**, **15** and their metabolites, even more than for **13**, exclude the disintegration of the Se-C(sp3) bond in the oxidative environment of microsomes.

Summing up, the weak signal intensity from the masses of degradation products of **13-15** along the C-Se bounds and their appearance in the MS spectra, together with the mass of expected metabolites (corresponding to UPLC time), indicate this fragmentation as a result of MS spectrometry conditions.

Thus, the high stability along C(sp3)-Se bonds of the highly active 5-HT_6R Se-ligands (**13-15**) in the oxidative conditions of liver microsomes, corresponding to potential therapeutic ones in rats and humans, have been confirmed in our assays.

Figure S24 - Dose-dependent inhibition of hERG channel activity by 15

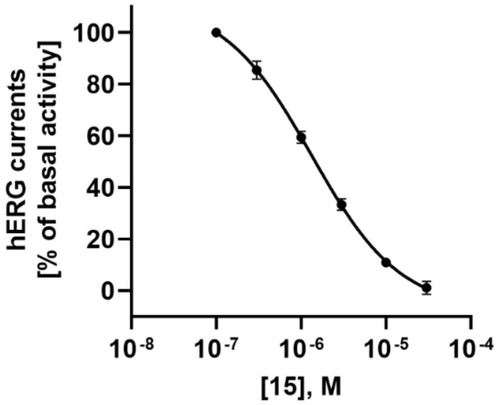

References

1. Sudol, S.; Kucwaj-Brysz, K.; Kurczab, R.; Wilczynska, N.; Jastrzebska-Wiesek, M.; Satala, G.; Latacz, G.; Gluch-Lutwin, M.; Mordyl, B.; Zeslawska, E.; Nitek, W.; Partyka, A.; Buzun, K.; Doroz-Plonka, A.; Wesolowska, A.; Bielawska, A.; Handzlik, J. Chlorine Substituents and Linker Topology as Factors of 5-Ht(6)R Activity for Novel Highly Active 1,3,5-Triazine Derivatives with Procognitive Properties in Vivo. *Eur J Med Chem* **2020,** 203, 112529.
